# Random Copolymers of Styrene with Pendant Fluorophore Moieties: Synthesis and Applications as Fluorescence Sensors for Nitroaromatics

**DOI:** 10.3390/molecules27206957

**Published:** 2022-10-17

**Authors:** Mohamad Zen Eddin, Ekaterina F. Zhilina, Roman D. Chuvashov, Alyona I. Dubovik, Alexandr V. Mekhaev, Konstantin A. Chistyakov, Anna A. Baranova, Konstantin O. Khokhlov, Gennady L. Rusinov, Egor V. Verbitskiy, Valery N. Charushin

**Affiliations:** 1 I. Postovsky Institute of Organic Synthesis, Ural Branch of the Russian Academy of Sciences, S. Kovalevskaya Str. 22, Ekaterinburg 620108, Russia; 2 Chemical Engineering Institute, Ural Federal University, Mira Str. 19, Ekaterinburg 620002, Russia; 3 College of Science, University of Aleppo, Mouhafaza Str., Aleppo 12212, Syria; 4 Institute of Physics and Technology, Ural Federal University, Mira Str. 19, Ekaterinburg 620002, Russia

**Keywords:** pendant copolymers, fluorescence chemosensors, nitroaromatic explosive detection, fluorescence quenching

## Abstract

Five random copolymers comprising styrene and styrene with pendant fluorophore moieties, namely pyrene, naphthalene, phenanthrene, and triphenylamine, in molar ratios of 10:1, were synthesized and employed as fluorescent sensors. Their photophysical properties were investigated using absorption and emission spectral analyses in dichloromethane solution and in solid state. All copolymers possessed relative quantum yields up to 0.3 in solution and absolute quantum yields up to 0.93 in solid state, depending on their fluorophore components. Fluorescence studies showed that the emission of these copolymers is highly sensitive towards various nitroaromatic compounds, both in solution and in the vapor phase. The detection limits of these fluorophores for nitroaromatic compounds in dichloromethane solution proved to be in the range of 10^−6^ to 10^−7^ mol/L. The sensor materials for new hand-made sniffers based on these fluorophores were prepared by electrospinning and applied for the reliable detection of nitrobenzene vapors at 1 ppm in less than 5 min.

## 1. Introduction

The development of new chemosensors and applications thereof for the trace probing of various explosives is an important task for researchers, considering their potential for improving anti-terrorism measures and homeland security [1,2,3]. In addition, these technologies could help to enhance the environmental and forensic investigations associated with explosive substances [4,5]. Accordingly, the design and synthesis of prompt and highly sensitive devices for the detection of explosive compounds is crucial for safeguarding global security. Nitroaromatic compounds (NACs) are the principal ingredients in many explosive blends, and they are also widely exploited by the agrochemical, dye, and pharmaceutical industries. In particular, 2,4,6-trinitrotoluene (TNT); 2,4-dinitrotoluene (2,4-DNT); and 2,4,6-trinitrophenol (picric acid, PA) are widely used in military ammunition, being present in unexploded landmines around the world [6,7,8].

Although many precise techniques exist for NAC recognition and quantification (e.g., surface-enhanced Raman spectroscopy, ion mobility spectrometry, gas/liquid chromatography/mass spectrometry), they are generally quite expensive, time-consuming, and/or lack the portability necessary for real-time application [9,10].

On the other hand, various types of sensors [11], such as biosensors, electrochemical sensors, colorimetric sensors [12], and fluorescent sensors, have been developed and applied for detection purposes. The fluorescence sensor has received the most attention due to its low cost, high response speed, contactless detection capability, and high detection sensitivity [6,7,8,13].

Thanks to their unique optical and electrochemical properties, various polymers have attracted considerable attention from researchers over the last two decades, which has resulted in numerous technological innovations [14,15]. In particular, their implementation in devices for detecting nitroaromatic explosives should be mentioned [16,17,18,19].

Polycyclic aromatic hydrocarbons, for instance, pyrene, can be considered preferred fluorophore units due to their chemical stability and high quantum yields [20,21,22].

Notably, pyrene was efficiently utilized as a fluorescent indicator dopant in polystyrene to detect vapors of nitro explosives at the nanomolecular level [23]. Recently, Akkoc and Karagoz developed pyrene-based polymeric microbeads as fluorescence chemosensors for the highly sensitive and selective detection of 2,4-dinitrotoluene (DNT) up to 1.39 ppb in aqueous media [24].

**Figure 1:**
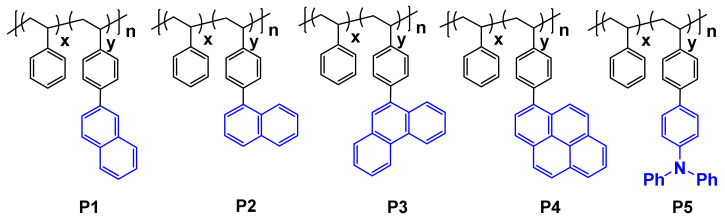
Structure of investigated polymers **P1**–**P5**.

This paper deals with a further extension of the research focused on the design of novel fluorophores for chemosensors. Herein, we report the synthesis of new random copolymers of styrene with pendant fluorophore moieties, such as pyrene, naphthalene, phenanthrene, and triphenylamine (Figure 1), and the systematic investigation of their photophysical properties and application as fluorescent sensors for nitroaromatic explosives.

## 2. Results and Discussion

### 2.1. Synthesis and Thermal Properties

**Scheme 1:**
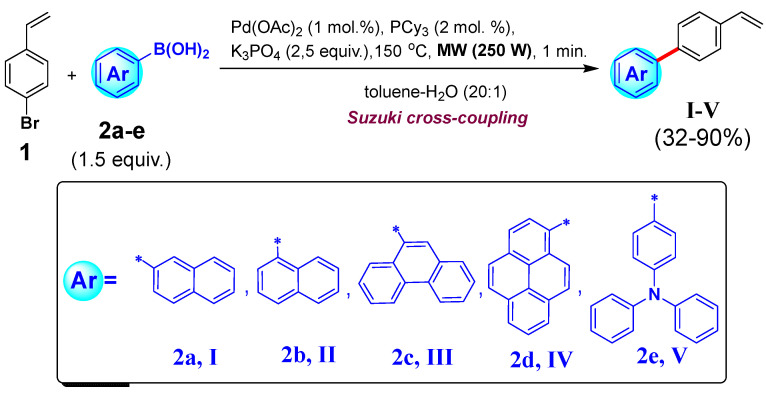
Syntheses of 4-arylstyrenes (**I–V**) through the microwave-assisted Suzuki coupling reaction.

The monomers with pendant fluorophore moieties, 4-arylstyrenes (**I**–**V**), were synthesized by reacting 4-bromostyrene with the corresponding arylboronic acid through the microwave-assisted Suzuki coupling reaction (Scheme 1) [25].

**Scheme 2:**
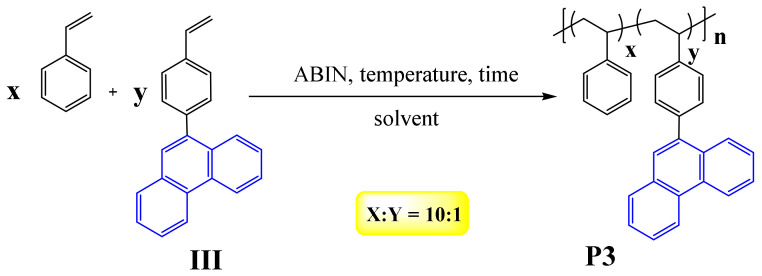
Polymerization of styrene with 4-(9-phenanthrenyl)styrene (**III**).

The polymerization of vinyl monomers is usually carried out in a radical manner by the action of a thermal initiator. As is well-known, 2,2′-azobis(isobutyronitrile) (AIBN) is the key initiator of thermal radicals, and it can be introduced into the reaction in rather small quantities [26]. The polymerization of styrene with 4-(9-phenanthrenyl)styrene (**III**) by the action of AIBN, as the initiator, was recently described for the manufacture of organic field-effect transistor memory materials [27]. We chose this reaction as an appropriate method to prepare the sensitive fluorophores (Scheme 2). The conditions of radical polymerization in THF were optimized to obtain the highest yield of the copolymer and to reach the lowest polydispersity (PDI).

The impact of changing the amount of AIBN and THF, the temperature, and the reaction time was studied (Table 1). When the polymerization took place in toluene, the yield of compound **P3** was two-times lower than that in THF (Table 1, Entry 2). Unfortunately, polymerization under microwave irradiation did not improve the yield of the desired product **P3** (Table 1, Entry 9). The analysis of the results revealed that 1.5 mol.% initiator in 0.5 mL THF at 80 °C for 3 h proved to be the optimal conditions, providing the best yield of polymer **P3** and the lowest polydispersity PDI (see Table 1, Entry 7).

**Scheme 3:**
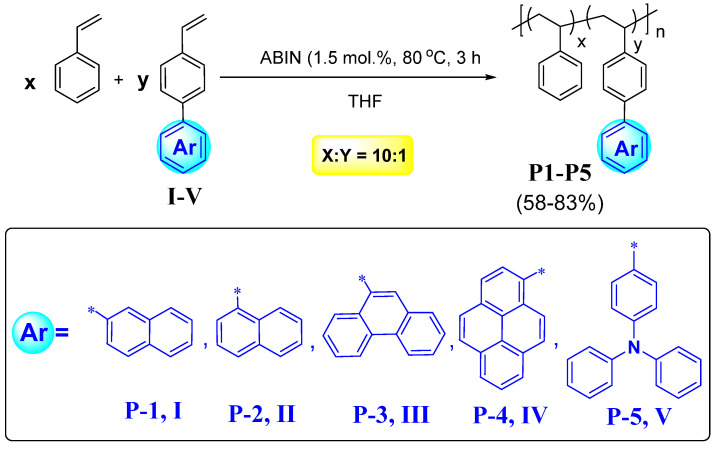
Synthesis of poly[styrene-co-(4-arylstyrene)] (**P1**–**P5**).

The optimum reaction conditions determined above were applied to carry out the copolymerization of styrene with 4-(2-naphthyl)styrene (**I**), 4-(1-naphthyl)styrene (**II**), 4-(1-pyrenyl)styrene (**III**), and 4-(4′-(*N*,*N*-diphenylamino)phenyl)styrene (**V**) to obtain the corresponding copolymers (**P1**–**P5**) in high yields (Scheme 3, Figures S1–S5). The polymerization results and thermal properties are summarized in Table 2.

**Figure 2:**
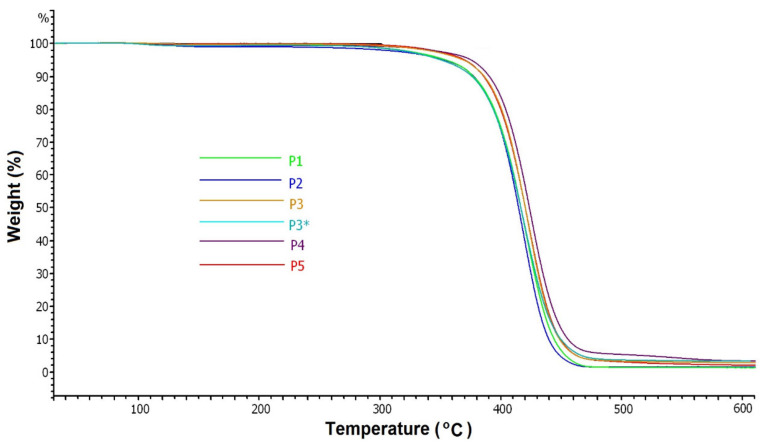
Thermogravimetric curves for polymers **P1**–**P5**.

The thermal stability of the synthesized copolymers was investigated by thermogravimetric analysis (TGA) under argon flow. The T_d_ for all synthesized polymers proved to be higher than 385 °C (Figure 2, Table 2). These data show the good thermal stability of the obtained polymers for applications as sensor materials. According to the shape of the thermogravimetric curves, it is most likely that the thermal decomposition of all the copolymers **P1**–**P5** proceeded along the path of polymer depolymerization, similarly to styrene.

The structures of the obtained vinyl monomers (**I**–**V**) and copolymers (**P1**–**P5**) were also proved by Fourier transform infrared spectroscopy (FTIR) (Table S1, Figures S6–S16). All IR spectra of **I**–**V** contained the bands that are characteristic for vibrations of vinyl groups at ~1626 cm^−1^. In addition, the out-of-plane =CH_2_ wagging vibrations gave rise to a strong band at ~900 cm^−1^. These bands were logically absent in the FTIR spectra of **P1**–**P5**, in which vibrational modes of methylene groups were observed. The FTIR spectra for copolymers **P1**–**P5** were similar to the spectrum of polystyrene [28]. The main difference concerned the presence of CH wagging vibrations among adjacent hydrogens of the aryl substituents in the range of 890–760 cm^−1^.

### 2.2. Photophysical Studies of the Obtained Fluorescent Polymers

The photophysical properties of the prepared polymers **P1**–**P5** were investigated at room temperature using UV–vis and photoluminescence (PL) spectroscopy in dichloromethane (DCM) solutions and in solid state (Table 3, Figure 3 and Figure 4, and Figures S17–S27).

The UV–vis spectra of polymers **P1**–**P5** contained maximum absorption bands at 350–270 nm (ε = 132,600–858,800 M^−1^·cm^−1^), corresponding to the absorption to 4-arylphenyl substituents. An intensive band at 260–240 nm (332,700–1,318,300 M^−1^·cm^−1^) could be attributed to the absorption of the phenyl ring in polystyrene [29]. The long-wavelength absorption maximum was a bathochromic shift in the series **P1** ≈ **P2** < **P3** < **P5, P4** due to the increasing conjunction of the aromatic system.

**Figure 4:**
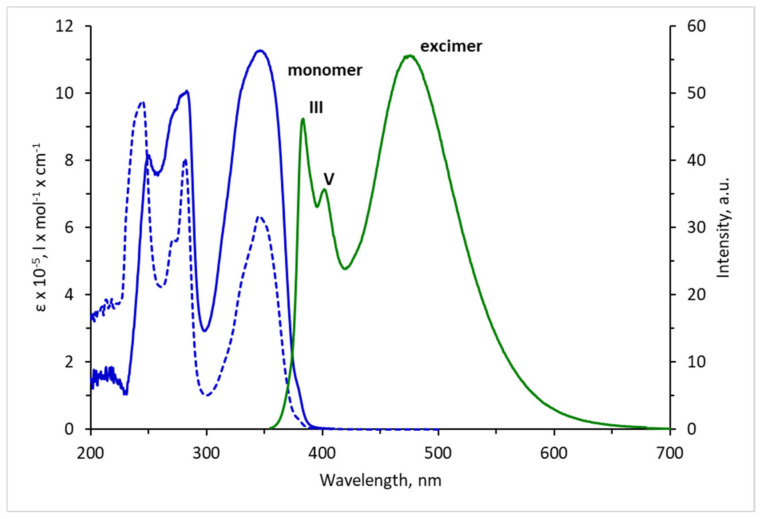
Absorption (--), excitation (–), and emission (–) spectra of **P4** in DCM.

Figure 4 and Figures S17–S21 show the steady-state fluorescence spectra of **P1**–**P5** in DCM solution. All polymers had a fluorescence band in the range of 350–450 nm, which was most likely associated with emission from the monomeric 4-arylphenyl moiety in the polymer chain [25]. The PL spectra of **P1**, **P3**, P3*, and **P4** (though not **P2** and **P5**) had a vibrational structure in this fluorescence band, probably due to steric factors. Figure 4 demonstrates that the PL spectrum of **P4**, in addition to the monomeric structured bands (III, V), showed a longer-wavelength structureless fluorescence band centered at 478 nm, which was attributed to emission from the excimer of pyrene molecules [30].

At the transition from solution to solid powder, the emissions changed noticeably for the **P4** and **P5** polymers. The emission spectrum of each polymer consisted of one band at 457 and 420 nm, respectively.

Tables S2 and S3, as well as Figures S28–S48, demonstrate the fluorescence decay time for all polymers in DCM and solid powders. The experimental results show that the decay process of **P2** and **P5** in DCM consisted predominantly of one fast component, 1.80 ns (92.2%) and 1.09 ns (98.0%), respectively, which could be assigned to 4-arylphenyl substituents. This was also observed in the solid state: τ_1_ = 2.04 ns (97.5%) for **P2** and τ_1_ = 1.08 ns (77.3%) for **P5**. In addition, a small percentage of the short-lived component was observed for **P4**: 1.7–2.2 ns (~8%). The well-known excimer conformation of pyrene in **P4** at 478 nm showed 100% content with τ = 25.23 ns. An increasing average lifetime was observed for the solid powders of **P1**, **P3**, P3*, and **P4**.

### 2.3. Detection of Nitroaromatic Compounds in Dichloromethane Solution

**Figure 5:**
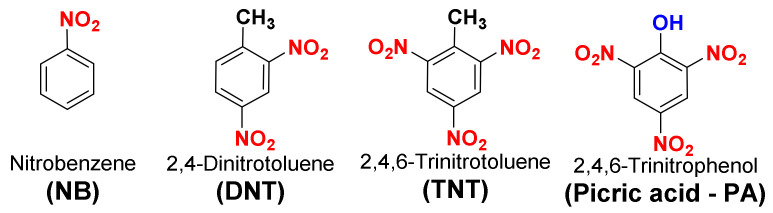
Structures of utilized nitroaromatic quenchers.

**Figure 6:**
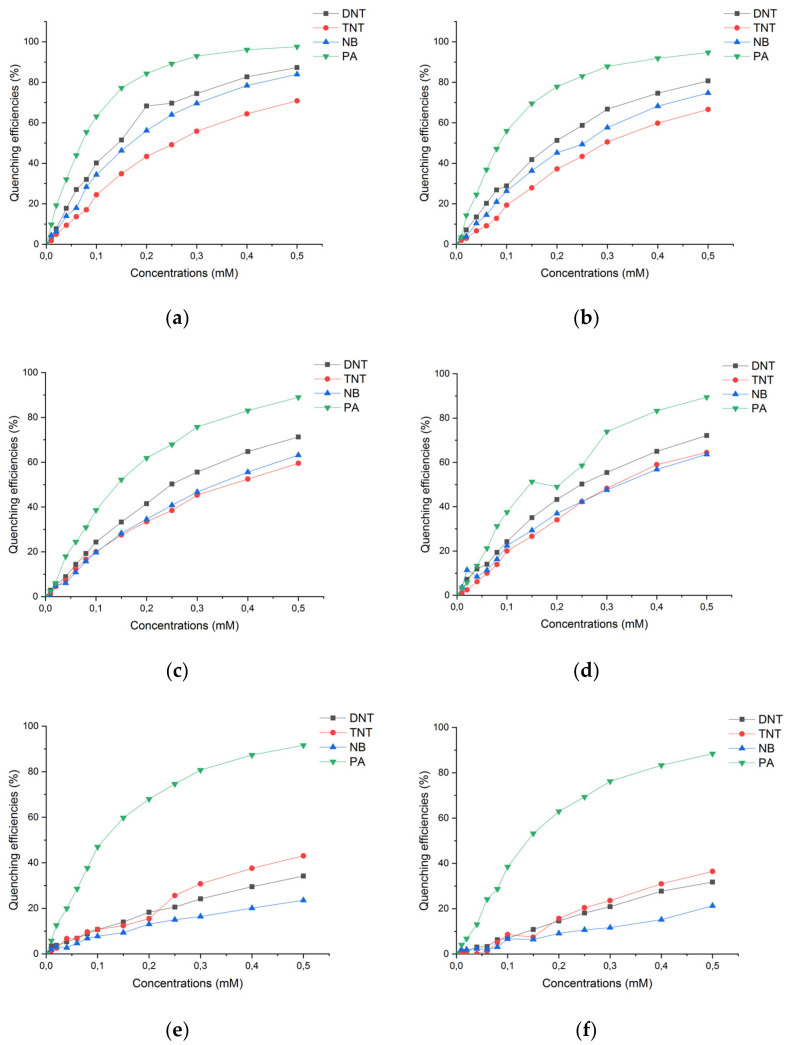
Quenching efficiencies of NB, DNT, TNT, and PA relative to fluorophores **P1** (**a**), **P2** (**b**), **P3** (**c**), P3* (**d**), **P4** (**e**), and **P5** (**f**) at mM level.

Various nitroaromatic compounds, such as nitrobenzene, 2,4-dinitrotoluene, 2,4,6-trinitrotoluene, and picric acid, are not only components of explosives and industrial reagents, but also hazardous ecotoxicants that can cause serious harm to human health (Figure 5). Therefore, we must pay great attention to detecting these dangerous chemicals both in solution and in the vapor phase [31,32,33]. To estimate the ability of the copolymers **P1**–**P5** to detect nitroaromatic analytes, the fluorescence titration of **P1**–**P5** was performed in dichloromethane solution using a method similar to that previously described in [34]. As depicted in Figure 6, all fluorophores **P1**–**P5** acted as efficient detectors of the considered nitroaromatics in solution.

The fluorescence quenching efficiency was quantified, as is typical, by the Stern−Volmer constant (*K*_SV_); the fluorophores showed good linear quenching responses in the low concentration range of NB, DNT, TNT, and PA, as indicated by the regression factors (*R*^2^) being close to 0.99 in most cases (Figures S49–S54). The Stern–Volmer equation is defined as *I*_0_/*I* = 1 + *K*sv × [*Q*], where *I*_0_ and *I* are the fluorescence intensities in the absence and presence of a quencher and [*Q*] represents the quencher concentration. The detection limits (DLs) were determined according to the well-known equation DL = 3σ/*k*, where σ is the standard deviation of the fluorophore intensity in the absence of an analyte and *k* is the slope of the calibration curve [8]. The data on the obtained Stern–Volmer constants and the detection limits for **P1**–**P5** copolymers are summarized in the Table 4.

**Figure 7:**
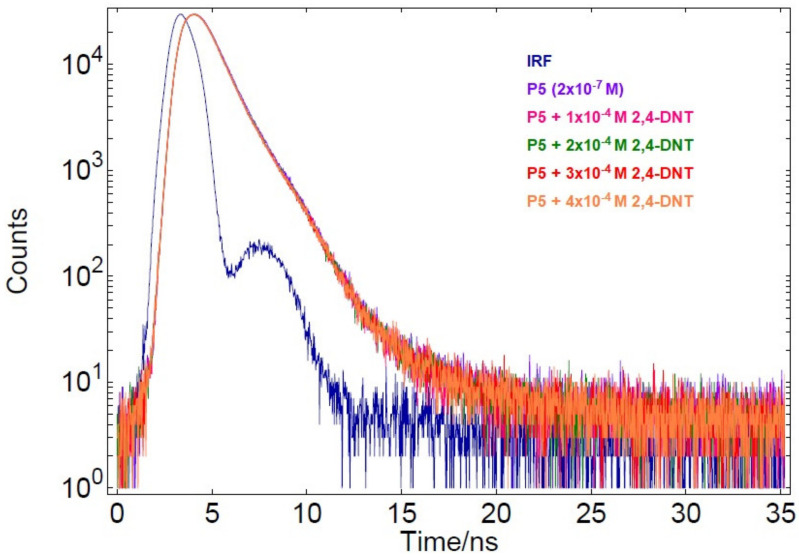
Fluorescence decay time curves of **P5** with DNT in DCM at an excitation wavelength of 300 nm and an emission wavelength of 392 nm.

The linear relationship of the Stern–Volmer plots (in the range of concentrations from 0 to 1 × 10^−4^ M) (Figures S49–S54) and the lifetime measurements for **P5** upon the addition of DNT suggested the predominant role of static interactions in the fluorescence quenching (Figure 7 and Figures S55–S58 and Table S4 in Supplementary Materials). To confirm this suggestion, the quenching rate constant (*k*_q_) value was calculated using the Stern–Volmer equation:

*I*_0_/*I* = 1 + *K*sv × [Q] = 1 + *k*_q_τ_0_[Q]. (1)

**Figure 8:**
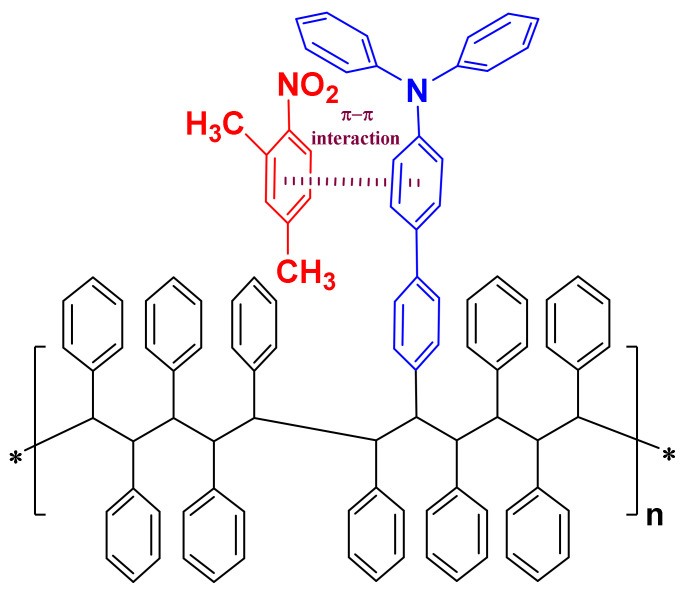
The proposed complexation mechanism of copolymer **P5** with DNT.

Accordingly, the quenching rate constant value could was determined as *k*_q_ = *K*sv/τ_0_, where *τ*_0_ is the average lifetime of the copolymer in the absence of an analyte. For the DNT titrated dichloromethane solution of **P5**, the *K*sv value was found to be 4.66 × 10^4^ M^−1^, and the average lifetime value (τ_0_) was calculated as 1.12 × 10^−9^ s. Evidently, the calculated value of *k*_q_ = 4.16 × 10^13^ was much higher than the maximum scatter collision quenching constant, which is 2 × 10^10^ M^−1^ × s^−1^ for dynamic quenching [35]. Therefore, the detection mechanism was undoubtedly static quenching. Thus, we believe that the formation of the non-fluorescent complex **P5–DNT** took place for the pendant fluorophore moiety due to the π–π interaction, as was previously proved for pyrimidine polymers [33] and push–pull systems [8]. The proposed mechanism of complexation is shown in Figure 8.

### 2.4. Application of Polymers to Detect Nitroaromatic Compounds in Vapor Phase

#### 2.4.1. Preparation and Morphology of Electrospun Sensing Materials

**Figure 9:**
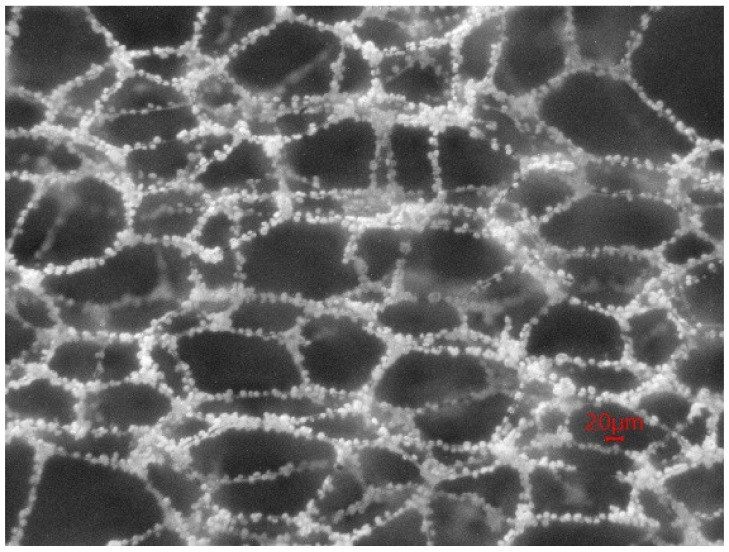
Microphoto of **M1** material electrospun on foam substrate.

The on-site detection of nitroaromatic vapors is critical for security providers and for the protection of human health. The electrospinning technique was used to produce sensing materials based on the synthesized polymers. As is common, polystyrene (PS) was used for the electrospinning, and the process parameters were selected according to the literature [36,37,38]. Melamine formaldehyde foam cut into 2 mm sheets was selected as the substrate for material deposition due to its permeability, low fluorescence under 365 nm UV excitation, chemical inertness, availability, and ease of manipulation. The electrospinning solution was prepared by dissolving the sensor polymer in THF to obtain approximately 500 µL of 5% *w/v* solution. The molecular weight of the polymers was not sufficient to form fibers [36,37,38], and the deposition was achieved by the spraying of polystyrene beads. Sensing materials **M1**, **M2**, and **M3** were prepared from polymers **P4**, **P1**, and **P5**, respectively. All electrospun polymers produced similar structures, featuring beads approximately 10 µm in diameter (8.65 ± 1.99 µm for **P4** (**M1**), 9.48 ± 1.07 µm for **P1** (**M2**), and 10.10 ± 2.72 µm for **P5** (**M3**), see Figure 9 and Figure S59). The shape of the beads resembled structures obtained for 5% solution *w/v* in the literature [36,37,38]. Electrospun copolymers **P3**, P3*, and **P2** emitted negligible fluorescence intensities in a solid state under 365 nm UV irradiation and were not included for further examination. Out of the remaining materials, **M1** was the brightest and **M2** was the dimmest, which was in good agreement with the higher absolute quantum yield in the solid state for copolymer **P4** and the much lower yield for copolymer **P1** (see Table 3). Figure 9 shows the obtained **M1** material under magnification.

#### 2.4.2. Sensor Cartridge and Fluorescence Recorder

**Figure 10:**
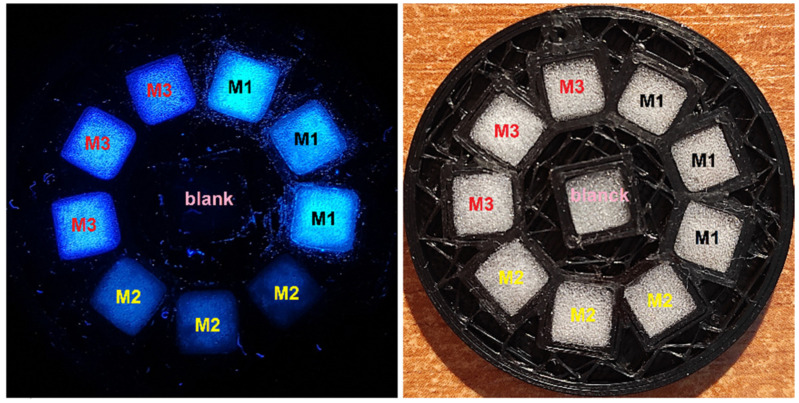
Sensor element cartridge under UV (**left**) and visible (**right**) illumination.

To adapt the sensor materials for gas-phase sampling, a cartridge was manufactured from ABS plastic via 3D printing (Figure 10). The cartridge featured radially positioned sensor material slots with through-holes and was installed perpendicular to the airflow. The remainder of the cartridge’s surface blocked the passage of air to force the airflow through the slots. Foam sheets carrying electrospun sensor materials were cut into fragments and installed into the slots. The cartridge design enabled the housing of an array of materials for the parallel evaluation of their responses to gas-phase analytes.

**Figure 11:**
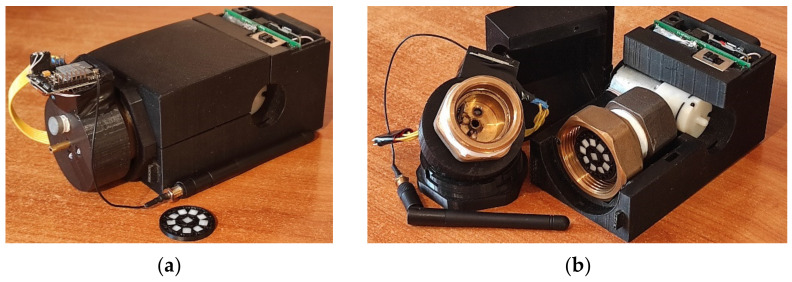
Fluorescence recorder and cartridge used in the experiment: assembled (**a**) and disassembled (**b**).

For the detection of vapors, the cartridge was put into an original fluorescent recorder (Figure 11). The main design idea of this portable fluorescence recorder was the use of a compact camera for the parallel recording of the fluorescence intensities of sensing materials in the visible band (Figure S60). A UV-light-emitting diode (λ_em_ = 365 nm) was applied for the fluorescence excitation of materials on the cartridge (Figure S61). The camera was fitted with a UV-suppressing optical filter to prevent the interference of the excitation light in the fluorescence recording. During the measurement, the video stream was transmitted for processing to the PC. Custom software was used to operate the device and extract fluorescence intensity data from the video stream.

#### 2.4.3. The Sensing Performance of Materials for Various Analytes in Vapor Phase

**Figure 12:**
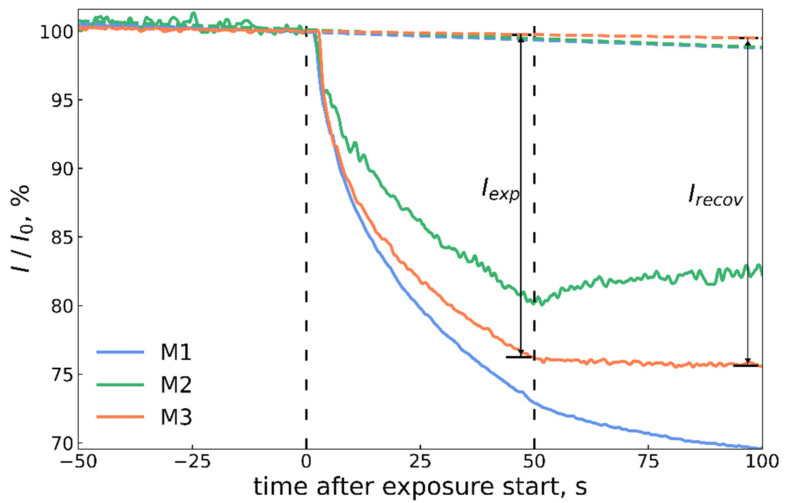
Fluorescence responses of materials **M1**–**M3** towards NB vapors in saturated vapor scenario. Vertical dashed lines mark the vapor exposure interval.

The evaluation of the materials was performed as follows. Two measurement scenarios imitating possible sampling cases were formulated: (1) the detection of saturated vapors accumulated in a limited volume (saturated vapor scenario), and (2) the detection of diluted vapors emanating from a vapor source (diluted vapor scenario). Accordingly, the exposure to analytes was achieved either by inserting the vessel with saturated vapors into the airflow, imitating sampling inside a limited volume, or by dosing saturated vapors into the airflow to produce a constant analyte vapor concentration. The duration of exposure was set to 50 s and 100 s for the two scenarios, respectively. The UV illumination intensity was at a constant level during the test. Figure 12 shows an example of a saturated vapor case record (scenario 1); the 50 s exposure interval is marked by dashed lines. According to the Stern–Volmer equation, the fluorescence attenuation was described by the fluorescence intensity before and during exposure, defined as *I* and *I*_0_, respectively. During the exposure to vapors, *I* was the observed intensity; the base level *I*_0_ had to be predicted. In the experiment, a linear model trained on pre-exposure intensity data was used for *I*_0_ prediction. The primary task of the prediction was to account for the loss of intensity due to the increase in photobleaching over time. Differences between *I* and *I*_0_ at the end of exposure to vapors *I_exp_* and at the end of post-exposure recovery under clean air *I_recov_* were taken as metrics of the sensor response to vapors and are shown in Figure 12.

**Figure 13:**
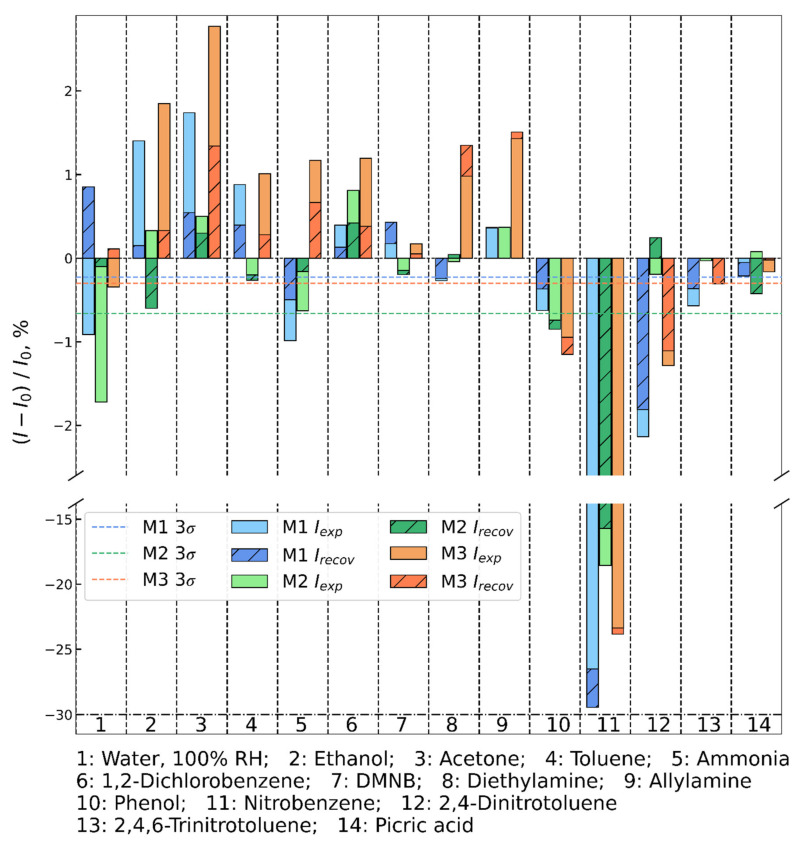
Fluorescence responses of sensor materials **M1**–**M3** towards saturated vapors of interferents and nitroaromatic compounds in a 50 s exposure interval. The 3σ noise levels of registered fluorescence intensity differences are marked with horizontal dashed lines.

The fluorescence intensity responses to saturated vapors of interferents and nitroaromatic analytes are presented in Figure 13. The amplitude of the quenching by the nitroaromatics reflected the saturated vapor pressures of the analytes (372 ppm for NB, 411 ppb for DNT, 9.15 ppb for TNT, and 971 ppt for PA at 25 °C) [39,40]. In the case of analyte vapors, the fluorescence response of the polymers was determined not only by the electron affinity of the analytes towards the fluorophores but also by the permeation dynamics of the vapor molecules into the depths of the polymer. For glassy polymers such as PS, the analyte permeated into the polymer as a saturated front, and the higher vapor pressure allowed for a faster front advance into the interior of the polymer [41]. No significant signal was produced upon exposure to picric acid, presumably due to the saturated vapor pressure at the level below 1 ppb. The material **M1** was capable of detecting saturated TNT vapors at 5.8 ppb from a 160 mL sample volume, and saturated DNT vapors were detected at 282.4 ppb and 386.1 ppb from a 160 mL sample volume by **M1** and **M3**, respectively. All materials produced a signal of at least 30% fluorescence quenching upon exposure to NB. A summary of the detection times for the analytes is provided in Table S5.

To study the selectivity of the sensors **M1**–**M3**, the fluorescence responses towards interferent vapors were evaluated (see columns 1–10 in Figure 13). Sensor materials **M1**–**M3** demonstrated good selectivity for NB compared to the other analytes and NACs (see column 11 in Figure 13), since their quenching sensitivities were much weaker in these vapors than in NB.

To determine the sensing performance for diluted nitro compound vapors, the materials were exposed to NB and DNT according to the diluted vapor scenario 2 (Figure S46). Picric acid and 2,4,6-TNT were not selected for the test regarding the magnitude of the response to saturated vapors because their saturated vapor levels were too low. At this dilution level, 2,4-DNT was registered by **M3**, and NB was registered by **M1** and **M3**. These measurements demonstrated the applicability of the obtained sensory materials for the direct vapor-phase sensing of explosives with the use of a pre-concentrator or via closed packages allowing vapor accumulation.

The lack of fluorescence recovery under clean air after exposure to nitroaromatics was common to all sensor materials. PS is known to have a sorption capacity linked to the π–π interactions of its benzene rings with aromatic analytes, which contributes to the retention of analytes in the polymer, especially those that are weakly volatile and have an electron affinity towards PS moieties [42]. The tendency to accumulate analytes can be utilized for the detection of low concentrations of vapor contaminants within long exposure intervals. In order to simulate the NB detection at the maximum permissible level [43], a 1 ppm concentration was created in the airflow via a syringe driver and a 20 mL syringe containing saturated NB vapors. The sensor cartridge carrying materials **M1** and **M3** was used in the fluorescence recorder to sample the contaminated air; **M2** was ruled out due to low brightness.

The accumulation of nitroaromatic analytes implied that the resulting fluorescence attenuation would grow as the exposure interval duration increased. Therefore, the detection times were calculated as the times required to produce the 3*σ* threshold signal at a certain analyte concentration so that it would be registered by the fluorescence recorder. The signal value was calculated as the difference between *I* and *I*_0_ within the exposure intervals, and the noise was estimated as the difference between *I* and *I*_0_ for the separate measurement under clean air. The brightness of the material dictated the gain used in the video capture, and the 3*σ* threshold was higher for dimmer materials due to noise amplification.

**Figure 14:**
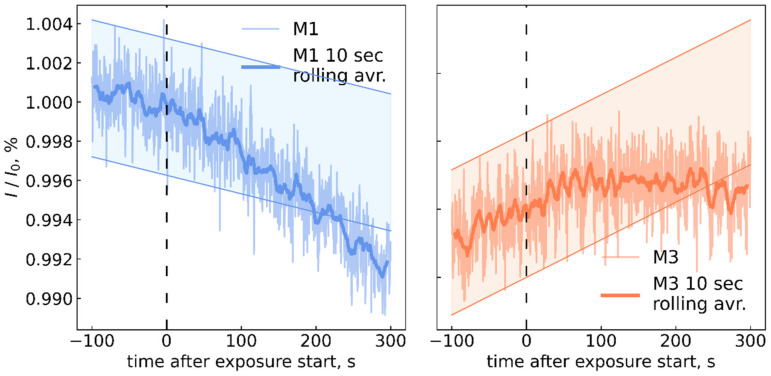
Fluorescence response of materials **M1** and **M3** to NB vapors at 1 ppm concentration.

The 100 s interval before exposure to vapors was used to estimate the expected fluorescence intensity and the noise at the 3*σ* level (Figure 14). The results showed that both materials could detect NB at 1 ppm in less than 5 min and could be applied for air contamination monitoring.

## 3. Experimental Materials and Methods

The detailed specifications of the chemical materials and the methods used for their characterization are provided in the electronic Supplementary Materials.

### 3.1. Materials

Since we have already described the general details of the analytical equipment and methods [8,22,25] used, they are presented in the “***General Information***” section (see Supplementary Materials).

### 3.2. Synthesis of Polymers

The 4-arylstyrene (**I**–**V**) (0.356 mmol), styrene (372 mg, 3.57 mmol), and AIBN (0.9 mg, 1.5 mol.%) were dissolved in 0.5 mL THF. The resulting reaction mixture was stirred under an argon atmosphere and refluxed for 3 h. After cooling to room temperature, the viscous polymer solution was reprecipitated by pouring the solution into a large excess of ethanol three times. The products, poly[styrene-co-(4-arylstyrene)] (**P1**–**P5**), were dried under vacuum overnight.

**Poly[styrene-co-4-(2-naphthyl)styrene] (P1)** was synthesized by the reaction of styrene with 4-(2-naphthyl)styrene (**I**). Yield 420 mg (76%), white powder; ^1^H NMR (500 MHz, CDCl_3_, δ): 7.4–7.9 (CH_2_CHC_6_H_4_C_10_H_7_), 6.4–7.4 (CH_2_CHC_6_H_5_, CH_2_CHC_6_H_4_C_10_H_7_), 1.2–2.1 (CH_2_CHC_6_H_5_, CH_2_CHC_6_H_4_C_10_H_7_); Mw (g∙mol^−1^)/PDI (GPC): 23 × 10^3^/1.7; T_d_ = 387 °C.

**Poly[styrene-co-4-(1-naphthyl)styrene] (P2)** was synthesized by the reaction of styrene with 4-(1-naphthyl)styrene (**II**). Yield 450 mg (82%), white powder; ^1^H NMR (500 MHz, CDCl_3_, δ): 7.3–7.9 (CH_2_CHC_6_H_4_C_10_H_7_), 6.4–7.3 (CH_2_CHC_6_H_5_, CH_2_CHC_6_H_4_C_10_H_7_), 1.3–2.1 (CH_2_CHC_6_H_5_, CH_2_CHC_6_H_4_C_10_H_7_); Mw (g∙mol^−1^)/PDI (GPC): 25 × 10^3^/1.6; T_d_ = 387 °C.

**Poly[styrene-co-4-(9-phenanthrenyl)styrene] (P3)** (and P3*) was synthesized by the reaction of styrene with 4-(9-phenanthrenyl)styrene (**III**). Yield 395 mg (83%) for **P3**, 280 mg (58%) for P3*, white powder; ^1^H NMR (500 MHz, CDCl_3_, δ): 7.4–7.9, 8.6–8.8 (CH_2_CHC_6_H_4_C_14_H_9_), 6.3–7.2 (CH_2_CHC_6_H_5_, CH_2_CHC_6_H_4_C_14_H_9_), 1.2–2.1 (CH_2_CHC_6_H_5_, CH_2_CHC_6_H_4_C_14_H_9_); Mw (g∙mol^−1^)/PDI (GPC): 24 × 10^3^/1.6 for **P3**, 9×10^3^/1.8 for P3*; T_d_ = 392 °C for **P3**, 385 °C for P3*.

**Poly[styrene-co-4-(1-pyrenyl)styrene] (P4)** was synthesized by the reaction of styrene with 4-(1-pyrenyl)styrene (**IV**). Yield 321 mg (78%), white powder; ^1^H NMR (500 MHz, CDCl_3_, δ): 7.8–8.2 (CH_2_CHC_6_H_4_C_16_H_9_), 6.3–7.2 (CH_2_CHC_6_H_5_, CH_2_CHC_6_H_4_C_14_H_9_), 1.2–2.1 (CH_2_CHC_6_H_5_, CH_2_CHC_6_H_4_C_14_H_9_); Mw (g∙mol^−1^)/PDI (GPC): 24 × 10^3^/1.7; T_d_ = 395 °C.

**Poly[styrene-co-4-(4′-(*N*,*N*-diphenylamino)phenyl)styrene] (P5)** was synthesized by the reaction of styrene with 4-(4’-(*N*,*N*-diphenylamino)phenyl)styrene (**V**). Yield 320 mg (80%), white powder; ^1^H NMR (500 MHz, CDCl_3_, δ): 7.2–7.5 (CH_2_CHC_6_H_4_C_18_H_14_N), 6.3–7.2 (CH_2_CHC_6_H_5_, CH_2_CHC_6_H_4_ C_18_H_14_N), 1.2–2.1 (CH_2_CHC_6_H_5_, CH_2_CHC_6_H_4_C_18_H_14_N); Mw (g∙mol^−1^)/PDI (GPC): 26 × 10^3^/1.6; T_d_ = 391 °C.

## 4. Conclusions

We described the synthesis and photophysical properties of five random copolymers comprising styrene and styrene with pendant fluorophore moieties, namely pyrene, naphthalene, phenanthrene, and triphenylamine, in molar ratios of 10:1. It was shown that the emission of these copolymers was highly sensitive towards various nitroaromatic compounds, both in solution and in the vapor phase. The detection limits of these fluorophores for nitroaromatic compounds in dichloromethane solution reached 10^−7^ mol/L. The sensor materials for new hand-made sniffers based on these fluorophores were prepared by electrospinning and applied for the reliable detection of nitrobenzene vapors at 1 ppm in less than 5 min.

## Figures and Tables

**Figure 3:**
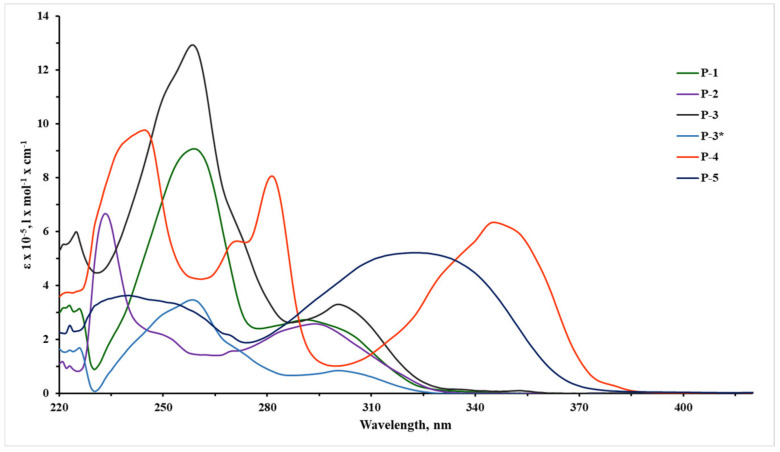
Absorption spectra of **P1**–**P5** in DCM solution.

**Table 1:** Optimization of the copolymerization reaction between styrene and 9-(4-vinylphenyl)phenanthrene (**III**).

Entry	AIBN (mol.%)	Yield (%)	Mw	Mn	PDI
**1**	0.5	52	7800	4200	1.8
**2** ^(**d**)^	0.5	25	3200	2300	1.4
**3**	1.0	60	12,400	7600	1.6
**4**	1.5	70	20,000	11,000	1.8
**5**	2.0	58	9000	5000	1.8
**6** ^(**a**)^	1.5	80	16,000	9800	1.6
**7** ^(**b**)^	1.5	84	24,000	14,400	1.6
**8** ^(**c**)^	1.5	70	31,500	19,100	1.7
**9** ^(**e**)^	1.5	31	11,500	4100	2.8

Number average molecular weight (Mn), weight average molecular weight (Mw), and polydispersity (PDI) were determined by GPC by eluting with THF. ***General conditions*:** monomer **III** (100 mg, 0.356 mmol) and styrene (372 mg, 3.566 mmol) in 5 mL of THF at 80 °C/12 h; ^(a)^ in 2 m L of THF at 80 °C/12 h; ^(b)^ in 0.5 mL of THF at 80 °C/3 h; ^(c)^ in 0.3 mL of THF at 80 °C/2 h. ^(d)^ In toluene. ^(e)^ Reactions under microwave irradiation in 5 mL THF at 100 °C/3 h.

**Table 2:** Polymerization results and thermal properties of **P1**–**P5**.

Copolymer	Yield, %	Mw	Mn	PDI	T_d_, °C
**P1**	76	23,000	13,500	1.7	387
**P2**	82	25,000	15,500	1.6	387
**P3**	83	24,000	14,400	1.6	392
P3*	58	9000	5000	1.8	385
**P4**	73	24,000	14,000	1.7	395
**P5**	80	26,000	16,000	1.6	391

***General conditions***: 4-arylstyrenes **I**–**V** (100 mg,1 equiv.), styrene (10 equiv.), and AIBN (1.5 mol.%) in 0.5 mL THF at 80 °C/3 h.

**Table 3:** Optical properties of polymers **P1**–**P5**.

Polymer	UV Absorption		Fluorescence							
	Solution in DCM						Solid			
	λ_abs_^max^ (nm)	ε_max_ (M^−1^ cm^−1^)	Excitation λ_ex_ (nm)	Emission λ_em_ (nm)	τ _avg_ (ns)	*Φ*_F_ *^a^*	Excitation λ_ex_ (nm)	Emission λ_em_ (nm0	τ _avg_ (ns)	*Φ*_F_ *^c^*
**P1**	292	310,000	290	362	21.19	0.17	305	362	41.25	0.44
	259	1,031,000	263	345	21.60			355	40.31	
								346	40.02	
**P2**	294	309,000	294	358	2.06	0.28	311	359	2.15	0.48
	230	1,271,700	230							
**P3**	301	336,800	301	376	14.57	0.08	351	377	27.88	0.24
	258	1,318,300	265	359	14.74		313	364	27.95	
P3*	301	132,600	301	376	14.41	0.07	351	377	27.53	0.19
	258	517,300	258	359	14.36		313	364	27.21	
**P4**	345	693,000	345	478	25.23	0.29 *^b^*	375	457	23.50	0.93
	281	858,800	281	401	11.46					
	271	585,100	271	383	11.35		350			
	245	1,025,300	245							
**P5**	322	552,300	325	392	1.12	0.30	350	420	1.32	0.77
	246	332,700	246							

*^a^ Φ*_F_ values were determined relative to 2-aminopyridine in 0.1N H_2_SO_4_ as standard (*Φ*_F_ = 0.60); excitation at 300 nm; *^b^* excitation at 345 nm. *^c^ Φ*_F_ values were determined using integrating sphere SC-30 of FS5 Edinburgh Instruments spectrofluorometer.

**Table 4:** Stern–Volmer constants and detection limits of nitroaromatics for fluorophores **P1-P5** in CH_2_Cl_2_.

Polymer	*K*sv × 10^4^, M^−1^/DL, mol × L^−1^			
	PA	TNT	DNT	NB
**P1**	405.79/1.74 × 10^−7^	24.32/4.02 × 10^−7^	34.33/3.70 × 10^−7^	52.26/1.92 × 10^−7^
**P2**	89.16/5.11 × 10^−7^	19.91/3.63 × 10^−7^	41.78/3.44 × 10^−7^	29.56/3.71 × 10^−7^
**P3**	40.25/3.55 × 10^−7^	14.70/4.51 × 10^−7^	24.81/2.58 × 10^−7^	17.14/2.91 × 10^−7^
P3*	42.36/1.18 × 10^−6^	18.15/2.19 × 10^−7^	25.88/2.11 × 10^−7^	17.51/2.39 × 10^−7^
**P4**	54.21/4.78 × 10^−7^	7.56/6.63 × 10^−7^	5.20/7.18 × 10^−7^	3.08/1.04 × 10^−6^
**P5**	76.24/1.74 × 10^−7^	5.74/1.54 × 10^−7^	4.66/3.71 × 10^−7^	2.70/5.55 × 10^−7^

## Data Availability

The data presented in this study are available on request from the corresponding author and co-authors.

## Supplementary Materials

The following supporting information can be downloaded at: https://www.mdpi.com/article/10.3390/molecules27206957/s1. General Information; Figure S1: ^1^H NMR (500 MHz, CDCl_3_) spectrum of **P1**; Figure S2: ^1^H NMR (500 MHz, CDCl_3_) spectrum of **P2**; Figure S3: ^1^H NMR (500 MHz, CDCl_3_) spectrum of **P3**; Figure S4: ^1^H NMR (500 MHz, CDCl_3_) spectrum of **P4**; Figure S5: ^1^H NMR (500 MHz, CDCl_3_) spectrum of **P5**; Figure S6: IR spectrum of **I**; Figure S7: IR spectrum of **II**; Figure S8: IR spectrum of **III**; Figure S9. IR spectrum of **IV**; Figure S10: IR spectrum of **V**; Figure S11: IR spectrum of **P1**; Figure S12: IR spectrum of **P2**; Figure S13: IR spectrum of **P3**; Figure S14: IR spectrum of **P3***; Figure S15: IR spectrum of **P4**; Figure S16: IR spectrum of **P5**; Table S1: IR characteristic frequencies of **I–V** and **P1–P5**; Figure S17: Absorption (*blue*), fluorescence emission (*green*) and fluorescence excitation (*blue dashed*) spectra of compound **P1** in CH_2_Cl_2_; Figure S18: Absorption (*blue*), fluorescence emission (*green*) and fluorescence excitation (*blue dashed*) spectra of compound **P2** in CH_2_Cl_2_; Figure S19: Absorption (*blue*), fluorescence emission (*green*) and fluorescence excitation (*blue dashed*) spectra of compound **P3*** in CH_2_Cl_2_; Figure S20: Absorption (*blue*), fluorescence emission (*green*) and fluorescence excitation (*blue dashed*) spectra of compound **P3** in CH_2_Cl_2_; Figure S21: Absorption (*blue*), fluorescence emission (*green*) and fluorescence excitation (*blue dashed*) spectra of compound **P5** in CH_2_Cl_2_; Figure S22: Excitation (*green*) and emission (*blue*) spectra of solid powder **P1**; Figure S23: Excitation (*green*) and emission (*blue*) spectra of solid powder **P2**; Figure S24: Excitation (*green*) and emission (*blue*) spectra of solid powder **P3**; Figure S25: Excitation (*blue*) and emission (*green*) spectra of solid powder **P3***; Figure S26: Excitation (*green*) and emission (*blue*) spectra of solid powder **P4**; Figure S27: Excitation (*green*) and emission (*blue*) spectra of solid powder **P5**; Table S2: Detailed data of the fluorescence lifetime measurements of **P1-P5** in DCM: τ – lifetime, f - fractional contribution, τ_avg_ – average lifetime, χ^2^ - chi-squared distribution; Figure S28: Time-resolved fluorescence lifetime decay profile of **P1** (*green*), instrumental response function (IRF, *blue*). λ_ex_ = 300 nm, λ_ex_ = 362 nm; Figure S29: Time-resolved fluorescence lifetime decay profile of **P1** (*green*), IRF (*blue*). λ_ex_ = 300 nm, λ_ex_ = 345 nm; Figure S30: Time-resolved fluorescence lifetime decay profile of **P2** (*green*), IRF (*blue*). λ_ex_ = 300 nm, λ_ex_ = 358 nm; Figure S31: Time-resolved fluorescence lifetime decay profile of **P3** (*green*), IRF (*blue*). λ_ex_ = 300 nm, λ_ex_ = 376 nm; Figure S32: Time-resolved fluorescence lifetime decay profile of **P3** (*green*), IRF (*blue*). λ_ex_ = 300 nm, λ_ex_ = 359 nm; Figure S33: Time-resolved fluorescence lifetime decay profile of **P3*** (*green*), IRF (*blue*). λ_ex_ = 300 nm, λ_ex_ = 376 nm; Figure S34: Time-resolved fluorescence lifetime decay profile of **P3*** (*green*), IRF (*blue*). λ_ex_ = 300 nm, λ_ex_ = 359 nm; Figure S35: Time-resolved fluorescence lifetime decay profile of **P4** (*green*), IRF (*blue*). λ_ex_ = 300 nm, λ_ex_ = 478 nm; Figure S36: Time-resolved fluorescence lifetime decay profile of **P4** (*green*), IRF (*blue*). λ_ex_ = 300 nm, λ_ex_ = 401 nm; Figure S37: Time-resolved fluorescence lifetime decay profile of **P4** (*green*), IRF (*blue*). λ_ex_ = 300 nm, λ_ex_ = 383 nm; Figure S38: Time-resolved fluorescence lifetime decay profile of **P5** (*green*), IRF (*blue*). λ_ex_ = 300 nm, λ_ex_ = 392 nm; Table S3: Detailed data of the fluorescence lifetime measurements of solid powders **P1-P5**: τ – lifetime, f - fractional contribution, τ_avg_ – average lifetime, χ^2^ - chi-squared distribution; Figure S39: Time-resolved fluorescence lifetime decay profile of solid **P1** (*green*), instrumental response function (IRF, *blue*). λ_ex_ = 300 nm, λ_ex_ = 362 nm; Figure S40: Time-resolved fluorescence lifetime decay profile of solid **P1** (*green*), instrumental response function (IRF, *blue*). λ_ex_ = 300 nm, λ_ex_ = 355 nm; Figure S41: Time-resolved fluorescence lifetime decay profile of solid **P1** (*green*), instrumental response function (IRF, *blue*). λ_ex_ = 300 nm, λ_ex_ = 346 nm; Figure S42: Time-resolved fluorescence lifetime decay profile of solid **P2** (*green*), instrumental response function (IRF, *blue*). λ_ex_ = 300 nm, λ_ex_ = 359 nm; Figure S43: Time-resolved fluorescence lifetime decay profile of solid **P3** (*green*), instrumental response function (IRF, *blue*). λ_ex_ = 300 nm, λ_ex_ = 377 nm; Figure S44: Time-resolved fluorescence lifetime decay profile of solid **P3** (*green*), instrumental response function (IRF, *blue*). λ_ex_ = 300 nm, λ_ex_ = 364 nm; Figure S45: Time-resolved fluorescence lifetime decay profile of solid **P3*** (*green*), instrumental response function (IRF, *blue*). λ_ex_ = 300 nm, λ_ex_ = 377 nm; Figure S46: Time-resolved fluorescence lifetime decay profile of solid **P3*** (*green*), instrumental response function (IRF, *blue*). λ_ex_ = 300 nm, λ_ex_ = 364 nm; Figure S47: Time-resolved fluorescence lifetime decay profile of solid **P4** (*green*), instrumental response function (IRF, *blue*). λ_ex_ = 300 nm, λ_ex_ = 457 nm; Figure S48: Time-resolved fluorescence lifetime decay profile of solid **P5** (*green*), instrumental response function (IRF, *blue*). λ_ex_ = 300 nm, λ_ex_ = 420 nm; Figure S49: Fluorescence quenching studies of **P1** (1.0 × 10^−5^ mol/L) recorded in the presence of various amounts of NB (*a*), DNT (*b*), TNT (*c*), and PA (*d*), of which 290 nm was taken as the excitation wavelength. The Stern–Volmer plots as function of NB (*e*), DNT (*f*), TNT (*g*), and PA (*h*) concentration in CH_2_Cl_2_, with an excitation wavelength of 290 nm for **P1** solution; Figure S50: Fluorescence quenching studies of **P2** (1.0 × 10^−5^ mol/L) recorded in the presence of various amounts of NB (*a*), DNT (*b*), TNT (*c*), and PA (*d*), of which 294 nm was taken as the excitation wavelength. The Stern–Volmer plots as function of NB (*e*), DNT (*f*), TNT (*g*), and PA (*h*) concentration in CH_2_Cl_2_, with an excitation wavelength of 294 nm for **P2** solution; Figure S51: Fluorescence quenching studies of **P3** (1.0 × 10^−5^ mol/L) recorded in the presence of various amounts of NB (*a*), DNT (*b*), TNT (*c*), and PA (*d*), of which 301 nm was taken as the excitation wavelength. The Stern–Volmer plots as function of NB (*e*), DNT (*f*), TNT (*g*), and PA (*h*) concentration in CH_2_Cl_2_, with an excitation wavelength of 301 nm for **P3** solution; Figure S52: Fluorescence quenching studies of **P3*** (1.0 × 10^−5^ mol/L) recorded in the presence of various amounts of NB (*a*), DNT (*b*), TNT (*c*), and PA (*d*), of which 301 nm was taken as the excitation wavelength. The Stern–Volmer plots as function of NB (*e*), DNT (*f*), TNT (*g*), and PA (*h*) concentration in CH_2_Cl_2_, with an excitation wavelength of 301 nm for **P3*** solution; Figure S53: Fluorescence quenching studies of P4 (1.0 × 10^−5^ mol/L) recorded in the presence of various amounts of NB (*a*), DNT (*b*), TNT (*c*), and PA (*d*), of which 345 nm was taken as the excitation wavelength. The Stern–Volmer plots as function of NB (*e*), DNT (*f*), TNT (*g*), and PA (*h*) concentration in CH_2_Cl_2_, with an excitation wavelength of 345 nm for **P4** solution; Figure S54: Fluorescence quenching studies of **P5** (1.0 × 10^−5^ mol/L) recorded in the presence of various amounts of NB (*a*), DNT (*b*), TNT (*c*), and PA (*d*), of which 325 nm was taken as the excitation wavelength. The Stern–Volmer plots as function of NB (*e*), DNT (*f*), TNT (*g*), and PA (*h*) concentration in CH_2_Cl_2_, with an excitation wavelength of 325 nm for **P5** solution; Table S4: Detailed data of the fluorescence lifetime measurements of **P1-P5** in DCM: τ – lifetime, f - fractional contribution, τ_avg_ – average lifetime, χ^2^ - chi-squared distribution; Figure S55: Time-resolved fluorescence lifetime decay profile of **P5** with DNT (1×10^−4^ M) (*blue*), IRF (*green*). λ_ex_ = 300 nm, λ_ex_ = 392 nm; Figure S56: Time-resolved fluorescence lifetime decay profile of **P5** with DNT (2×10^-4^ M) (*blue*), IRF (*green*). λ_ex_ = 300 nm, λ_ex_ = 392 nm; Figure S57: Time-resolved fluorescence lifetime decay profile of **P5** with DNT (3×10^−4^ M) (*blue*), IRF (*green*). λ_ex_ = 300 nm, λ_ex_ = 392 nm; Figure S58: Time-resolved fluorescence lifetime decay profile of **P5** with DNT (4×10^−4^ M) (*blue*), IRF (*green*). λ_ex_ = 300 nm, λ_ex_ = 392 nm; Figure S59: Photos of sensor materials **M1–M3** obtained by electrospinning of fluorescent polymers onto melamine-formaldehyde substrate. For shots under UV illumination, the source of illumination was fixed relative to the material; Figure S60: Photos of the typical sensor cartridge loadout used for measurements under UV illumination (365 nm) shot by the phone camera (*left*) and the camera of the fluorescence recorder (*right*); Figure S61: The optical scheme of fluorescence recorder. The device has disassembled for the sensor installation; Figure S62: Fluorescence responses of sensor materials to NB and DNT vapors at a concentration of 10% from saturated one in a 100 sec exposure interval. The 3σ noise levels of registered fluorescence intensity difference are marked by horizontal dashed lines; Table S5: Detection times of obtained sensor materials towards nitroaromatic vapours when applied in the fluorescence recorder. Saturated vapor concentrations at 25 °C; Figure S63: The scheme of vapor measurements setup; Figure S64: Vapor vessels, the 3 L glass vessel on the left, and the 160 mL syringe vessel installed onto the syringe driver on the right. References [44,45,46,47] are cited in the Supplementary Materials.

## Sample Availability

Samples of the compounds **P1–P5** are available from the authors.
