# Supplementary material for: Random Copolymers of Styrene with Pendant Fluorophore Moieties: Synthesis and Applications as Fluorescence Sensors for Nitroaromatics
Source: Molecules. 2022 Oct 17;27(20):6957. doi: 10.3390/molecules27206957 (PMC9612241; doi:10.3390/molecules27206957)
Supplement: Supplementary file 1 [file molecules-27-06957-s001.zip › molecules-1969205-supplementary.pdf]

Supplementary Material

Random copolymers of styrene with pendant fluorophore moieties: Synthesis and applications as fluorescence sensors for nitroaromatics

Mohamad Zen Eddin[a,b,c], **Ekaterina F. Zhilina**[a], **Roman D. Chuvashov**[b], **Alyona I. Dubovik**[b], **Alexandr V. Mekhaev**[a], **Konstantin A. Chistyakov**[a], **Anna A. Baranova**[b], **Konstantin O. Khokhlov**[b], **Gennady L. Rusinov**[a,b], **Egor V. Verbitskiy**[a,b,*], **Valery N. Charushin**[a,b]

[a]*I. Postovsky Institute of Organic Synthesis, Ural Branch of the Russian Academy of Sciences, S. Kovalevskaya Str., 22, Ekaterinburg, 620108, Russia*

[b]*Ural Federal University, Mira St. 19, Ekaterinburg, 620002, Russia*

[c]*University of Aleppo, Mouhafaza str., Aleppo, Syria*

Table of contents

General Information. 6

Figure S1. ^{1}H NMR (500 MHz, $CDCl_3$) spectrum of **P1** 10

Figure S2. ^{1}H NMR (500 MHz, $CDCl_3$) spectrum of **P2** 11

Figure S3. ^{1}H NMR (500 MHz, $CDCl_3$) spectrum of **P3** 11

Figure S4. ^{1}H NMR (500 MHz, $CDCl_3$) spectrum of **P4** 12

Figure S5. ^{1}H NMR (500 MHz, $CDCl_3$) spectrum of **P5** 12

Figure S6. IR spectrum of **I**. 13

Figure S7. IR spectrum of **II**. 13

Figure S8. IR spectrum of **III**. 14

Figure S9. IR spectrum of **IV**. 14

Figure S10. IR spectrum of **V**. 15

Figure S11. IR spectrum of **P1**. 15

Figure S12. IR spectrum of **P2**. 16

Figure S13. IR spectrum of **P3**. 16

Figure S14. IR spectrum of **P3***. 17

Figure S15. IR spectrum of **P4**. 17

Figure S16. IR spectrum of **P5**. 18

Table S1. IR characteristic frequencies of **I-V** and **P1-P5** 19

Figure S17. Absorption (*blue*), fluorescence emission (*green*) and fluorescence excitation (*blue dashed*) spectra of compound **P1** in CH_2Cl_2. 20

Figure S18. Absorption (*blue*), fluorescence emission (*green*) and fluorescence excitation (*blue dashed*) spectra of compound **P2** in CH_2Cl_2. 20

Figure S19. Absorption (*blue*), fluorescence emission (*green*) and fluorescence excitation (*blue dashed*) spectra of compound **P3*** in CH_2Cl_2. 21
Figure S20. Absorption (*blue*), fluorescence emission (*green*) and fluorescence excitation (*blue dashed*) spectra of compound **P3** in CH_2Cl_2. 21
Figure S21. Absorption (*blue*), fluorescence emission (*green*) and fluorescence excitation (*blue dashed*) spectra of compound **P5** in CH_2Cl_2. 22
Figure S22. Excitation (*green*) and emission (*blue*) spectra of solid powder **P1**. 22
Figure S23. Excitation (*green*) and emission (*blue*) spectra of solid powder **P2**. 23
Figure S24. Excitation (*green*) and emission (*blue*) spectra of solid powder **P3**. 23
Figure S25. Excitation (*blue*) and emission (*green*) spectra of solid powder **P3***. 24
Figure S26. Excitation (*green*) and emission (*blue*) spectra of solid powder **P4**. 24
Figure S27. Excitation (*green*) and emission (*blue*) spectra of solid powder **P5.** 25
Table S2. Detailed data of the fluorescence lifetime measurements of **P1-P5** in DCM: τ – lifetime, *f* - fractional contribution, τ_{avg} – average lifetime, χ^2 - chi-squared distribution. 25
Figure S28. Time-resolved fluorescence lifetime decay profile of **P1** (*green*), instrumental response function (IRF, *blue*). λ_{ex} = 300 nm, λ_{ex} = 362 nm. 26
Figure S29. Time-resolved fluorescence lifetime decay profile of **P1** (green)), IRF (*blue*). λ_{ex} = 300 nm, λ_{ex} = 345 nm. 26
Figure S30. Time-resolved fluorescence lifetime decay profile of **P2** (*green*), IRF (*blue*). λ_{ex} = 300 nm, λ_{ex} = 358 nm. 27
Figure S31. Time-resolved fluorescence lifetime decay profile of **P3** (*green*), IRF (*blue*). λ_{ex} = 300 nm, λ_{ex} = 376 nm. 27
Figure S32. Time-resolved fluorescence lifetime decay profile of **P3** (*green*), IRF (*blue*). λ_{ex} = 300 nm, λ_{ex} = 359 nm. 28
Figure S33. Time-resolved fluorescence lifetime decay profile of **P3*** (*green*), IRF (*blue*). λ_{ex} = 300 nm, λ_{ex} = 376 nm. 28
Figure S34. Time-resolved fluorescence lifetime decay profile of **P3*** (*green*), IRF (*blue*). λ_{ex} = 300 nm, λ_{ex} = 359 nm. 29
Figure S35. Time-resolved fluorescence lifetime decay profile of **P4** (*green*), IRF (*blue*). λ_{ex} = 300 nm, λ_{ex} = 478 nm. 29
Figure S36. Time-resolved fluorescence lifetime decay profile of **P4** (*green*), IRF (*blue*). λ_{ex} = 300 nm, λ_{ex} = 401 nm. 30
Figure S37. Time-resolved fluorescence lifetime decay profile of **P4** (*green*), IRF (*blue*). λ_{ex} = 300 nm, λ_{ex} = 383 nm. 30

Figure S38. Time-resolved fluorescence lifetime decay profile of **P5** (*green*), IRF (*blue*). λ_{ex} = 300 nm, λ_{ex} = 392 nm. 30
Table S3. Detailed data of the fluorescence lifetime measurements of solid powders **P1-P5**: τ – lifetime, *f* - fractional contribution, τ_{avg} – average lifetime, χ^2 - chi-squared distribution....31
Figure S39. Time-resolved fluorescence lifetime decay profile of solid **P1** (*green*), instrumental response function (IRF, *blue*). λ_{ex} = 300 nm, λ_{ex} = 362 nm....31
Figure S40. Time-resolved fluorescence lifetime decay profile of solid **P1** (*green*), instrumental response function (IRF, *blue*). λ_{ex} = 300 nm, λ_{ex} = 355 nm....32
Figure S41. Time-resolved fluorescence lifetime decay profile of solid **P1** (*green*), instrumental response function (IRF, *blue*). λ_{ex} = 300 nm, λ_{ex} = 346 nm....32
Figure S42. Time-resolved fluorescence lifetime decay profile of solid **P2** (*green*), instrumental response function (IRF, *blue*). λ_{ex} = 300 nm, λ_{ex} = 359 nm....32
Figure S43. Time-resolved fluorescence lifetime decay profile of solid **P3** (*green*), instrumental response function (IRF, *blue*). λ_{ex} = 300 nm, λ_{ex} = 377 nm....33
Figure S44. Time-resolved fluorescence lifetime decay profile of solid **P3** (*green*), instrumental response function (IRF, *blue*). λ_{ex} = 300 nm, λ_{ex} = 364 nm....33
Figure S45. Time-resolved fluorescence lifetime decay profile of solid **P3*** (*green*), instrumental response function (IRF, *blue*). λ_{ex} = 300 nm, λ_{ex} = 377 nm....33
Figure S46. Time-resolved fluorescence lifetime decay profile of solid **P3*** (*green*), instrumental response function (IRF, *blue*). λ_{ex} = 300 nm, λ_{ex} = 364 nm....34
Figure S47. Time-resolved fluorescence lifetime decay profile of solid **P4** (*green*), instrumental response function (IRF, *blue*). λ_{ex} = 300 nm, λ_{ex} = 457 nm....34
Figure S48. Time-resolved fluorescence lifetime decay profile of solid **P5** (*green*), instrumental response function (IRF, *blue*). λ_{ex} = 300 nm, λ_{ex} = 420 nm....34
Figure S49. Fluorescence quenching studies of **P1** (1.0×10^{-5} mol/L) recorded in the presence of various amounts of NB (*a*), DNT (*b*), TNT (*c*), and PA (*d*), of which 290 nm was taken as the excitation wavelength. The Stern–Volmer plots as function of NB (*e*), DNT (*f*), TNT (*g*), and PA (*h*) concentration in CH_2Cl_2, with an excitation wavelength of 290 nm for **P1** solution....35
Figure S50. Fluorescence quenching studies of **P2** (1.0×10^{-5} mol/L) recorded in the presence of various amounts of NB (*a*), DNT (*b*), TNT (*c*), and PA (*d*), of which 294 nm was taken as the excitation wavelength. The Stern–Volmer plots as function of NB (*e*), DNT (*f*), TNT (*g*), and PA (*h*) concentration in CH_2Cl_2, with an excitation wavelength of 294 nm for **P2** solution....36
Figure S51. Fluorescence quenching studies of **P3** (1.0×10^{-5} mol/L) recorded in the presence of various amounts of NB (*a*), DNT (*b*), TNT (*c*), and PA (*d*), of which 301 nm was taken as the excitation

wavelength. The Stern–Volmer plots as function of NB (*e*), DNT (*f*), TNT (*g*), and PA (*h*) concentration in CH_2Cl_2, with an excitation wavelength of 301 nm for **P3** solution. 37

Figure S52. Fluorescence quenching studies of **P3*** (1.0 × 10^{-5} mol/L) recorded in the presence of various amounts of NB (*a*), DNT (*b*), TNT (*c*), and PA (*d*), of which 301 nm was taken as the excitation wavelength. The Stern–Volmer plots as function of NB (*e*), DNT (*f*), TNT (*g*), and PA (*h*) concentration in CH_2Cl_2, with an excitation wavelength of 301 nm for **P3*** solution. 38

Figure S53. Fluorescence quenching studies of **P4** (1.0 × 10^{-5} mol/L) recorded in the presence of various amounts of NB (*a*), DNT (*b*), TNT (*c*), and PA (*d*), of which 345 nm was taken as the excitation wavelength. The Stern–Volmer plots as function of NB (*e*), DNT (*f*), TNT (*g*), and PA (*h*) concentration in CH_2Cl_2, with an excitation wavelength of 345 nm for **P4** solution. 39

Figure S54. Fluorescence quenching studies of **P5** (1.0 × 10^{-5} mol/L) recorded in the presence of various amounts of NB (*a*), DNT (*b*), TNT (*c*), and PA (*d*), of which 325 nm was taken as the excitation wavelength. The Stern–Volmer plots as function of NB (*e*), DNT (*f*), TNT (*g*), and PA (*h*) concentration in CH_2Cl_2, with an excitation wavelength of 325 nm for **P5** solution. 40

Table S4. Detailed data of the fluorescence lifetime measurements of **P1-P5** in DCM: τ – lifetime, *f* - fractional contribution, τ_{avg} – average lifetime, χ^2 - chi-squared distribution. 41

Figure S55. Time-resolved fluorescence lifetime decay profile of **P5** with DNT (1x10^{-4}M) (*blue*), IRF (*green*). λ_{ex} = 300 nm, λ_{ex} = 392 nm. 41

Figure S56. Time-resolved fluorescence lifetime decay profile of **P5** with DNT (2x10^{-4}M) (*blue*), IRF (*green*). λ_{ex} = 300 nm, λ_{ex} = 392 nm. 41

Figure S57. Time-resolved fluorescence lifetime decay profile of **P5** with DNT (3x10^{-4}M) (*blue*), IRF (*green*). λ_{ex} = 300 nm, λ_{ex} = 392 nm. 42

Figure S58. Time-resolved fluorescence lifetime decay profile of **P5** with DNT (4x10^{-4}M) (*blue*), IRF (green). λ_{ex} = 300 nm, λ_{ex} = 392 nm. 42

Figure S59. Photos of sensor materials **M1-M3** obtained by electrospinning of fluorescent polymers onto melamine-formaldehyde substrate. For shots under UV illumination, the source of illumination was fixed relative to the material. 43

Figure S60. Photos of the typical sensor cartridge loadout used for measurements under UV illumination (365 nm) shot by the phone camera (*left*) and the camera of the fluorescence recorder (*right*). 43

Figure S61. The optical scheme of fluorescence recorder. The device has disassembled for the sensor installation. 44

Figure S62. Fluorescence responses of sensor materials to NB and DNT vapors at a concentration of 10% from saturated one in a 100 sec exposure interval. The 3*σ* noise levels of registered fluorescence intensity difference are marked by horizontal dashed lines 44

Table S5. Detection times of obtained sensor materials towards nitroaromatic vapours when applied in the fluorescence recorder. Saturated vapor concentrations at 25C 45
Figure S63. The scheme of vapor measurements setup. 46
Figure S64. Vapor vessels, the 3 L glass vessel on the left, and the 160 mL syringe vessel installed onto the syringe driver on the right. 46

General Information.

All reagents and solvents were obtained from commercial sources and used as received. Nitroaromatic explosives, including 2,4-dinitrotoluene (DNT), 2,4,6-trinitrotoluene (TNT), picric acid (PA), and nitrobenzene (NB) were of analytical grade and used directly without further purification. Tetrahydrofuran (THF) was distilled over CaH_2 and prepurified from dissolved oxygen by bubbling argon through solutions for 1 h. (***Caution***: *All nitro-containing compounds used in the present study are high explosives and should be handled only in small analytical quantities*).

^1H NMR spectra were recorded on an AVANCE-500 instruments using Me_4Si as an internal standard.

Molecular weight distribution was determined by GPC on Agilent 1200 device with LSD (Evaporative Light Scattering Detector) detector and column Agilent Resipore 300×7.5 mm-2 piece, mobile phase 100% THF at a flow rate of 1.5 ml/min, the volume of the injected sample 100 μl.

Thermogravimetric analysis (TGA) was performed using TGA/DSC 1 (Mettler-Toledo, Switzerland) at a heating rate of 10 °C/min under an argon atmosphere (60 ml/min). The decomposition temperature (T_d) was determined by extrapolation onset temperature – this is the point of intersection of the starting-mass baseline and the tangent to the TGA curve at the point of the maximum gradient, which has been defined by a peak temperature of the DTG (1st derivative of the TGA curve).

IR spectra of samples (solid powders) were recorded on a Spectrum Two Fourier transform IR spectrometer (PerkinElmer) equipped with the universal attenuated total reflectance accessory (UATR) in the frequency range 4000÷400 cm^{-1}. Spectrum processing and band intensity determination were carried out using the special software (Spectrum) supplied with the spectrometer.

UV/vis spectra were recorded with Shimadzu UV-2600 spectrophotometer. Photoluminescent spectra were recorded on a FS5 Edinburgh Instruments spectrofluorometer. UV/vis and fluorescence spectra of solutions were recorded using standard 1 cm quartz cells at room temperature. Fluorescence spectra of solid powders were recorded using SC-10 module at room temperature. The Φ_F values were calculated using the established procedure with 2-aminopyridine in 0.1N H_2SO_4 (Φ_F = 0.60)[1] Absolute quantum yields have been measured using integrating sphere SC-30.

The emission lifetimes have been measured using TCSPC option of FS5 Edinburgh Instruments spectrofluorometer. The sample has been excited by EPLED-300 picosecond pulsed diode centered at 300 nm. The instrument response function (IRF) has been recorded under described conditions by replacing the sample with a silica diffuser. The time decay data have been analyzed by nonlinear least-squares fitting with deconvolution of the IRF using the Fluoracle software package.

---

[1] K. Rurack, Standardization and Quality Assurance in Fluorescence Measurements I, Springer Berlin Heidelberg, Berlin, Heidelberg, 2008, pp. 101–145

The fluorescence quenching studies were carried out on a Varian Cary Eclipse fluorescence spectrophotometer at room temperature in dichloromethane. For each analyte, the typical test procedure was as follows: the series of solution with 1×10^{-6} M of polymer and different concentration of analyte (1×10^{-5} – 5×10^{-4} M) were prepared. The solutions were incubated at 298 K for 10 min and then the emission spectra were recorded. The fluorescence spectrum of pure polymer without analyte was first recorded. The plots of I_0/I values of the quenching systems as functions of quencher concentrations ([Q]) were well described by the Stern–Volmer equation, $I_0/I=1+K\text{sv}[Q]$, where I_0 and I are fluorescence intensities without and in the presence of analyte.

Analyte concentration in the airflow is meant as its vapor pressure. To correct concentration values based on gas temperature during measurement, the Antoine equation was used for NB[2], and the Clausius-Clapeyron equation for 2,4-DNT, 2,4,6-TNT, and PA[3]. The coefficient A in the latter was adjusted per analyte to make the resulting vapor pressures match pressure data from ref.[4] at 25 °C.

Ender-3 3D printer (Creality, China) was used to manufacture ABS sensor cartridges. Images of sensor materials were obtained with optical microscope Mikromed-3 and attached camera ToupTek UCMOS05100KPA with the use of 365nm 5mW UV diode illumination. The flow rates were measured via a rotameter. The BME280 (Bosch, Germany) sensor was used to measure ambient humidity and temperature during the preparation of electrospun materials. Custom-made electrospinning apparatus was used to obtain sensor materials based on synthesized polymers.

Preparation of electrospun sensing materials. Prepared polymers were dissolved in THF to obtain a 5% w/v solution. The electrospinning was performed at a voltage of 15 kV with a collecting distance of 15cm. The permeable deposition substrate was obtained by cutting a melamine-formaldehyde foam into 2 mm thick sheets. The ground electrode collector was covered with 2 layers of scotch tape, and the substrate sheet was fixed on it. The syringe with 400–450 μL of the polymer solution was mounted on a custom-made syringe pump. The solution was electrospun in a closed box through a needle with a 0.7 mm internal diameter at a constant volume rate of 1.2 mL h^{-1} at ambient 25% relative humidity (RH) and 22 °C. The humidity and temperature data were registered inside the electrospinning box before deposition. Prepared films were stored in closed plastic containers with activated charcoal.

Sensor element. To employ obtained permeable sensor materials, the sensor element cartridge was manufactured via 3D printing from ABS plastic. The cartridge features of 9 radially-positioned through-hole slots for substrates carrying fluorescent materials. The cartridge meant to be installed perpendicular to

---

[2] E. J. Lynch, C.R. Wilke. Vapor Pressure of Nitrobenzene at Low Temperatures. *J. Chem. Eng. Data* **1960**, *5*, 300. https://doi.org/10.1021/je60007a018

[3] H. Östmark, S. Wallin, H.G. Ang. Vapor Pressure of Explosives: A Critical Review. *Propellants Explos. Pyrotech*. **2012**, *37*, 12–23. https://doi.org/10.1002/prep.201100083

[4] R.G. Ewing, M.J. Waltman, D.A. Atkinson, J.W. Grate, P.J. Hotchkiss. The vapor pressures of explosives. *Trends Anal. Chem*. **2013**, *42*, 35–48. https://doi.org/10.1016/j.trac.2012.09.010

the airway to guide air through substrates. To use electrospun fluorescent materials foam sheets were cut into 4×4 mm square fragments and installed into slots. Unused slots were filled with a clean substrate to equalize air resistance. The sensor element also features one impermeable slot in the middle filled with clean foam to account for the substrate's fluorescence intensity.

Fluorescence recorder. To evaluate the sensing performances of obtained materials toward gas-phase analytes, a luminescence recorder was constructed based on previously developed models of fluorescence sniffers.[5] The device uses a compact camera for the parallel recording of luminescence intensities of sensor materials in the visible band. The sensor cartridge carrying a combination of sensor materials is installed into the device at a fixed position relative to the camera, and the air is driven through it via the pump. The video stream processing and measurement automation are controlled from the PC via custom software. The overview of assembled fluorescence recorder is shown in Figure 10.

The fluorescence of sensor materials on the cartridge is excited by a 365 nm LED. A band-pass filter ($\lambda_{0.5}$ = 425 nm, T < 0.1% at 350-409 nm) is used to suppress camera illumination by excitation light. The device uses an OV2640 CMOS camera (OmniVision, China) that performs video capture at 20 frames per second in 3 color channels (440-540 nm, 500-580 nm, and 580-660 nm), allowing to monitor integral fluorescence intensity in time. The optical scheme of the device is shown in Figure S61. The video stream of the sensor cartridge is transmitted to the PC. The fluorescent material's spot on the image is automatically outlined by a contour prior to the measurement, and the brightness of pixels inside the contour is taken as a measure of the fluorescence intensity of the material (Figure S60). Capture parameters such as exposure, gain, and color channel were optimized to give the maximum brightness of the image without pixel overexposure. Processing on PC also includes digital noise filtering, allowing to reach a signal-to-noise ratio of 330 for material at 100 a.u. brightness. The UV illumination power hitting each foam fragment was in the 23 to 29 mW range depending on the position on the cartridge, and the mean losses of intensity due to photodecay in 100 sec interval were 0.76 %, 0.53 %, 0.74 % for materials **M1**, **M2**, and **M3**, respectively.

The air path of the device is made of brass fitting parts and is easy to disassemble for sensor cartridge change and maintenance. The vacuum pump drives the gas through the airway at volume rates up to 1.8 L/min, though the typical volume rate was modulated to 790 mL/min. The sensor element is installed into the inner volume via the sealing adapter 3D-printed from thermopolyurethane. The internal volume in front of the sensor cartridge is about 22 mL, with 790 mL/min flow making for a dwell time of

[5] (a) Chuvashov, R., Baranova, A., Khokhlov, K., Verbitskiy, E. A detection system with low sampling distortion for application in optical array sensing in gas phase (Conference Paper). Proceedings - 2020 7th International Congress on Energy Fluxes and Radiation Effects, EFRE 2020, 9242148, Pages 984-988. https://doi.org/10.1109/EFRE47760.2020.9242148; (b) 3. A.A. Baranova, K.O. Khokhlov, R.D. Chuvashov, E.V. Verbitskiy, E.M. Cheprakova, G.L. Rusinov, V.N. Charushin. The portable detector of nitro-explosives in vapor phase with new sensing elements on the base of pyrimidine scaffolds. *Journal of Physics: Conference Series*. **2017**, *830*, 012159 (6 pages).

1.7 s and lower concentration distortion of sampled vapors. The BME280 sensor positioned inside the airway provides data on humidity, temperature, and pressure inside the sensor chamber. Connections of parts were sealed with polytetrafluoroethylene tape where necessary to prevent air leaks.

Two microcontrollers are used to run the camera, set powers of elements, and exchange sensor data with the PC. The code on the PC provides communication with microcontrollers via wireless network, video stream capture, measurement automatization, and post-measurement data processing. The necessary software is written in Python and MicroPython. The software implements automated measurement by scenario routine which was used to record fluorescent responses to analytes.

Study of fluorescence response to gas-phase analytes. To run gas-phase sensor evaluation, the analyte vapor was introduced to the air stream going into the fluorescence recorder. Sensor cartridges with 3 slots filled per sensor material were prepared. Gas-phase measurements were conducted with the ambient air as a gas mixture with zero concentration of analyte vapor except for measurements with N_2 as the interfering analyte. Necessary valves, connectors, and mixers were 3D printed from ABS. The PVC pipes with 4 mm inner diameter were used to connect elements. The schematic of the testing setup is shown in Figure S63.

A set of vessels based on 160 mL and 20 mL plastic syringes and 3000 mL glass bottles were used for vapor accumulation (Figure S64). The analyte (< 1 g or < 1 mL) was placed inside on the inert substrate and kept for at least 24 h to produce saturated vapors; the same vessels were reused after vapor accumulation. The glass bottle vessels were cleaned with distilled water and air prior to initial use. Syringes contained silicon oil lubricant, which was removed mechanically and with chloroform. Rubber plungers were isolated from the inner volume by aluminum foil, and Teflon tape was applied to fixate the foil and compensate for increased plunger drag. Syringes were ventilated for 2 days after cleaning prior to use. Vessels had two exits at volume ends allowing to create the gas flow through them. The 3000 mL vessel was used only for measurements with non-nitroaromatic interferents; in other cases, 160 mL vessels were used unless stated otherwise. For all measurements, airflow volume speed was set to 790 mL/min.

For measurements with saturated vapors, the airflow source was switched via a valve from the ambient air to the flow through the vessel with saturated vapors. For this case, the concentration during the measurement changes due to dilution; the saturated vapor measurements are annotated with the volume of the used vapor vessel and exposure duration. For the measurement with N_2 as the analyte, the airflow was changed to the line of compressed nitrogen at the same volume rate. Measurements with diluted vapors were performed by dosing out the saturated vapor from the syringe into the airflow to create a constant concentration. The custom-made syringe driver was used to precisely dose vapors from the syringe into the air mixer. For measurements with NB, a needle with 0.7 mm inner diameter was applied to the syringe exit to limit the diffusion-driven leak from the vapor vessel to the airflow. Concentrations of vapors were

corrected based on the ambient temperature registered prior to measurement; all measurements were performed at the ambient pressure from 96 kPa to 98.6 kPa.

The general scenario of the measurement is an interval of sensor exposure to vapor-contaminated air (50 sec or 100 sec) prefaced and followed by intervals of the clean air of the same duration. For the exposure and recovery intervals, the base fluorescence intensity I was predicted by a linear model approximating pre-exposure intensity data. Metrics I_{exp} and I_{recov} were calculated as the mean of differences between I_0 and I for points at the last 2 seconds of the exposure or recovery interval, respectively. Bar plot data in Figures 12 and S62 represent means of metric values for each of 3 fragments per sensor material on the cartridge.

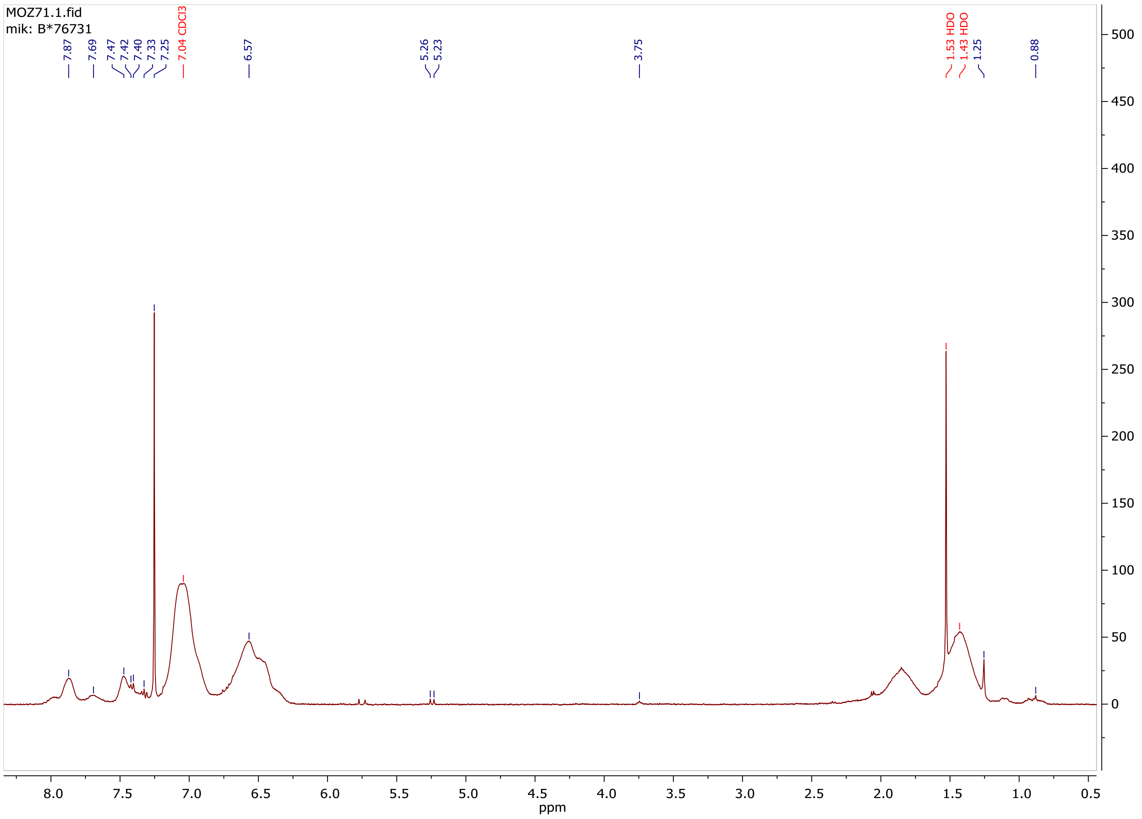

Figure S1. ^{1}H NMR (500 MHz, $CDCl_3$) spectrum of **P1**.

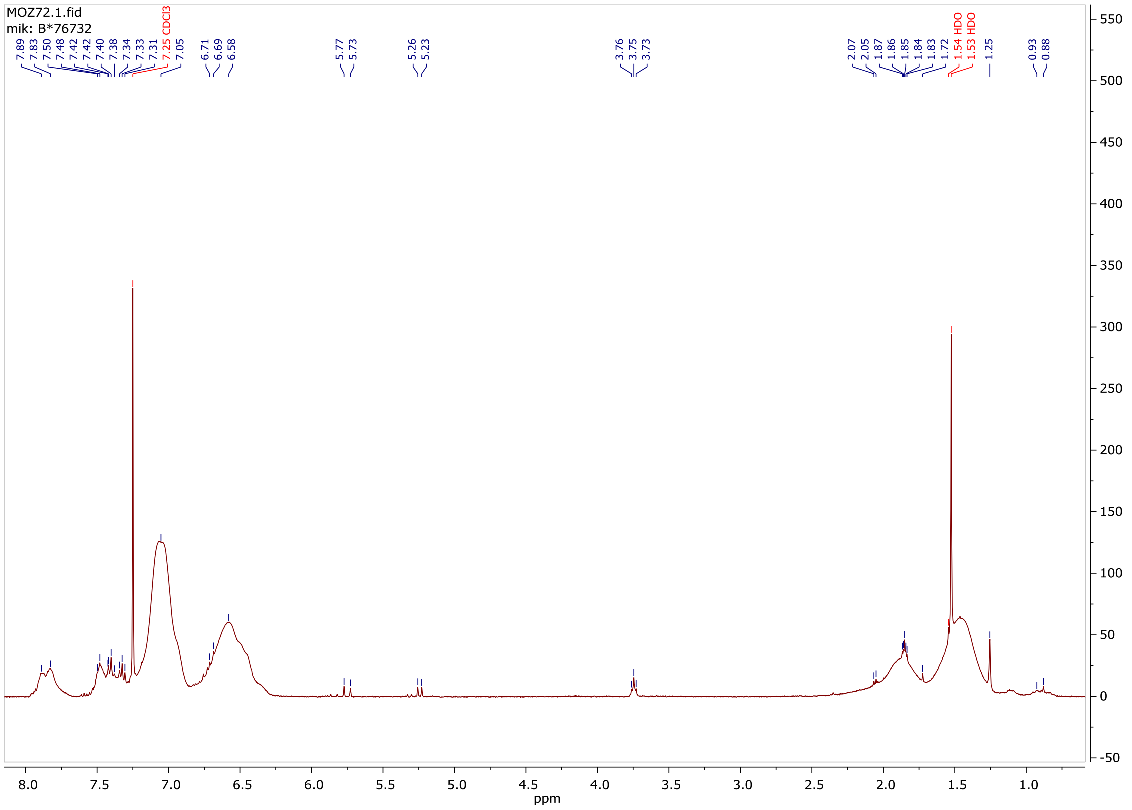

Figure S2. ^{1}H NMR (500 MHz, $CDCl_3$) spectrum of **P2**.

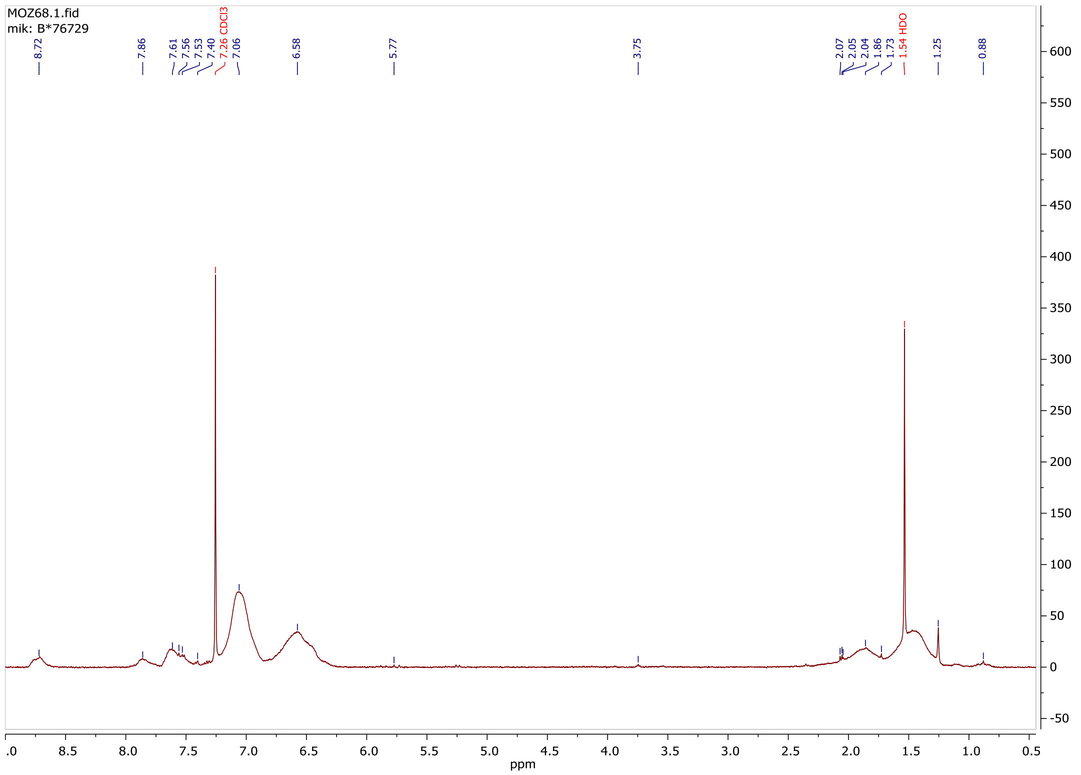

Figure S3. ^{1}H NMR (500 MHz, $CDCl_3$) spectrum of **P3**.

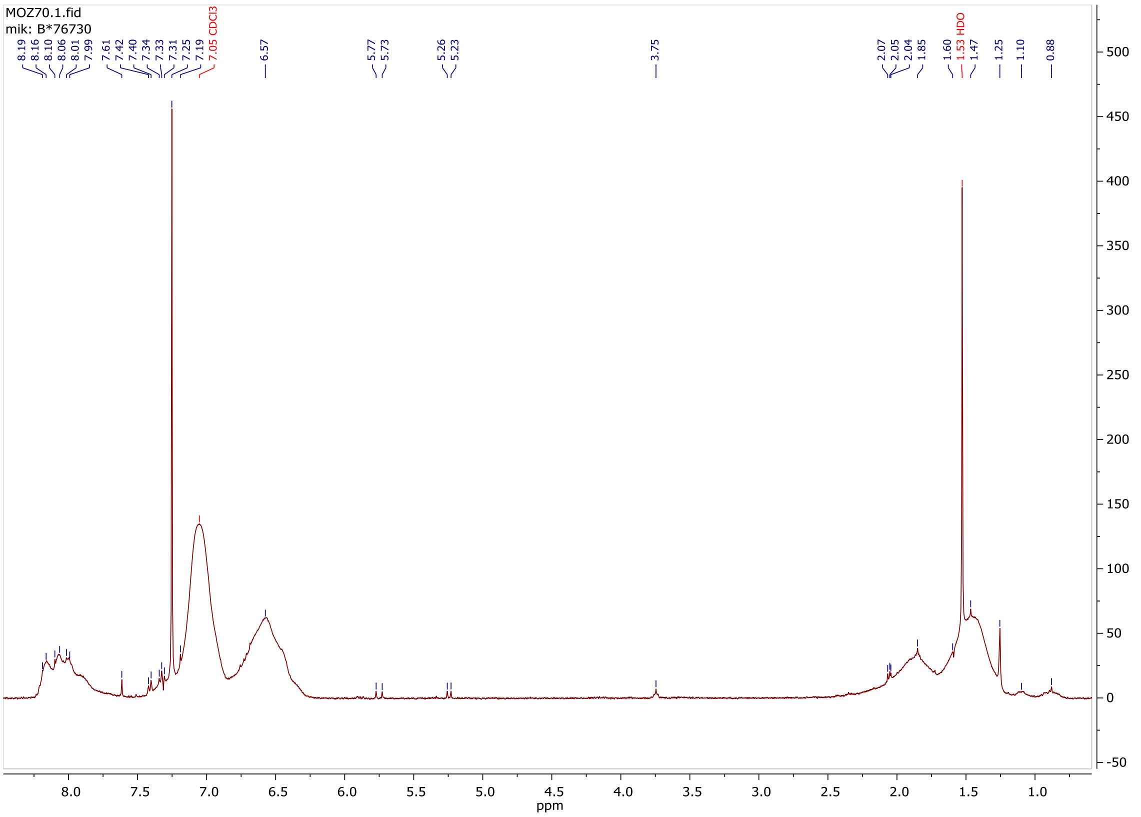

Figure S4. ^{1}H NMR (500 MHz, $CDCl_3$) spectrum of **P4**.

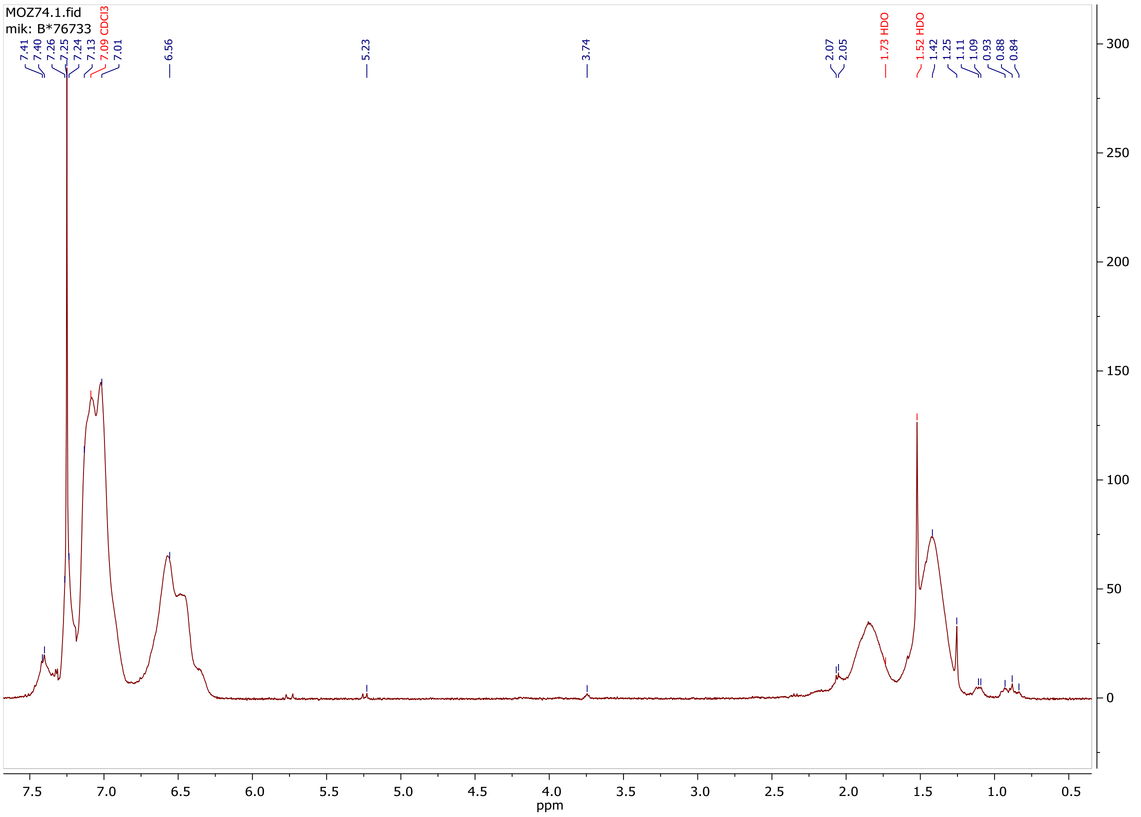

Figure S5. ^{1}H NMR (500 MHz, $CDCl_3$) spectrum of **P5**.

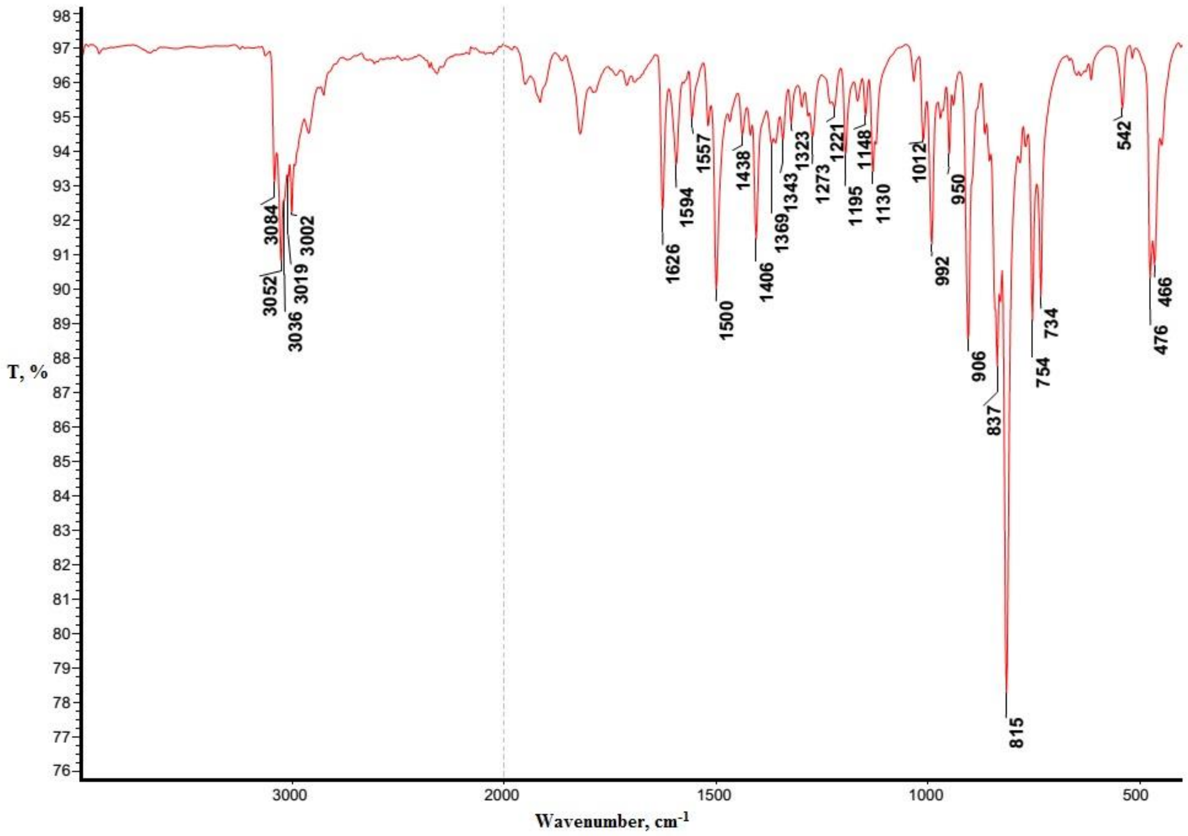

Figure S6. IR spectrum of **I**.

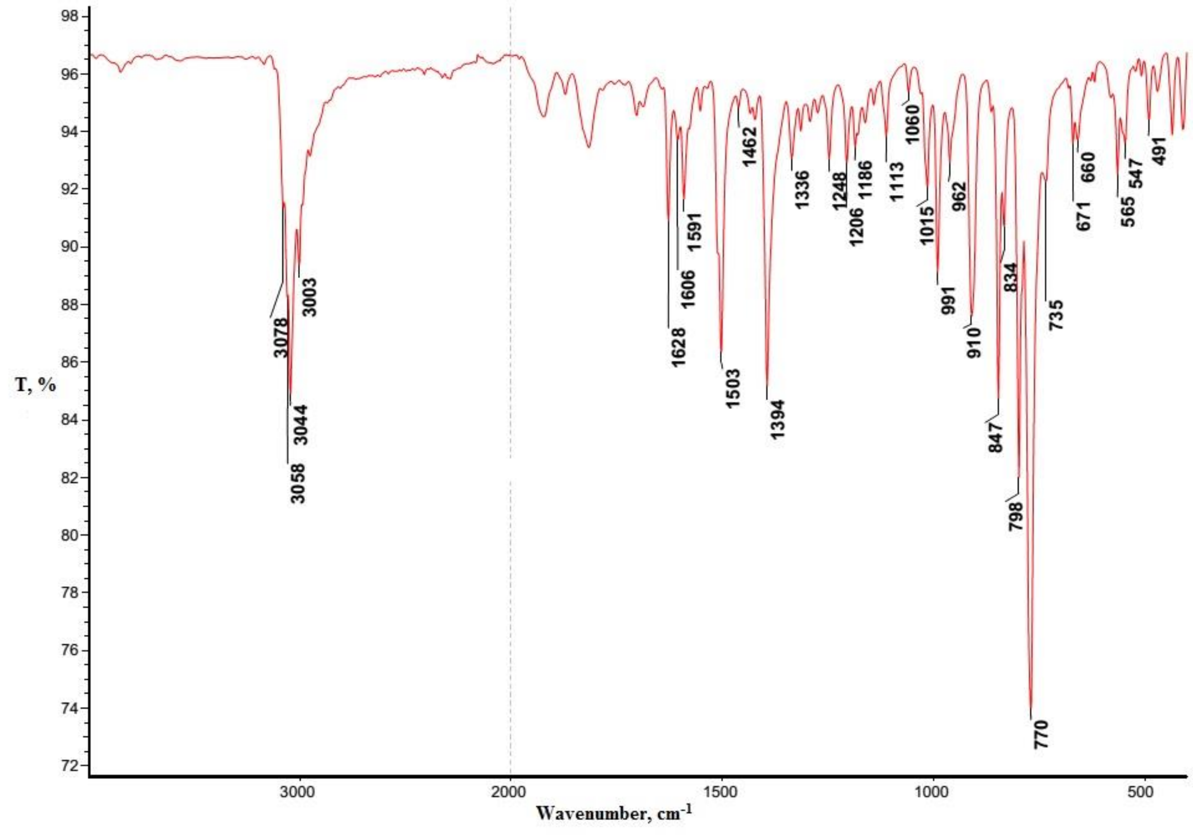

Figure S7. IR spectrum of **II**.

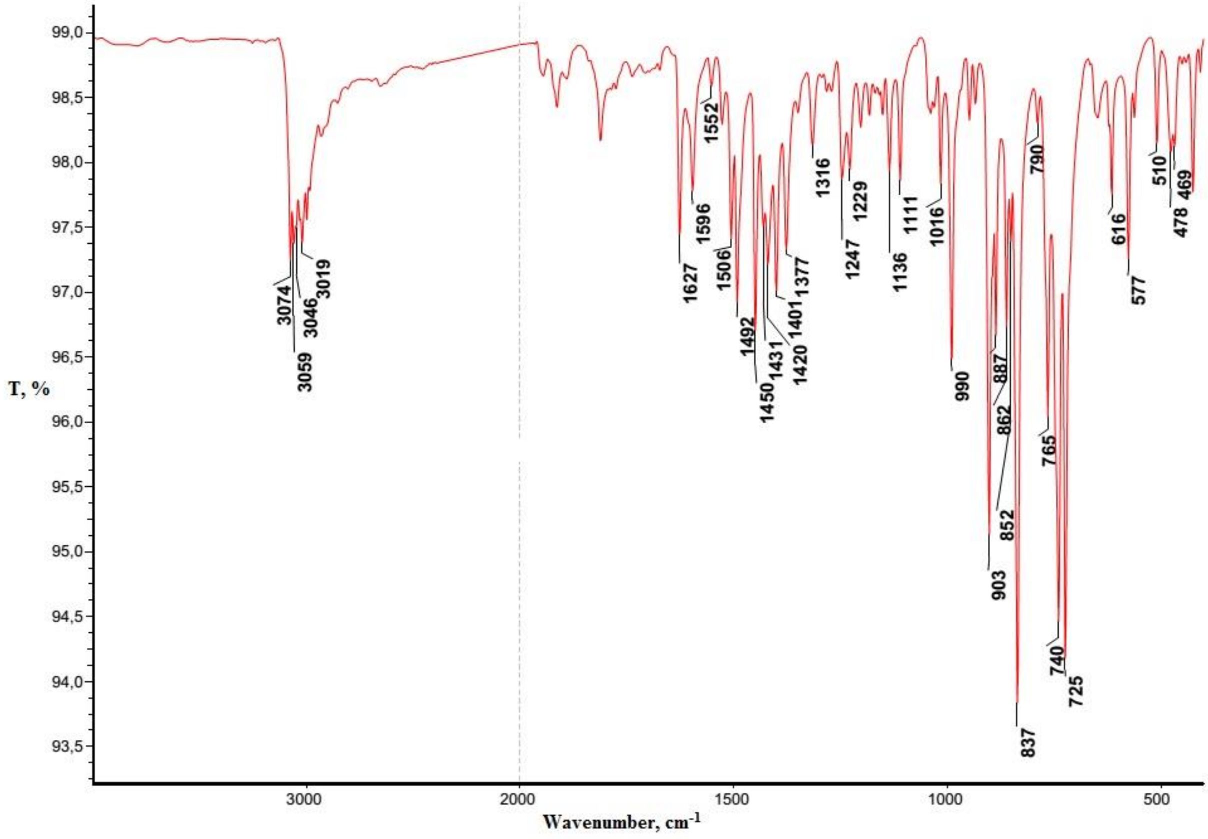

Figure S8. IR spectrum of **III**.

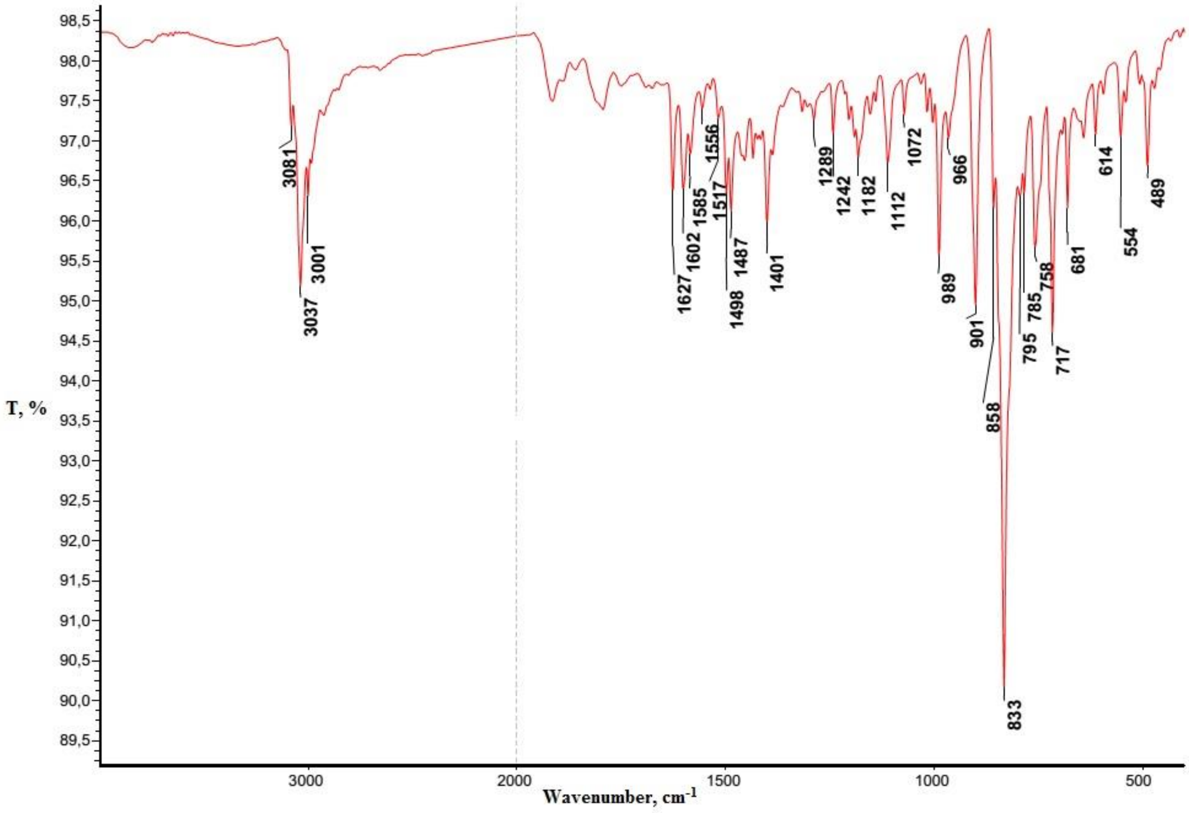

Figure S9. IR spectrum of **IV**.

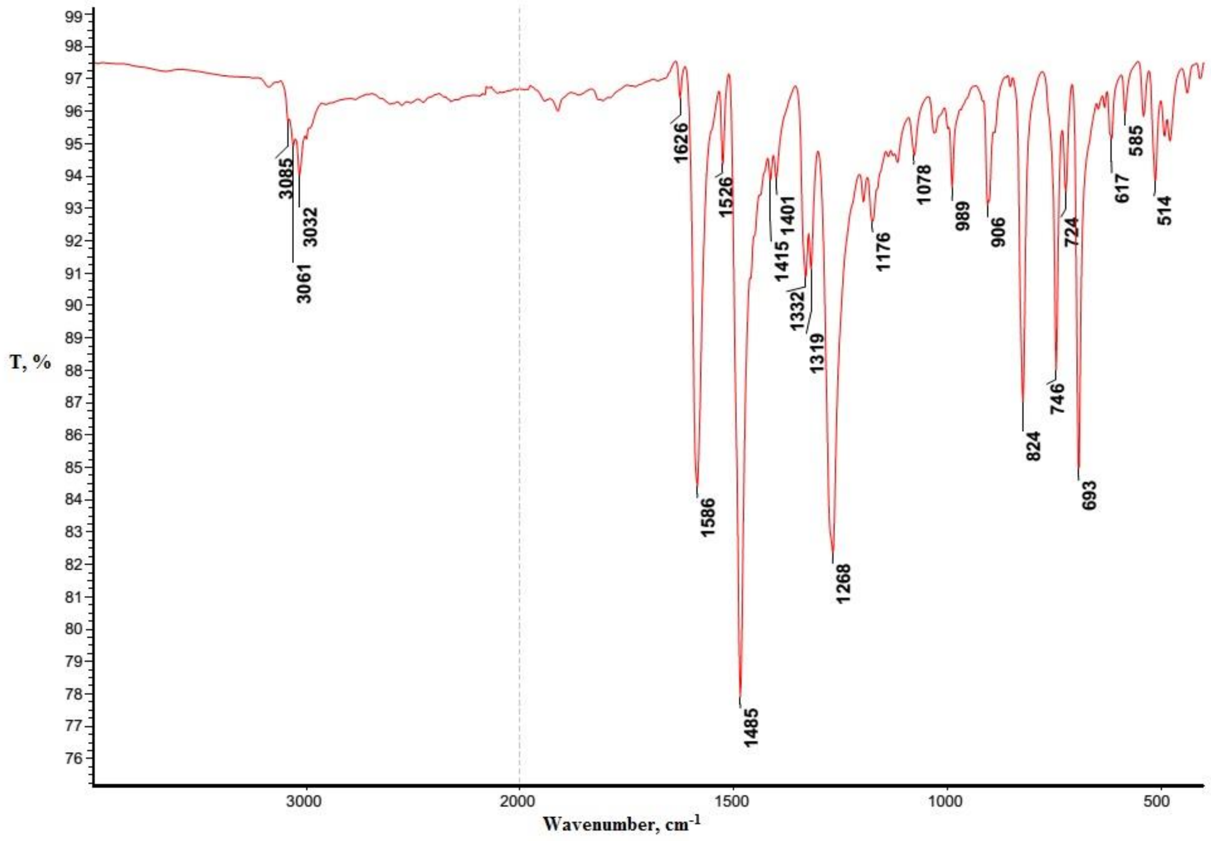

Figure S10. IR spectrum of **V**.

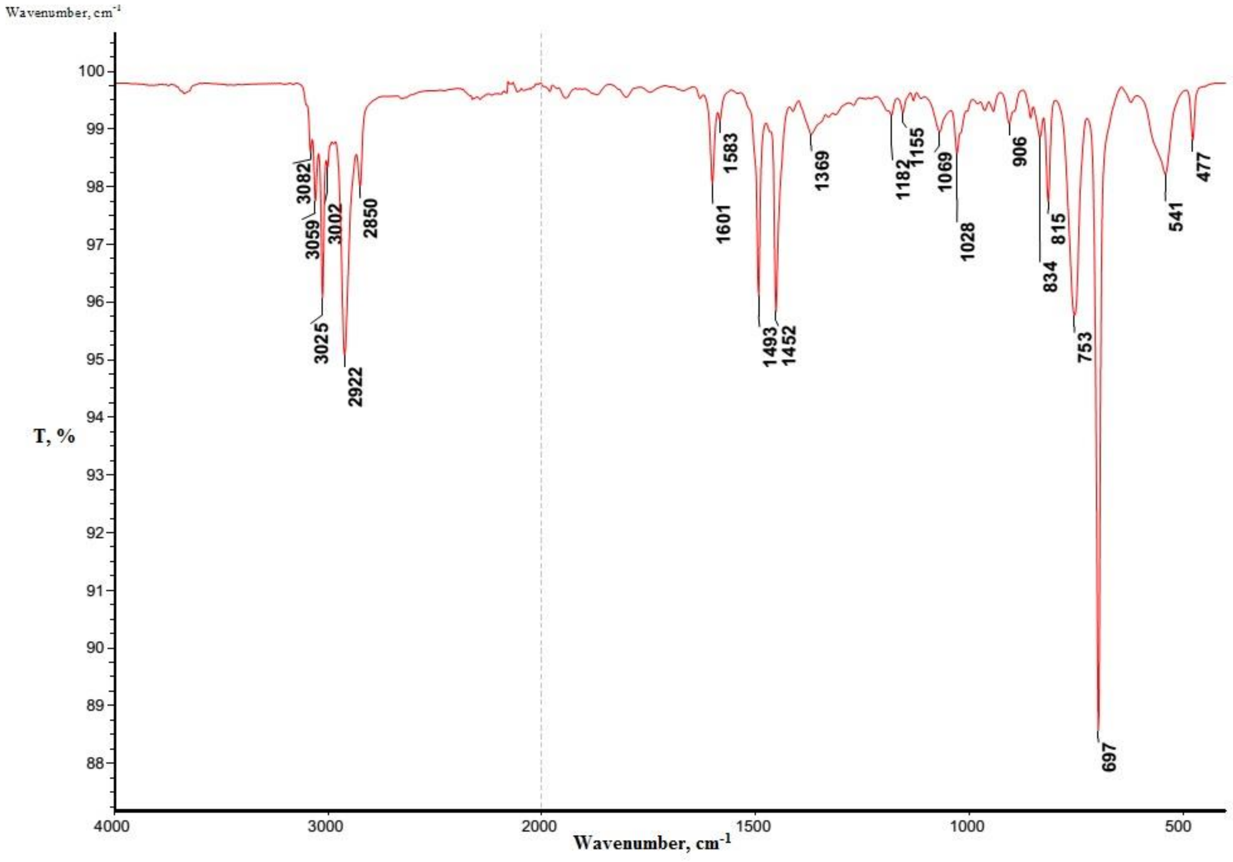

Figure S11. IR spectrum of **P1**.

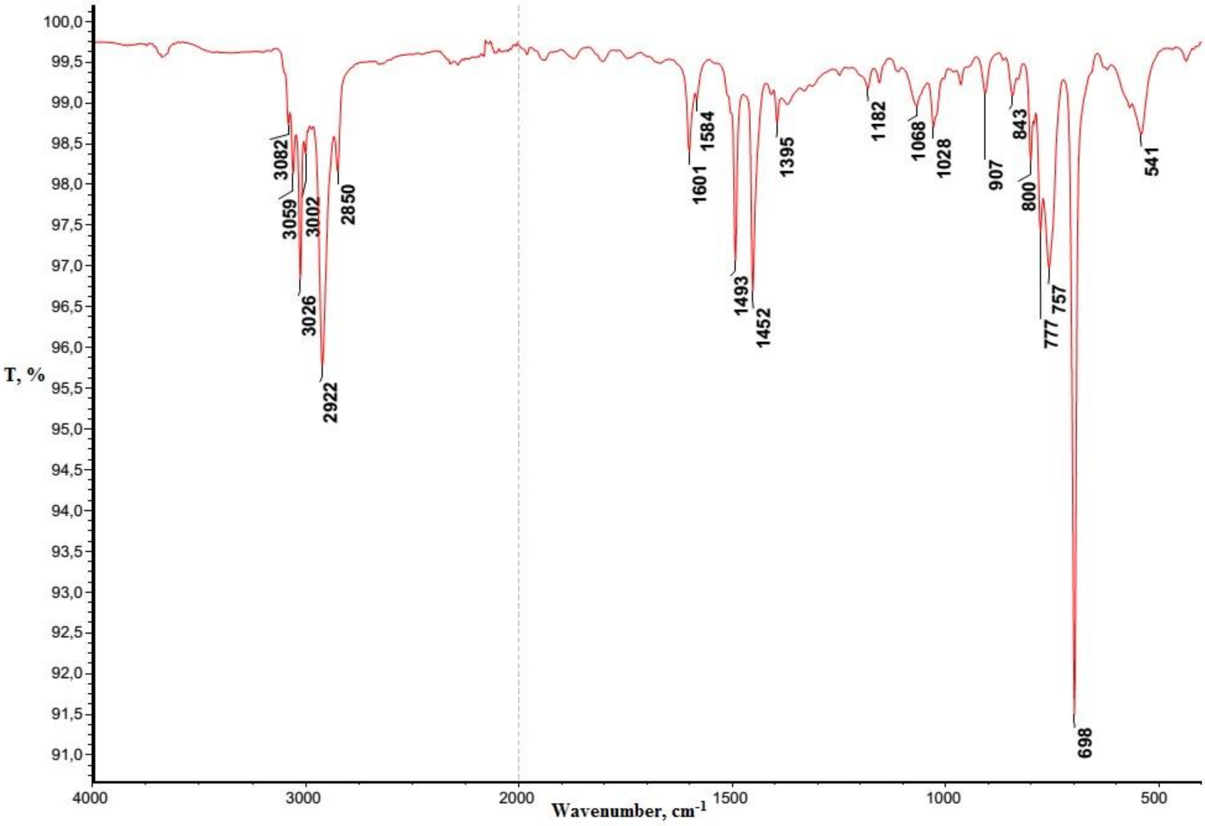

Figure S12. IR spectrum of **P2**.

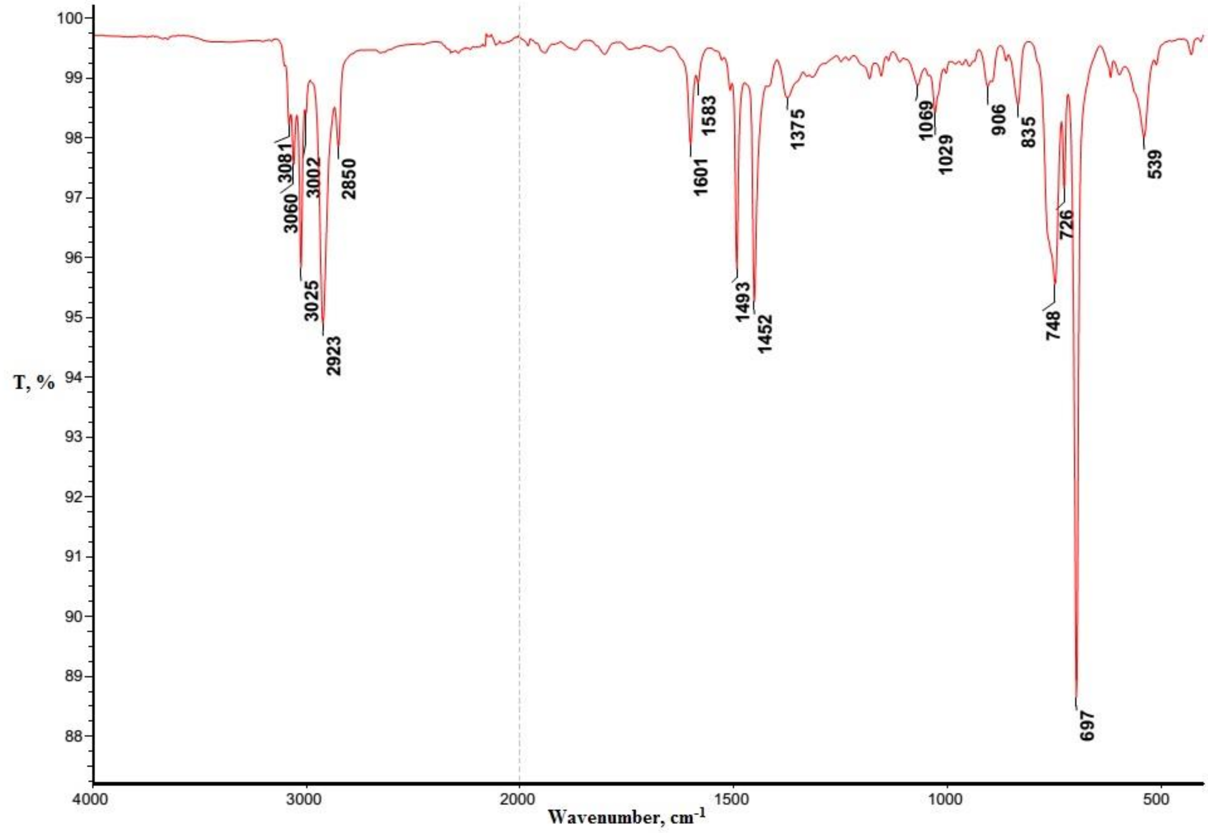

Figure S13. IR spectrum of **P3**.

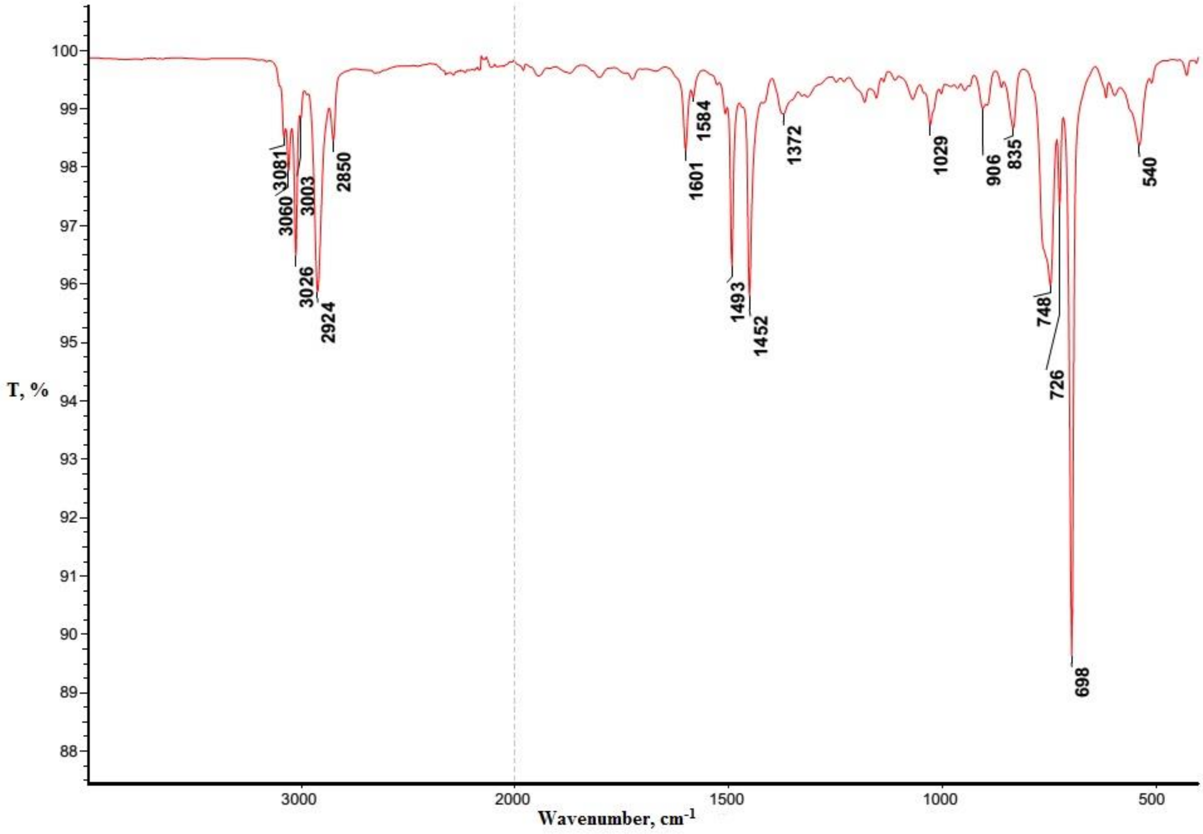

Figure S14. IR spectrum of **P3***.

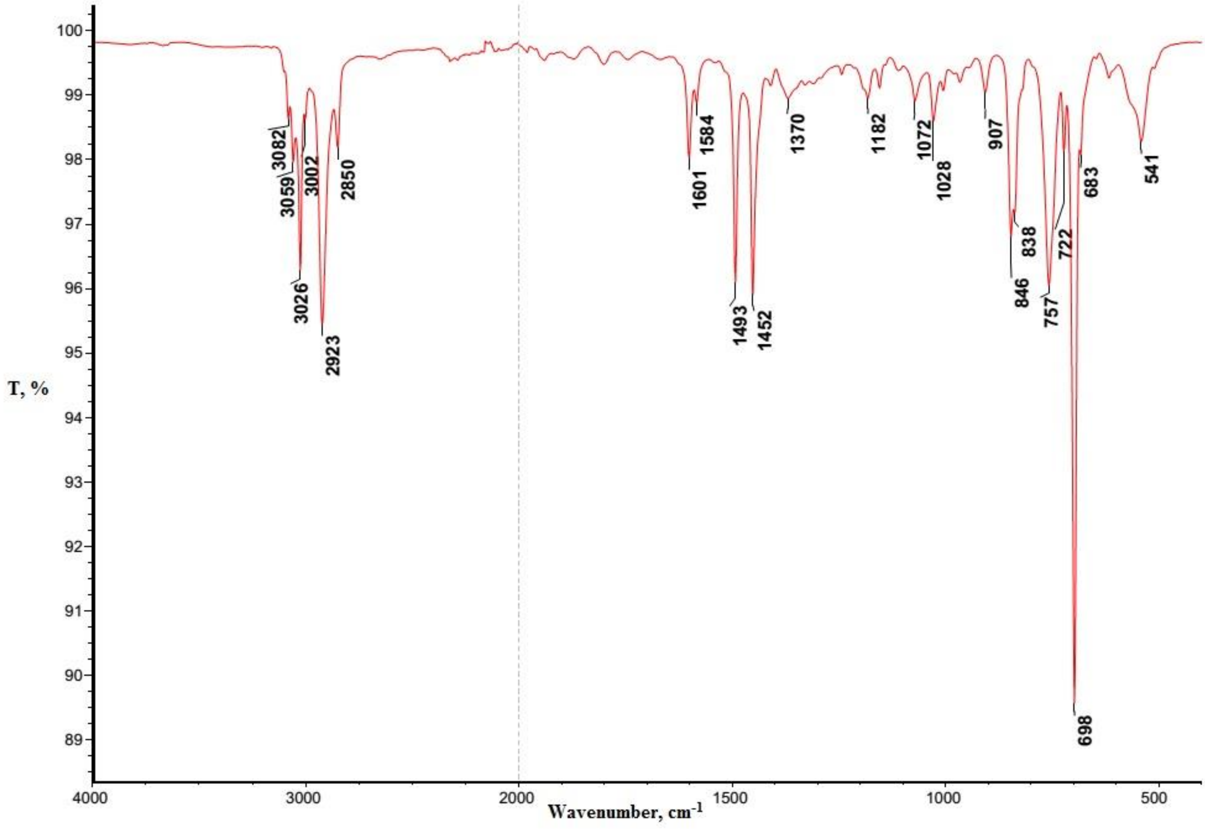

Figure S15. IR spectrum of **P4**.

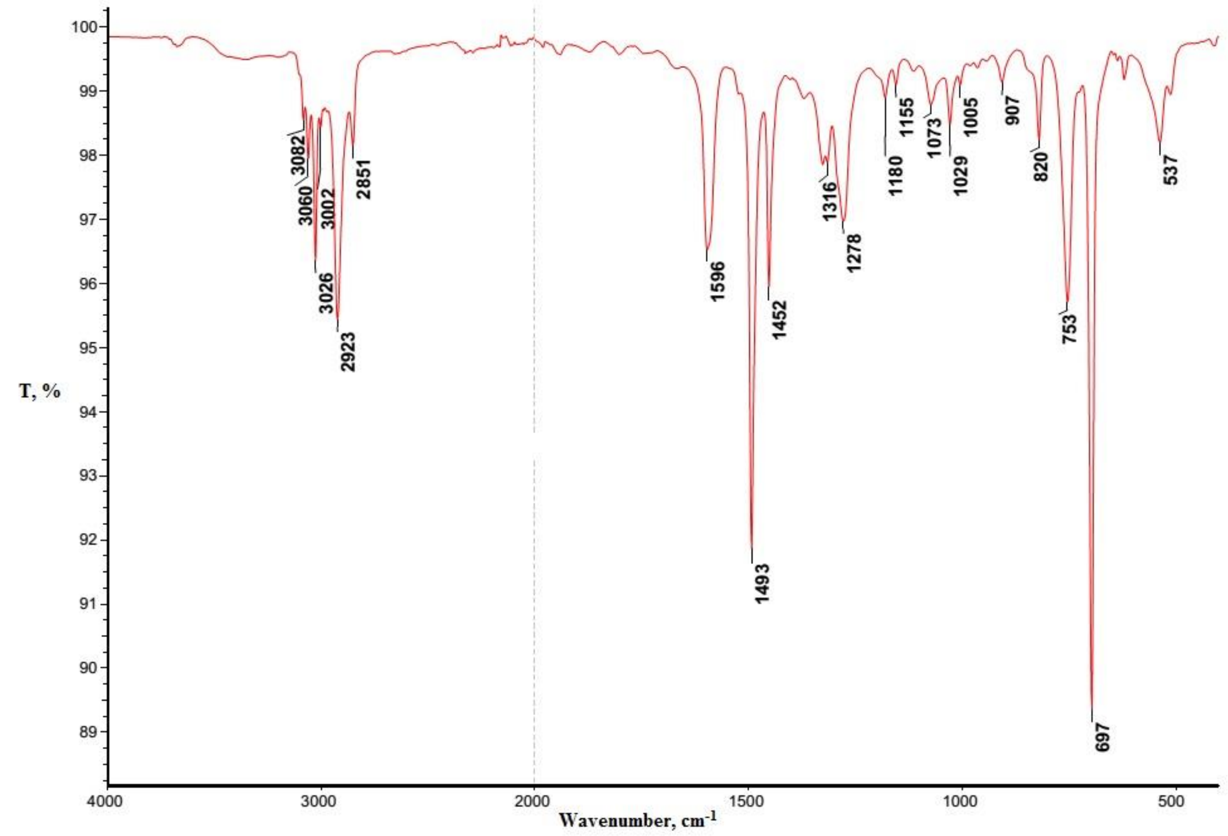

Figure S16. IR spectrum of **P5**.

Table S1. IR characteristic frequencies of **I-V** and **P1-P5**.

**Compound**	**ν (C=C), cm^{-1}**	**ν ($-CH_2-$), cm^{-1} antisymm/symm**	**ν (CH_{arom}, $=CH_2$, =CH-), cm^{-1}**	**ν ($CC_{arom\ ring}$), δ ($-CH_2-$) cm^{-1}**	**ω ($=CH_2$, one lone CH_{arom}), ν_{17B} (B_2) cm^{-1}**	**ω CH_{arom}, cm^{-1}**	
						**two or three adjacent H**	**five or four adjacent H**
**I**	1626	-	3084, 3052, 3036, 3018, 3002	1594, 1557, 1500, 1438	906 s	837, 815	754, 734
**II**	1628	-	3078, 3058, 3044, 3003	1606, 1591, 1503, 1462	910 s	847, 834	798, 770, 735
**III**	1627	-	3074, 3059, 3046, 3019	1596, 1552, 1506, 1492	903 s	887, 862, 837	790, 765, 740, 725
**IV**	1627	-	3081, 3037, 3001	1602, 1585, 1566, 1517, 1498, 1487	901s	858, 833, 795, 785, 758, 717	-
**V**	1626	-	3085, 3061, 3032	1586, 1526, 1485	906 m	824	746, 724, 693
**P1**	-	2922/2850	3082, 3059, 3025, 3002	1601, 1583, 1493, 1452	906 vw	834, 815	753, 697, 541
**P2**	-	2922/2850	3082, 3059, 3026, 3002	1601, 1584, 1493, 1452	907 vw	843, 800, 777	757, 698, 541
**P3**	-	2923/2850	3081, 3060, 3025, 3002	1601, 1583, 1493, 1452	906 vw	835	748, 726, 697, 539
**P3***	-	2924/2850	3081, 3060, 3026, 3003	1601, 1584, 1493, 1452	906 vw	835	748, 726, 698, 540
**P4**	-	2923/2850	3082, 3059, 3026, 3002	1601, 1584, 1493, 1452	907 vw	846, 838	757, 722, 698, 541
**P5**	-	2923/2851	3082, 3060, 3026, 3002	1596, 1493, 1452	907 vw	820	753, 697, 537

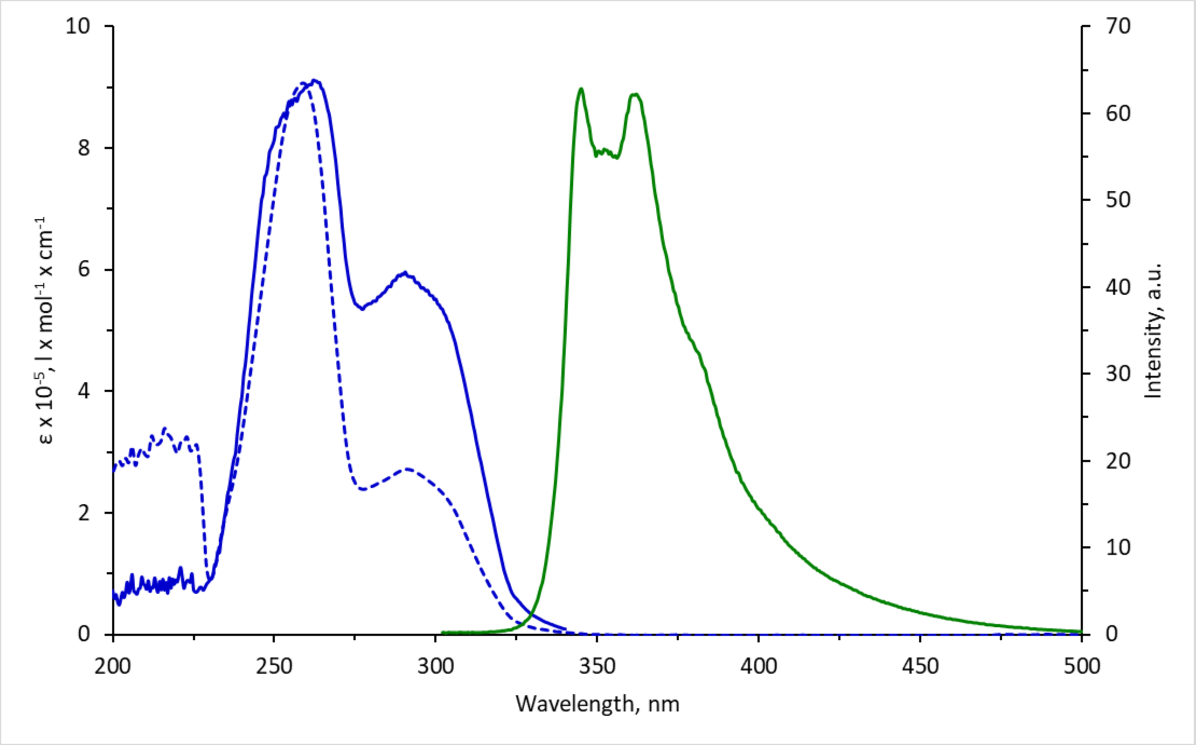

Figure S17. Absorption (*blue*), fluorescence emission (*green*) and fluorescence excitation (*blue dashed*) spectra of compound **P1** in CH_2Cl_2.

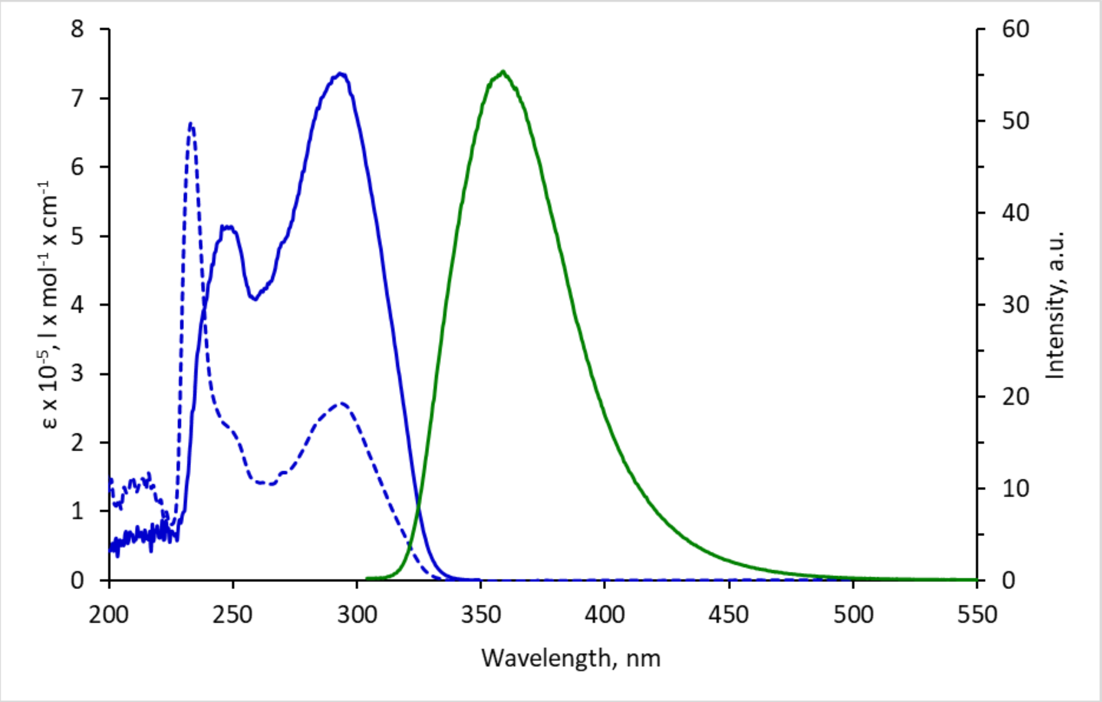

Figure S18. Absorption (*blue*), fluorescence emission (*green*) and fluorescence excitation (*blue dashed*) spectra of compound **P2** in CH_2Cl_2.

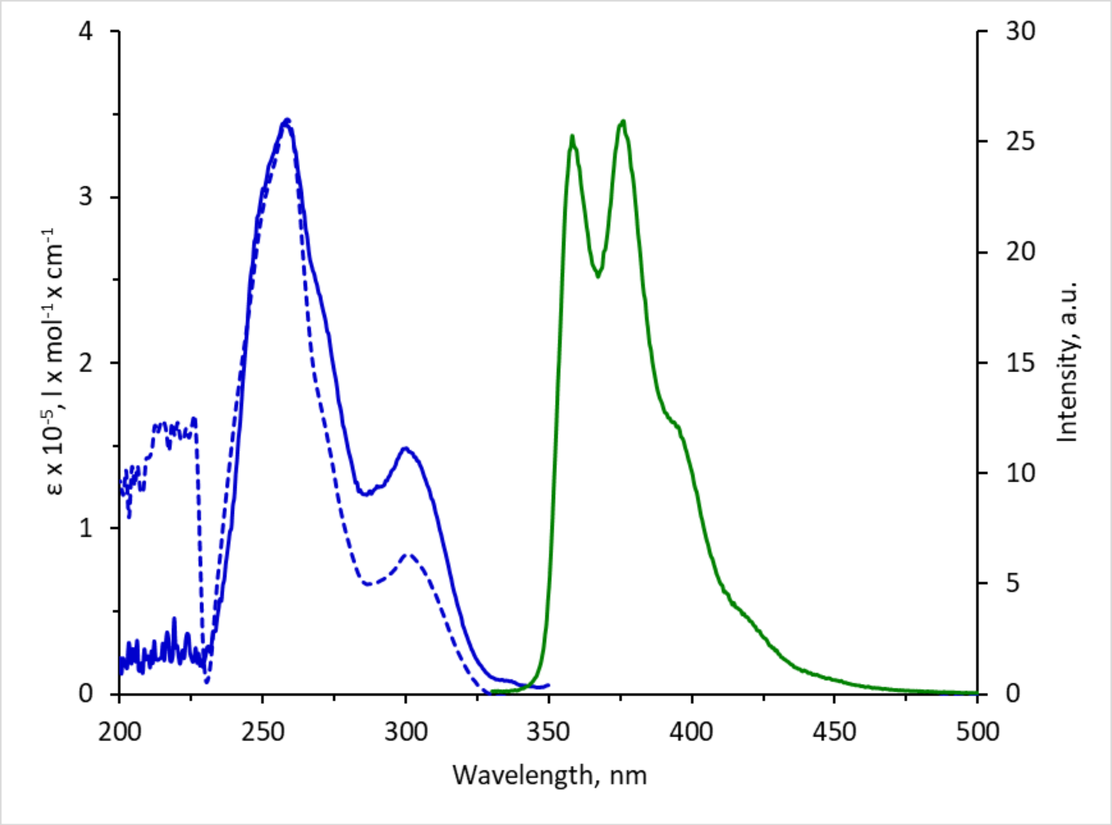

Figure S19. Absorption (*blue*), fluorescence emission (*green*) and fluorescence excitation (*blue dashed*) spectra of compound **P3*** in CH_2Cl_2.

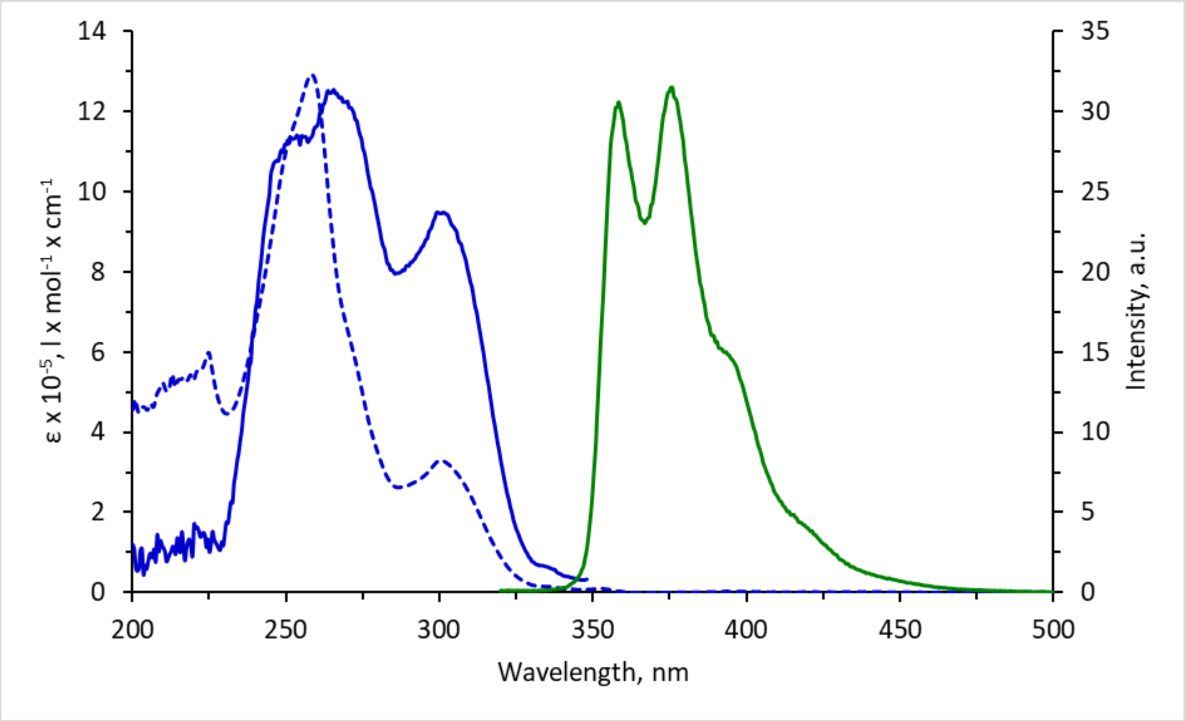

Figure S20. Absorption (*blue*), fluorescence emission (*green*) and fluorescence excitation (*blue dashed*) spectra of compound **P3** in CH_2Cl_2.

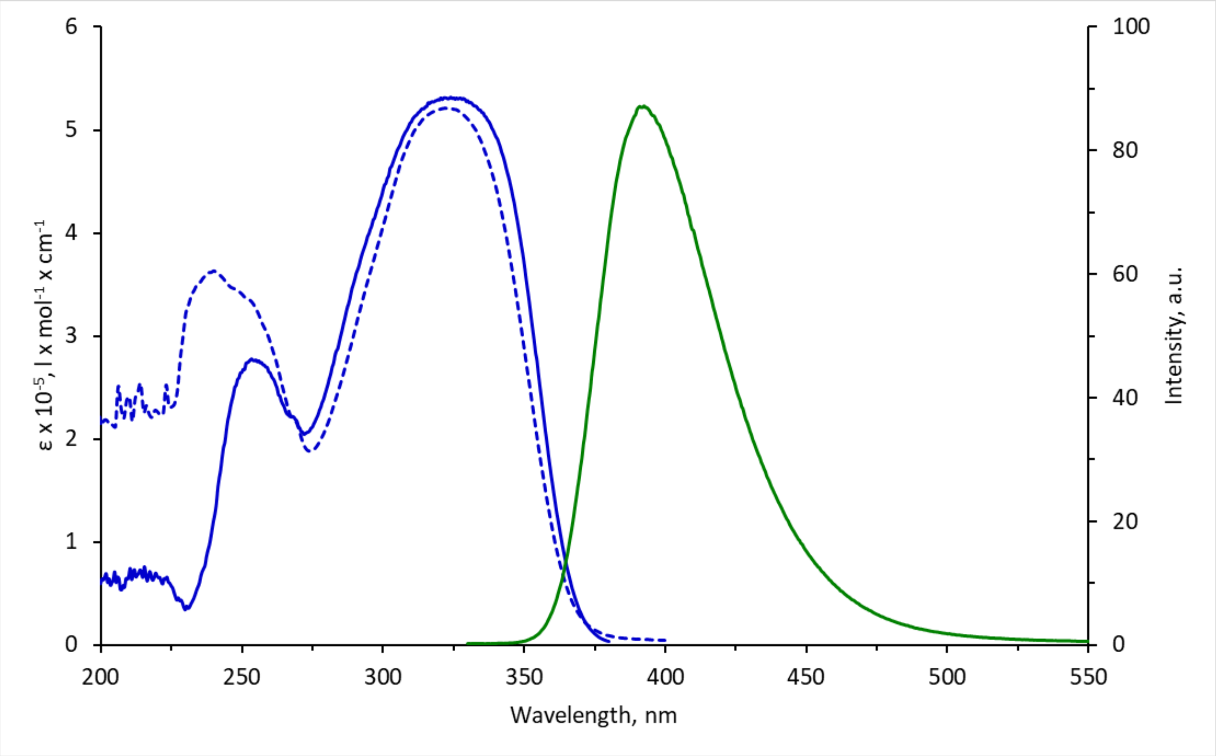

Figure S21. Absorption (*blue*), fluorescence emission (*green*) and fluorescence excitation (*blue dashed*) spectra of compound **P5** in CH_2Cl_2.

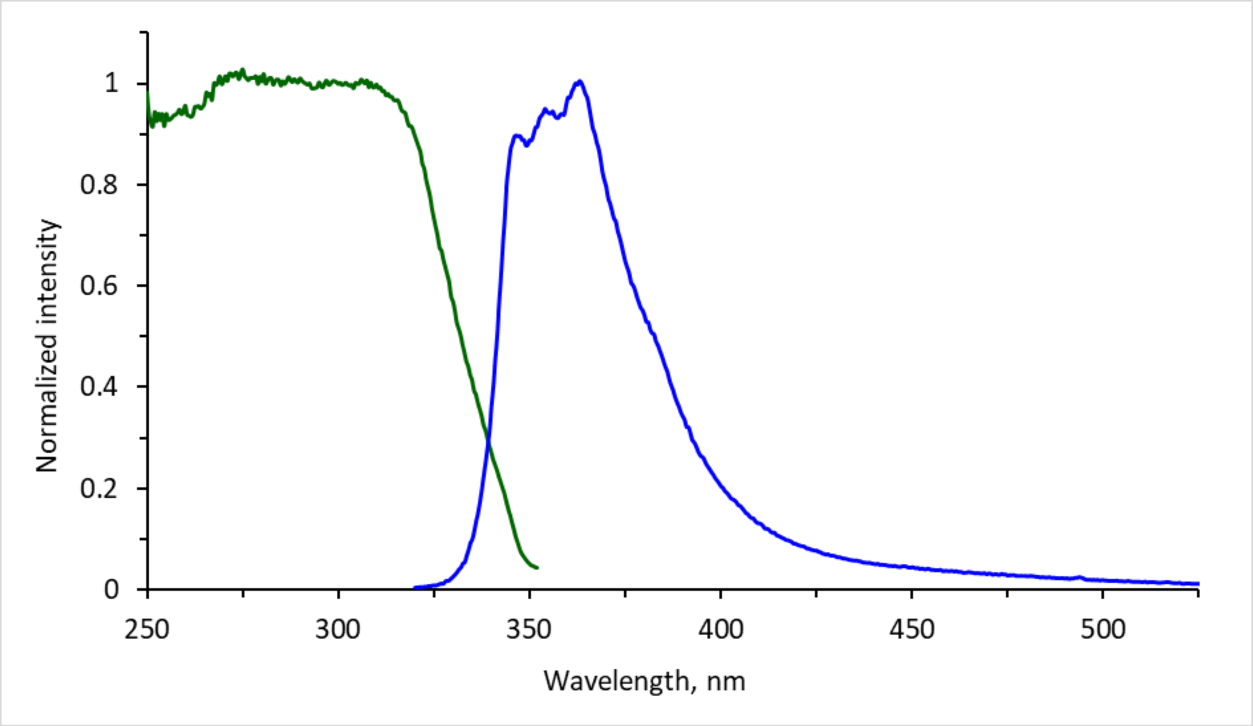

Figure S22. Excitation (*green*) and emission (*blue*) spectra of solid powder **P1**.

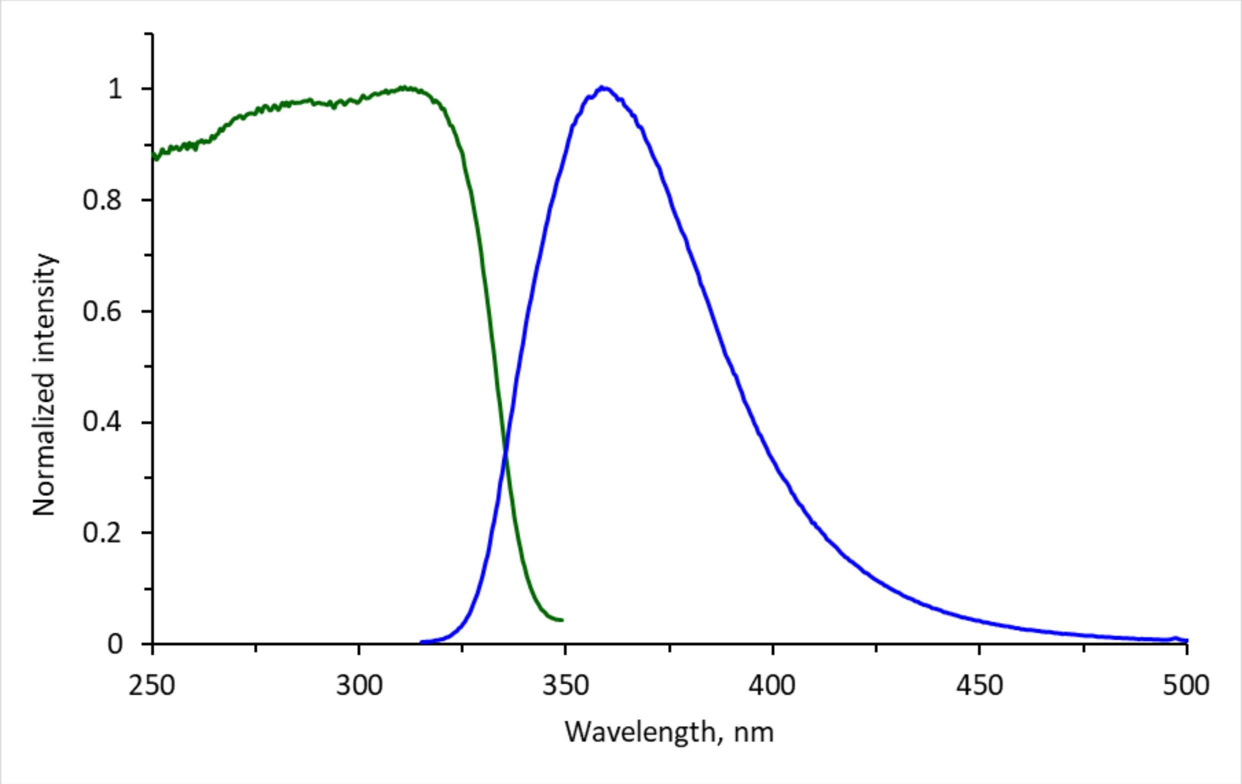

Figure S23. Excitation (*green*) and emission (*blue*) spectra of solid powder **P2**.

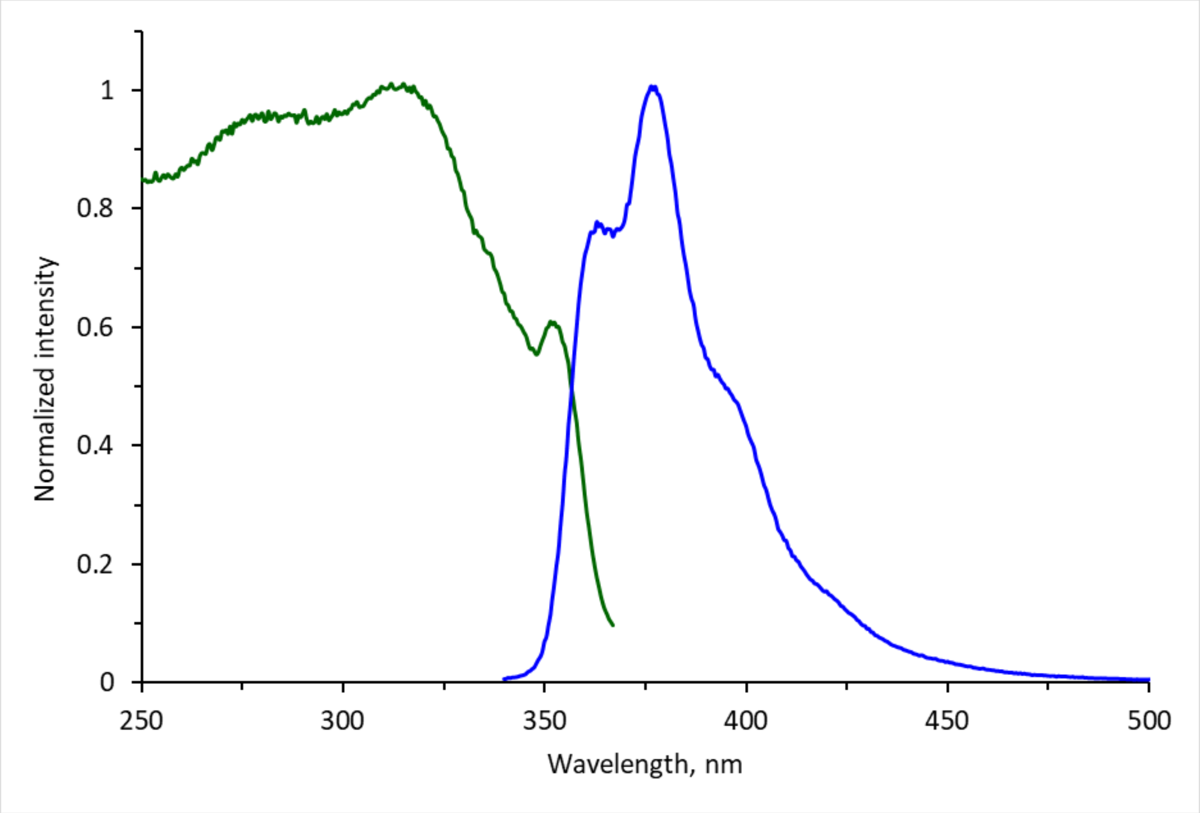

Figure S24. Excitation (*green*) and emission (*blue*) spectra of solid powder **P3**.

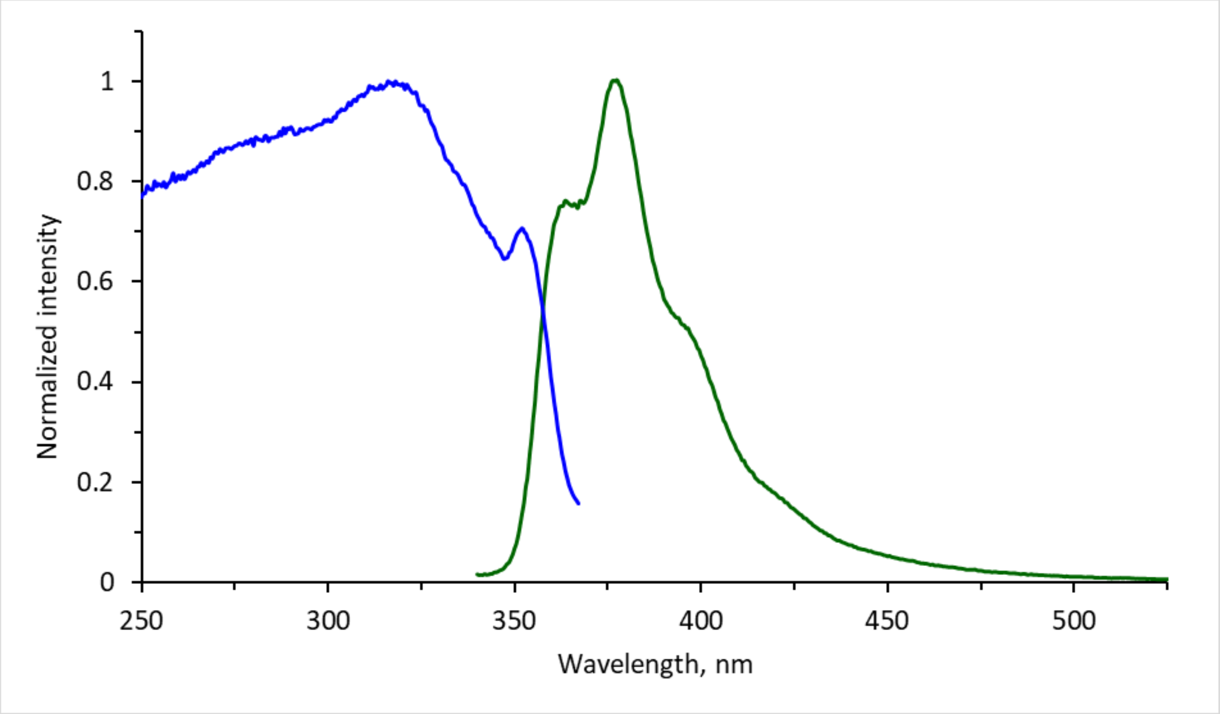

Figure S25. Excitation (*blue*) and emission (*green*) spectra of solid powder **P3***.

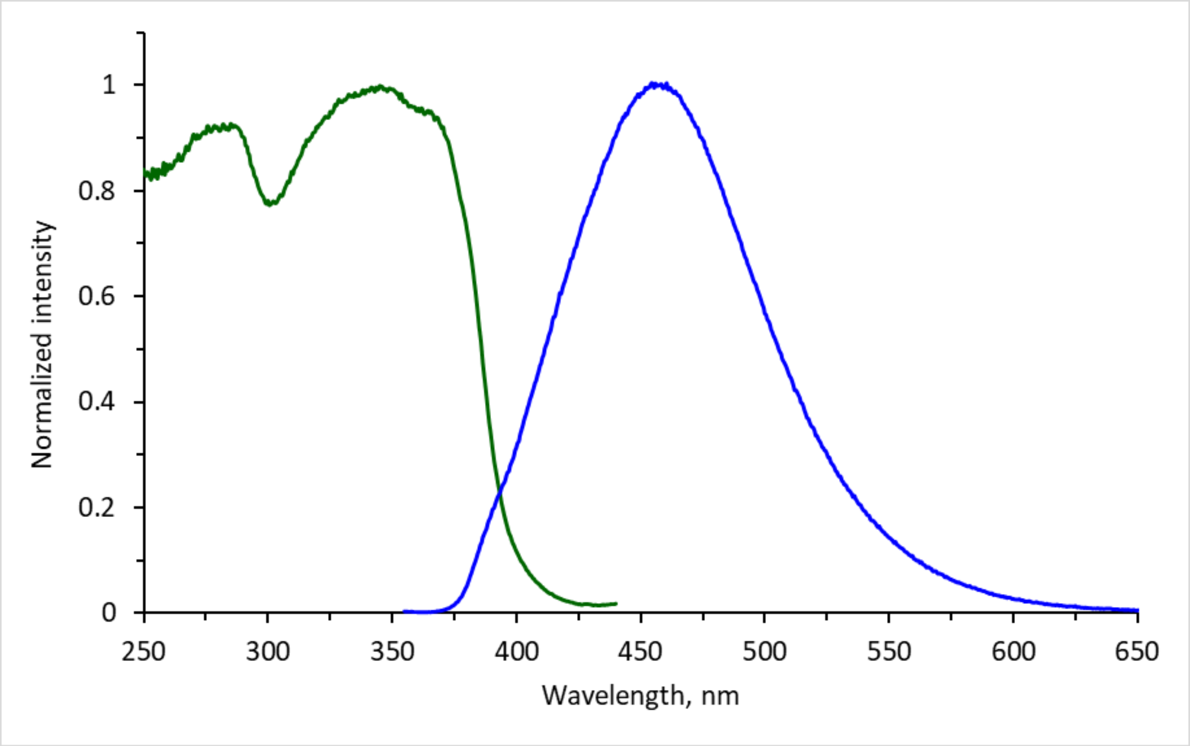

Figure S26. Excitation (*green*) and emission (*blue*) spectra of solid powder **P4**.

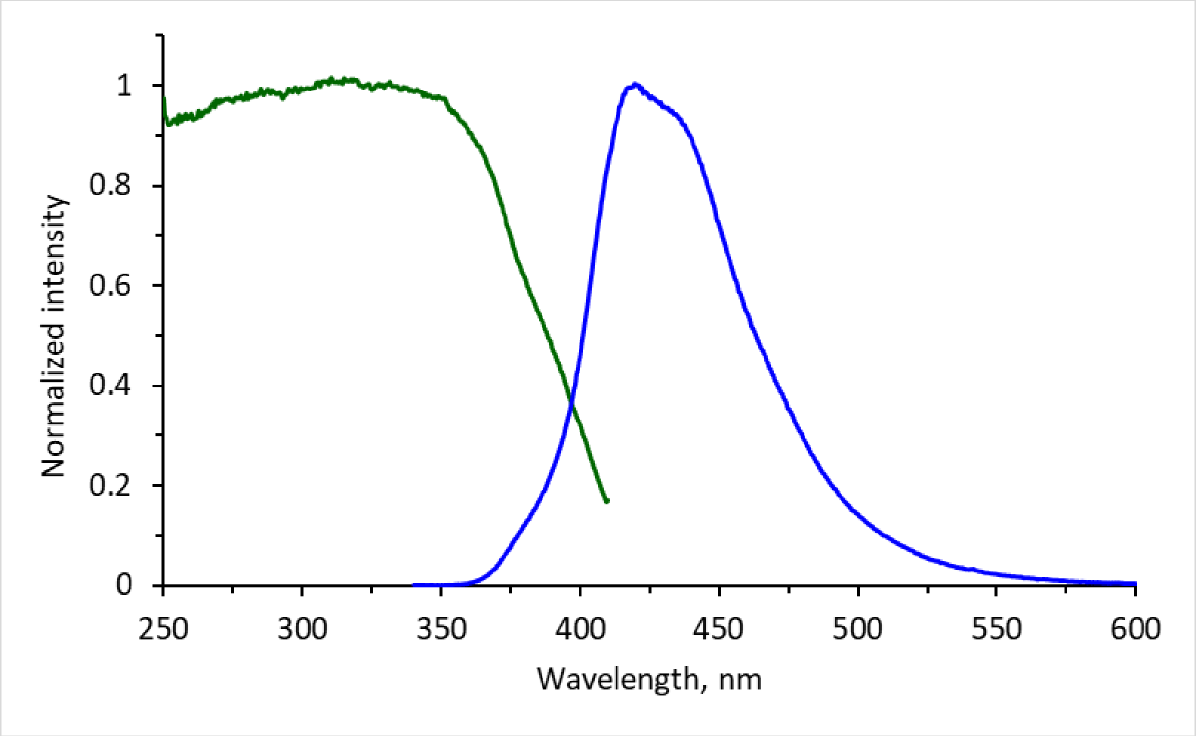

Figure S27. Excitation (*green*) and emission (*blue*) spectra of solid powder **P5.**

Table S2. Detailed data of the fluorescence lifetime measurements of **P1-P5** in DCM: τ – lifetime, *f* - fractional contribution, τ_{avg} – average lifetime, χ^2 - chi-squared distribution.

Polymer	Emission λ_{em} [nm]	τ_1, [ns]	f_1, %	τ_2, [ns]	f_2, %	τ_3, [ns]	f_3, %	τ_{avg}, [ns]	χ^2
**P1**	362	-	-	7.88	8.1	22.36	91.9	21.19	1.250
	345	-	-	9.61	10.3	22.98	89.7	21.60	1.060
**P2**	358	1.80	92.2	5.09	7.8	-	-	2.06	1.117
**P3**	376	-	-	14.57	100	-	-	14.57	1.040
	359	-	-	14.74	100	-	-	14.74	1.171
**P3***	376	-	-	14.41	100	-	-	14.41	1.063
	359	-	-	14.36	100	-	-	14.36	1.114
**P4**	478	-	-	-	-	25.23	100	25.23	1.182
	401	1.79	7.8	8.00	57.1	19.23	35.1	11.46	1.063
	383	2.20	8.8	8.25	56.8	18.84	34.4	11.35	1.032
**P5**	392	1.09	98.0	2.73	2	-	-	1.12	1.197

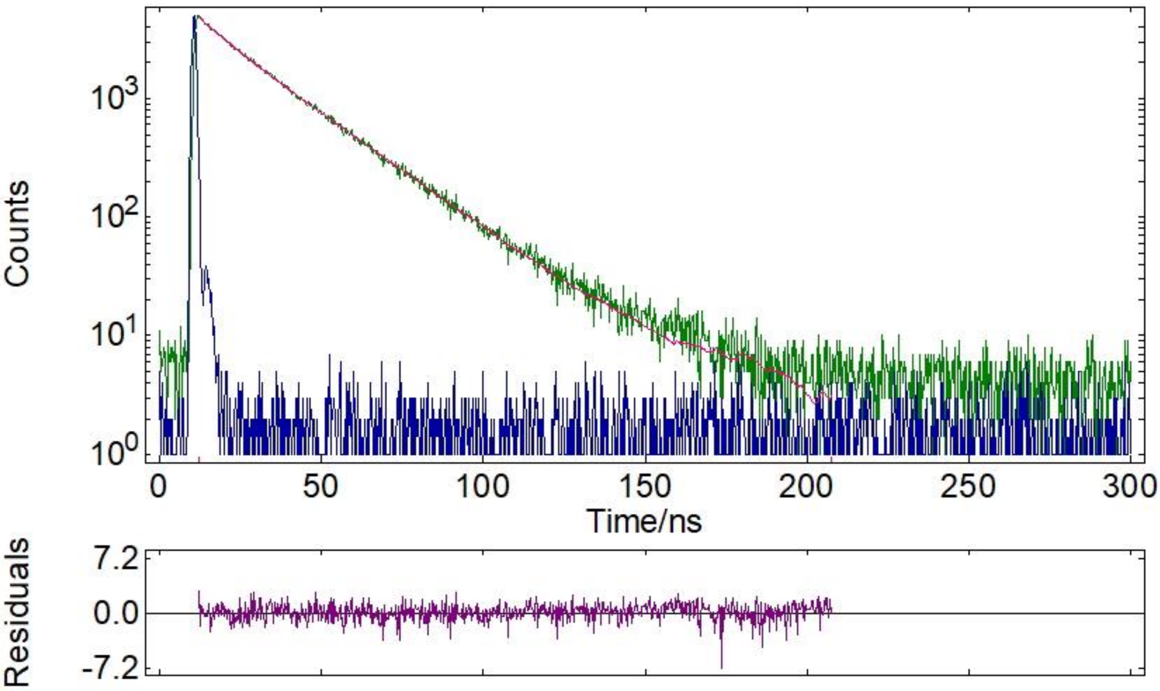

Figure S28. Time-resolved fluorescence lifetime decay profile of **P1** (*green*), instrumental response function (IRF, *blue*). λ_{ex} = 300 nm, λ_{ex} = 362 nm.

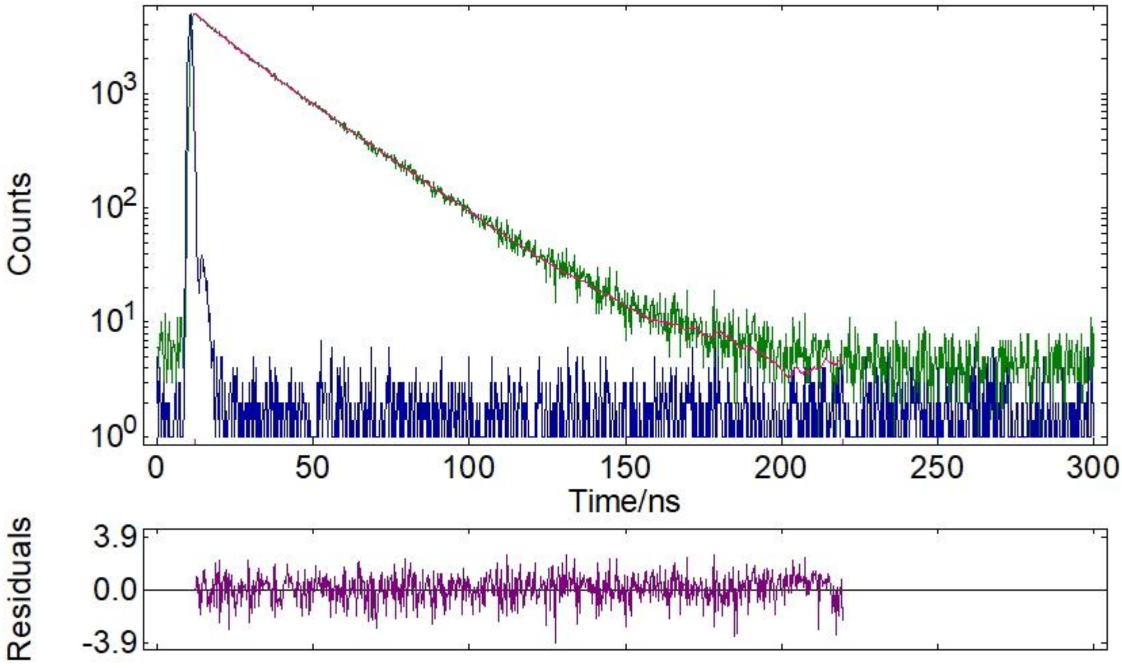

Figure S29. Time-resolved fluorescence lifetime decay profile of **P1** (green)), IRF (*blue*). λ_{ex} = 300 nm, λ_{ex} = 345 nm.

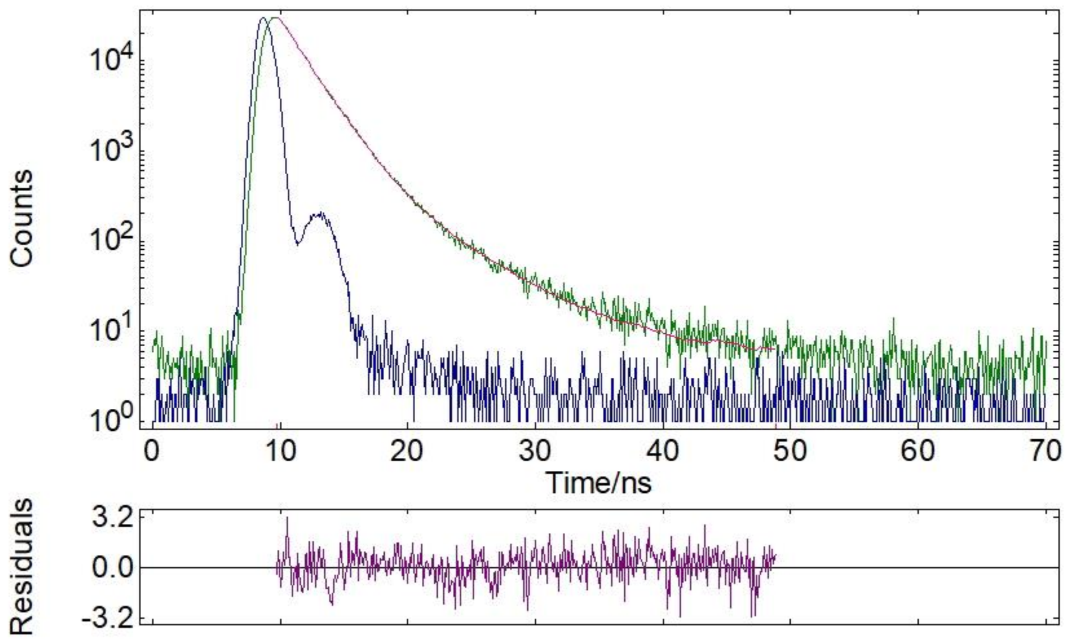

Figure S30. Time-resolved fluorescence lifetime decay profile of **P2** (*green*), IRF (*blue*). λ_{ex} = 300 nm, λ_{ex} = 358 nm.

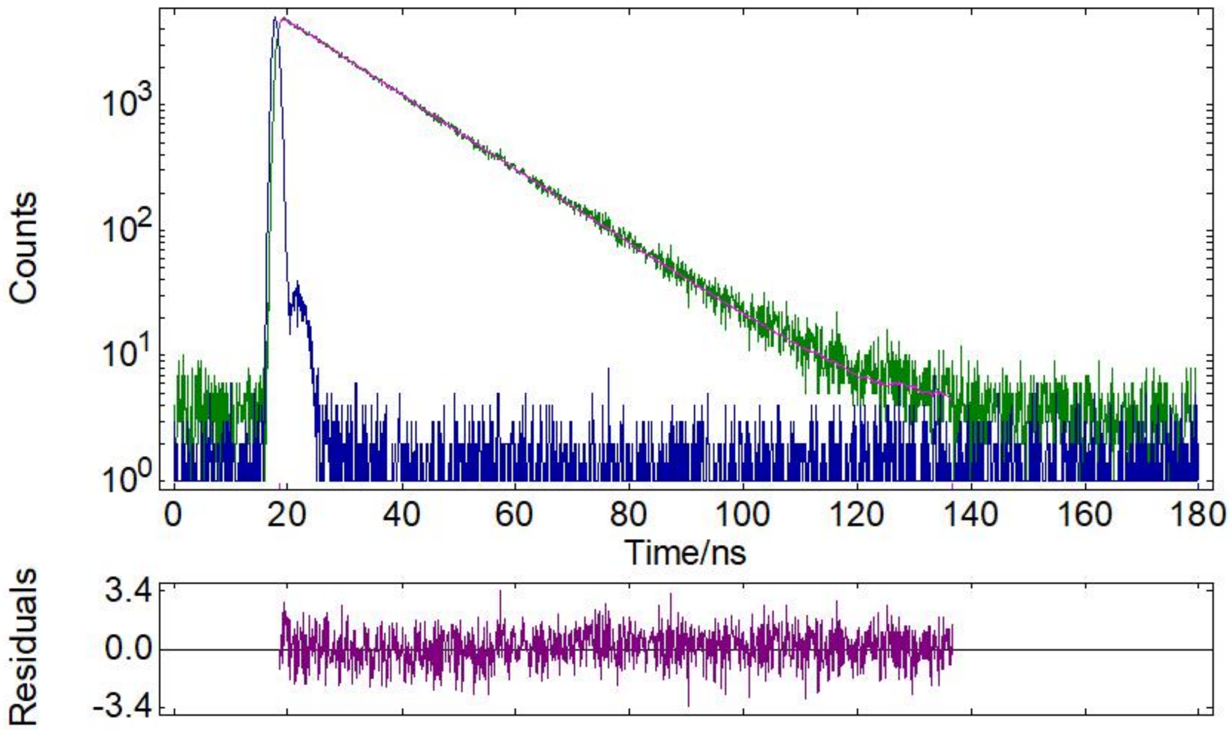

Figure S31. Time-resolved fluorescence lifetime decay profile of **P3** (*green*), IRF (*blue*). λ_{ex} = 300 nm, λ_{ex} = 376 nm.

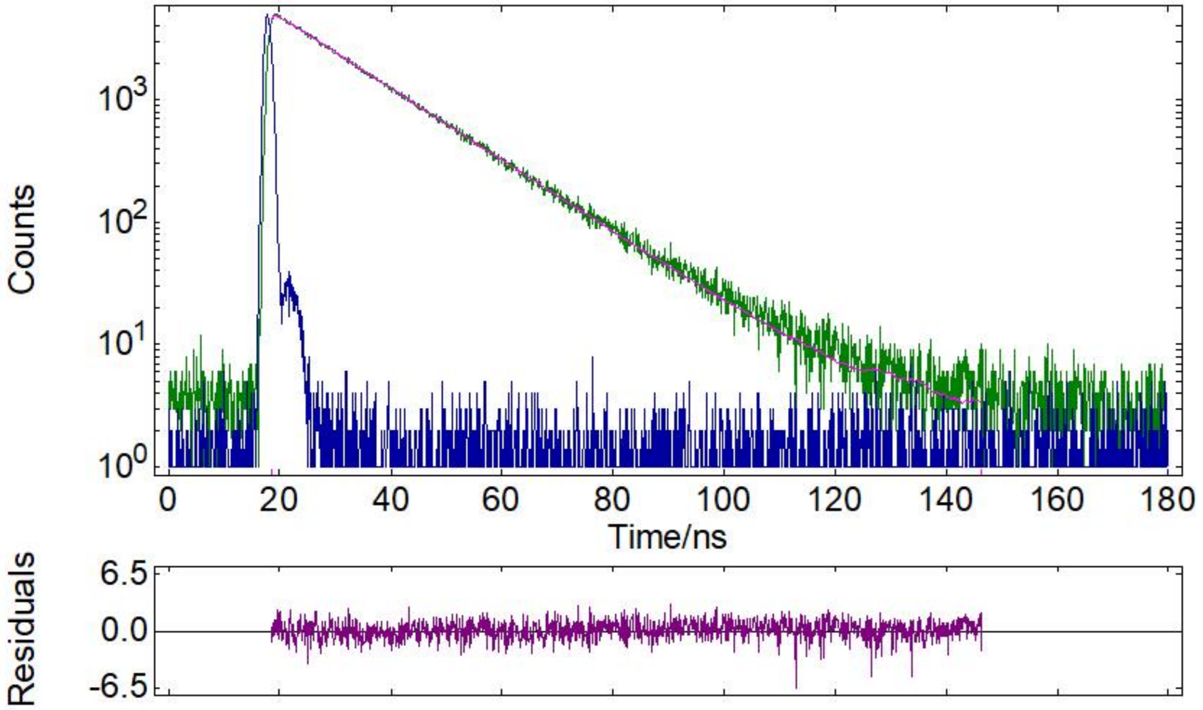

Figure S32. Time-resolved fluorescence lifetime decay profile of **P3** (*green*), IRF (*blue*). λ_{ex} = 300 nm, λ_{ex} = 359 nm.

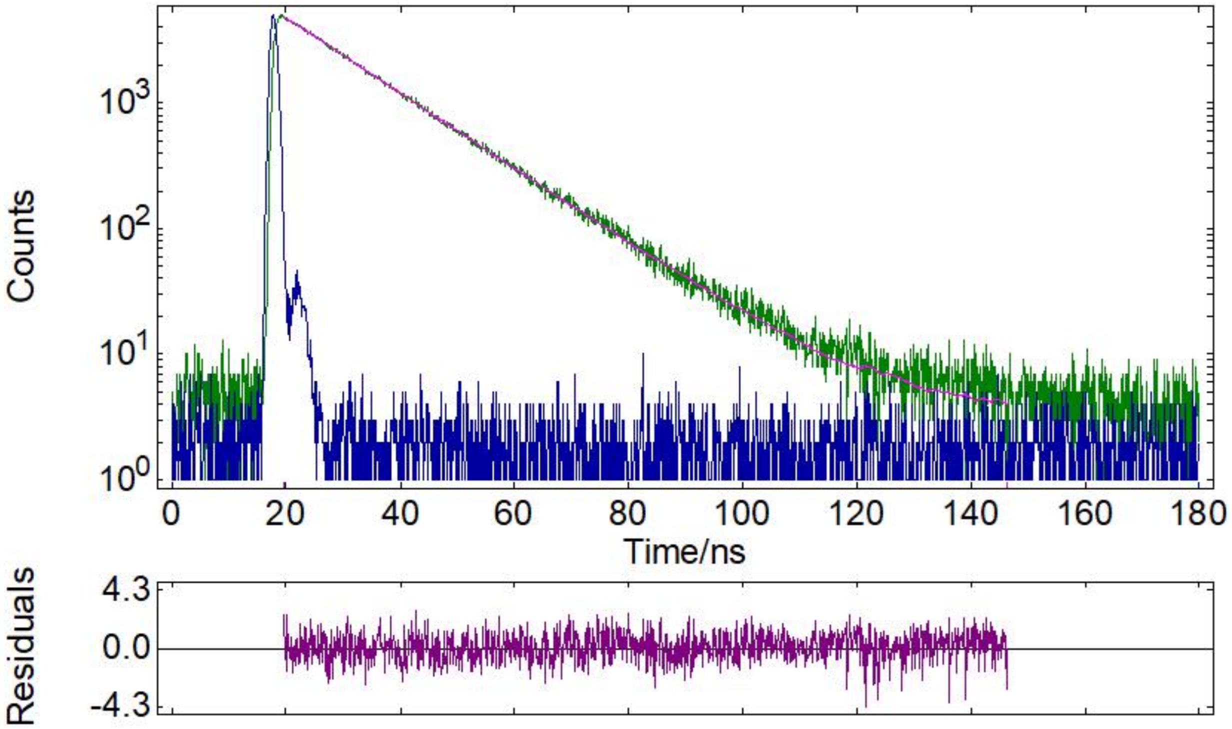

Figure S33. Time-resolved fluorescence lifetime decay profile of **P3*** (*green*), IRF (*blue*). λ_{ex} = 300 nm, λ_{ex} = 376 nm.

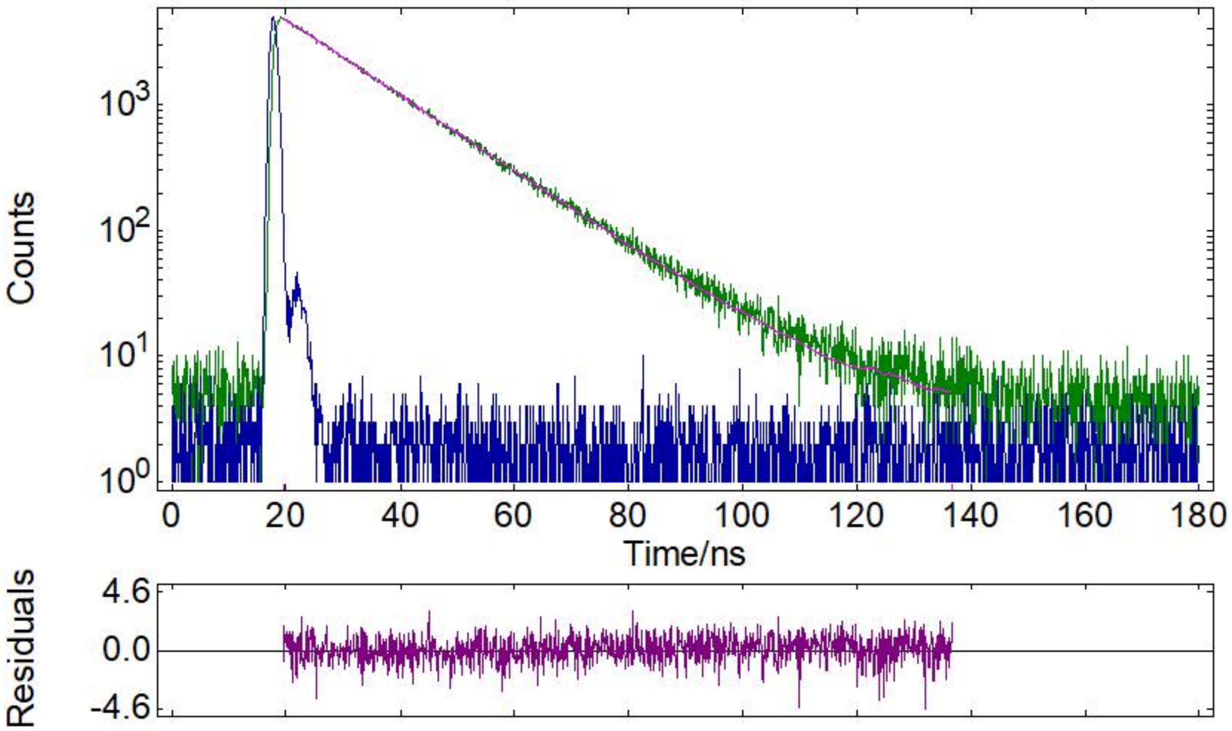

Figure S34. Time-resolved fluorescence lifetime decay profile of **P3*** (*green*), IRF (*blue*). λ_{ex} = 300 nm, λ_{ex} = 359 nm.

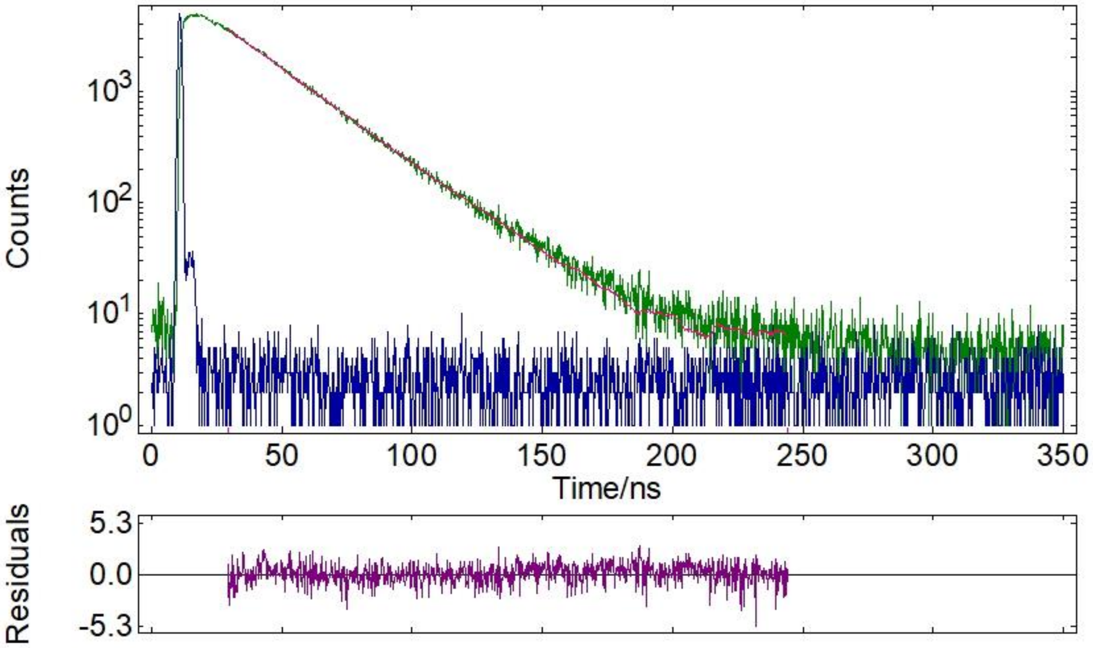

Figure S35. Time-resolved fluorescence lifetime decay profile of **P4** (*green*), IRF (*blue*). λ_{ex} = 300 nm, λ_{ex} = 478 nm.

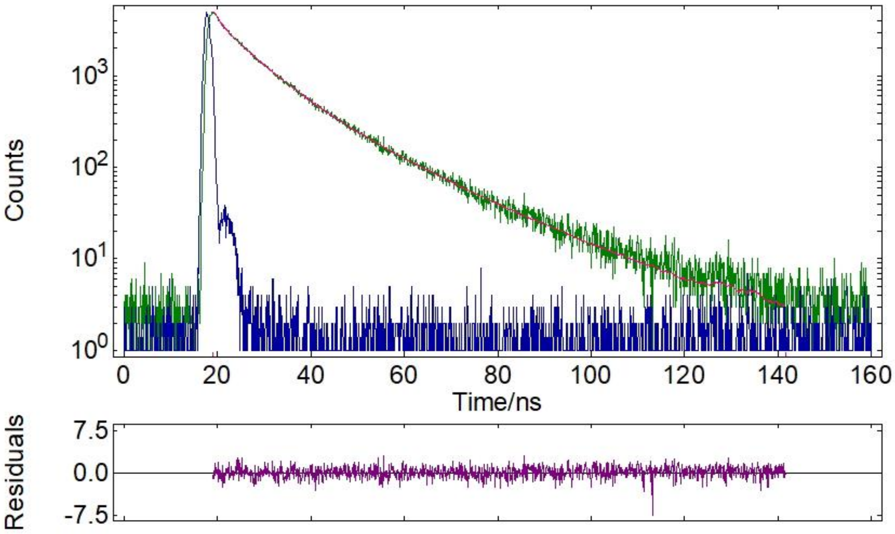

Figure S36. Time-resolved fluorescence lifetime decay profile of **P4** (*green*), IRF (*blue*). λ_{ex} = 300 nm, λ_{ex} = 401 nm.

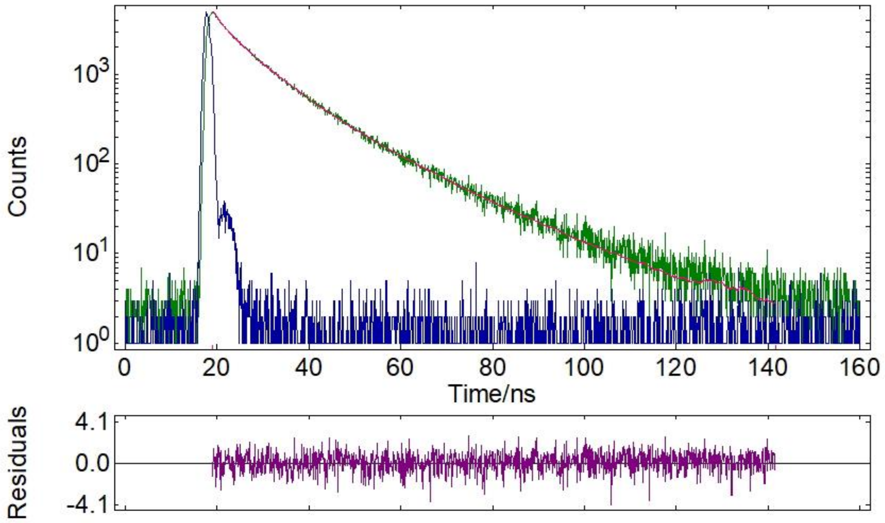

Figure S37. Time-resolved fluorescence lifetime decay profile of **P4** (*green*), IRF (*blue*). λ_{ex} = 300 nm, λ_{ex} = 383 nm.

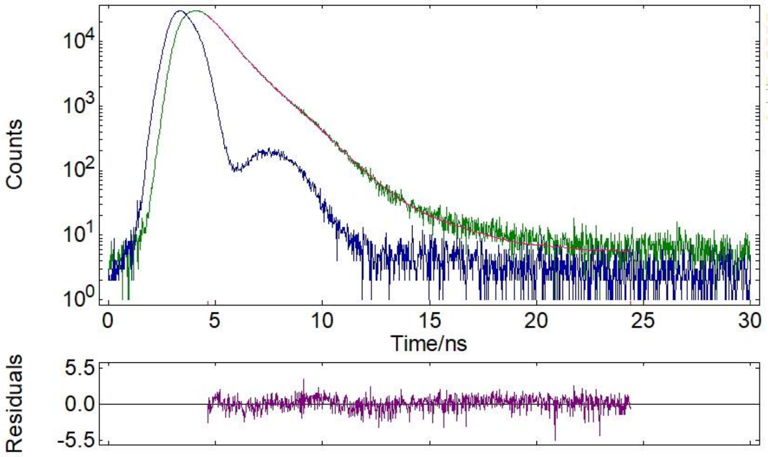

Figure S38. Time-resolved fluorescence lifetime decay profile of **P5** (*green*), IRF (*blue*). λ_{ex} = 300 nm, λ_{ex} = 392 nm.

Table S3. Detailed data of the fluorescence lifetime measurements of solid powders **P1-P5**: τ – lifetime, *f* - fractional contribution, τ_{avg} – average lifetime, χ^2 - chi-squared distribution.

Polymer	Emission λ_{em} [nm]	τ_1, [ns]	f_1, %	τ_2, [ns]	f_2, %	τ_3, [ns]	f_3, %	τ_4, [ns]	f_4, %	τ_{avg}, [ns]	χ^2
**P1**	362	-	-	-	-	24.78	30.0	48.31	70.0	41.25	1.181
	355	-	-	-	-	19.73	20.0	45.46	80.0	40.31	1.076
	346	-	-	-	-	18.34	17.0	44.45	83.0	40.02	1.140
**P2**	359	2.04	97.5	6.39	2.5	-	-	-	-	2.15	1.044
**P3**	377	-	-	14.45	8.3	29.10	91.7	-	-	27.88	1.082
	364	-	-	13.74	4.50	28.62	95.5	-	-	27.95	1.133
**P3***	377	-	-	14.75	8.8	28.77	91.2	-	-	27.53	1.132
	364	-	-	14.72	7.8	28.27	92.2	-	-	27.21	1.046
**P4**	457	-	-	4.85	18.6	19.42	57.6	47.92	23.8	23.50	1.112
**P5**	420	1.08	77.3	2.15	22.3	-	-	-	-	1.32	1.021

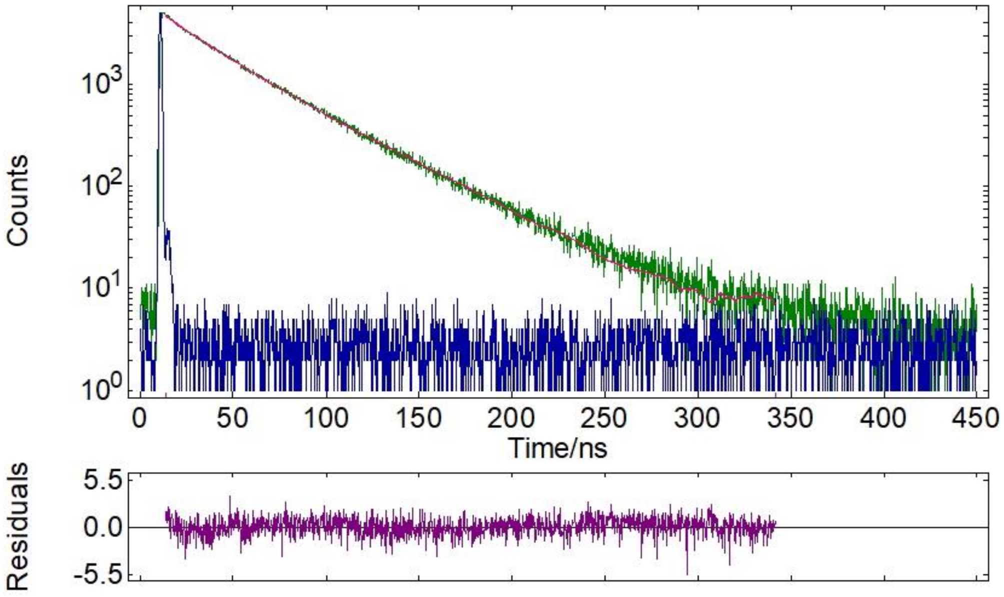

Figure S39. Time-resolved fluorescence lifetime decay profile of solid **P1** (*green*), instrumental response function (IRF, *blue*). λ_{ex} = 300 nm, λ_{ex} = 362 nm.

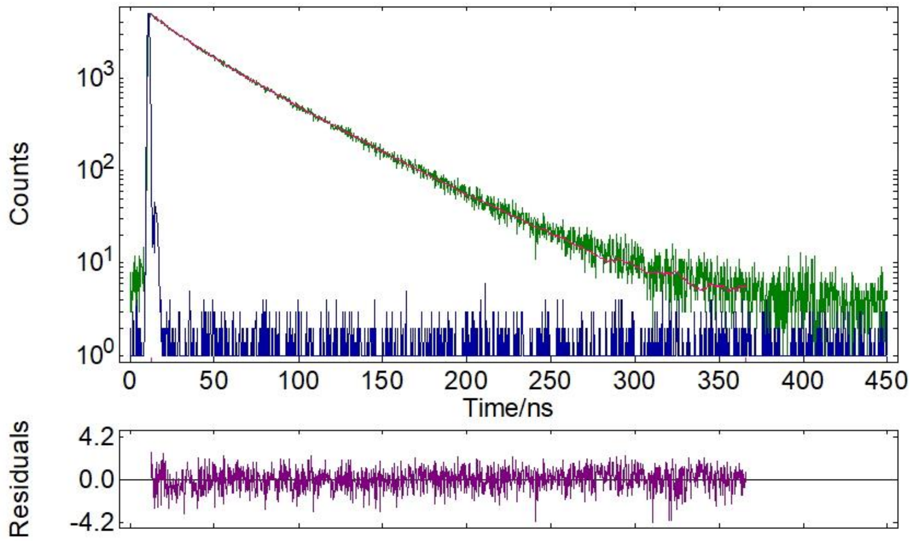

Figure S40. Time-resolved fluorescence lifetime decay profile of solid **P1** (*green*), instrumental response function (IRF, *blue*). λ_{ex} = 300 nm, λ_{ex} = 355 nm.

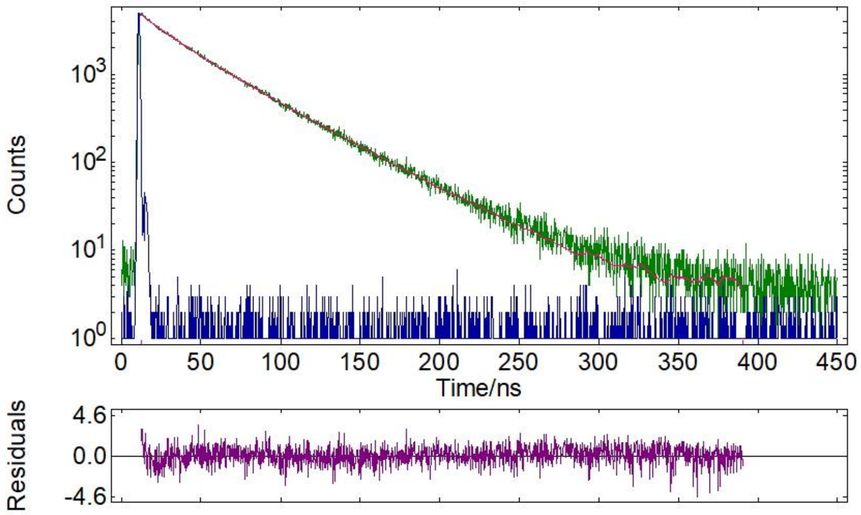

Figure S41. Time-resolved fluorescence lifetime decay profile of solid **P1** (*green*), instrumental response function (IRF, *blue*). λ_{ex} = 300 nm, λ_{ex} = 346 nm.

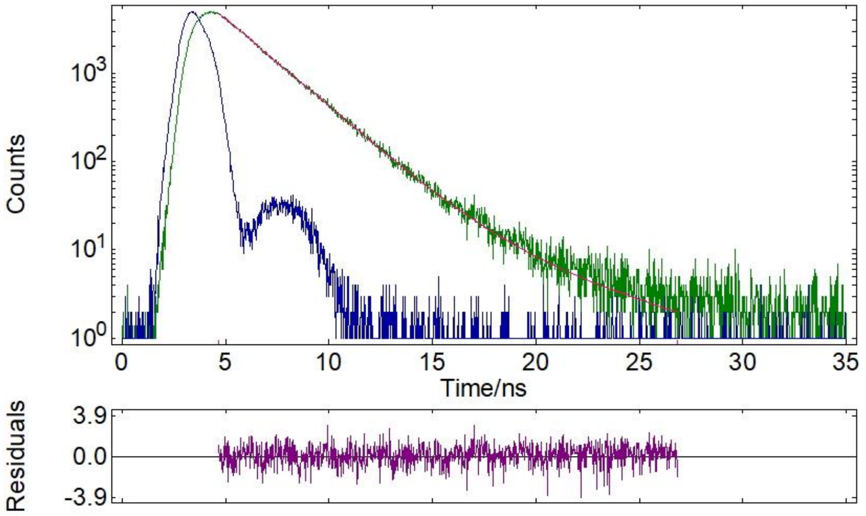

Figure S42. Time-resolved fluorescence lifetime decay profile of solid **P2** (*green*), instrumental response function (IRF, *blue*). λ_{ex} = 300 nm, λ_{ex} = 359 nm.

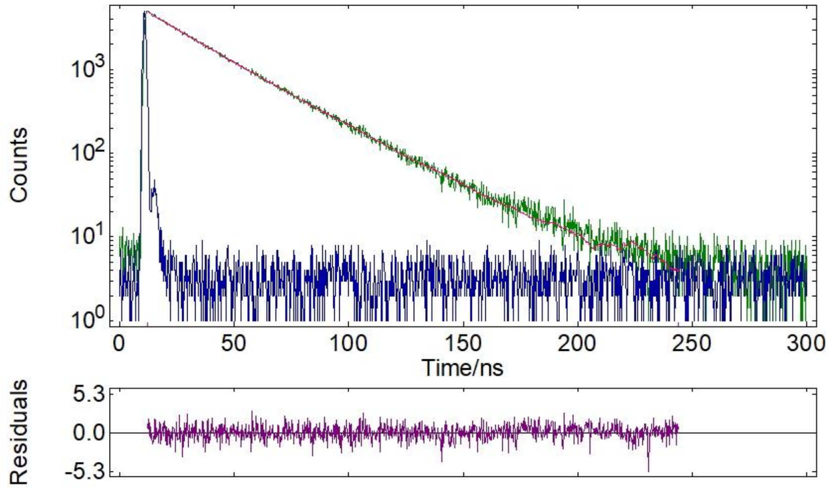

Figure S43. Time-resolved fluorescence lifetime decay profile of solid **P3** (*green*), instrumental response function (IRF, *blue*). λ_{ex} = 300 nm, λ_{ex} = 377 nm.

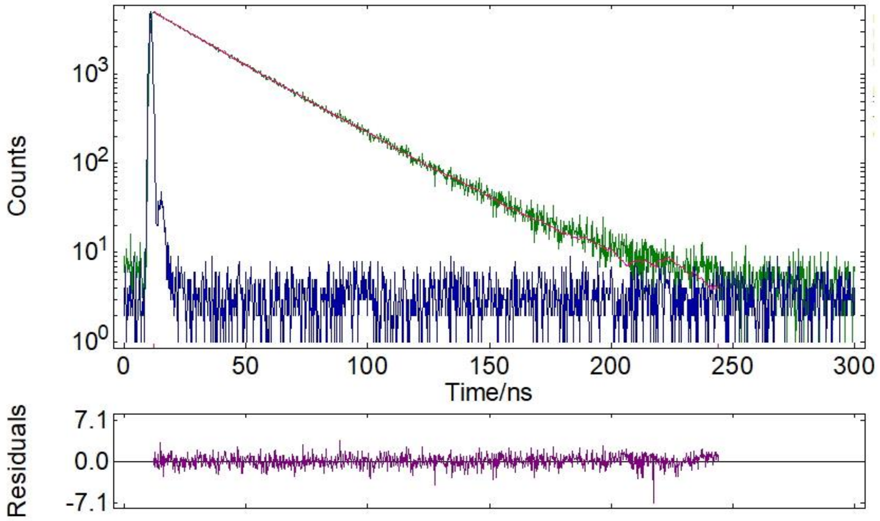

Figure S44. Time-resolved fluorescence lifetime decay profile of solid **P3** (*green*), instrumental response function (IRF, *blue*). λ_{ex} = 300 nm, λ_{ex} = 364 nm.

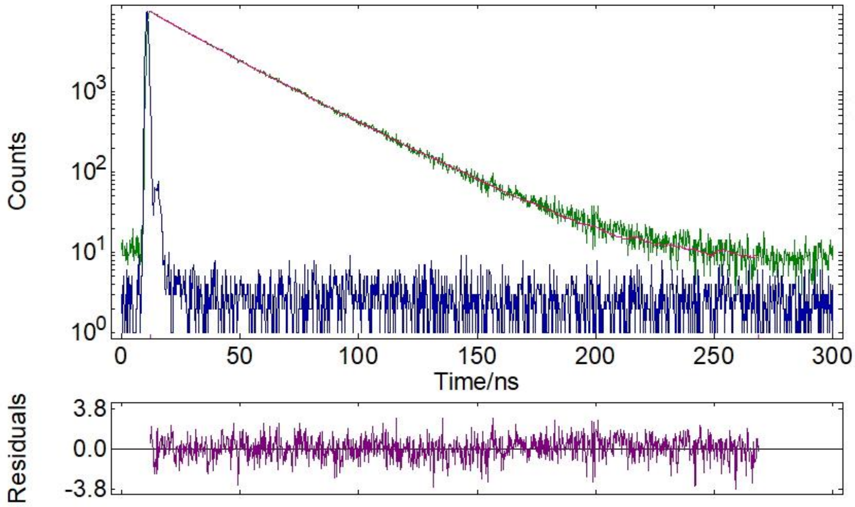

Figure S45. Time-resolved fluorescence lifetime decay profile of solid **P3*** (*green*), instrumental response function (IRF, *blue*). λ_{ex} = 300 nm, λ_{ex} = 377 nm.

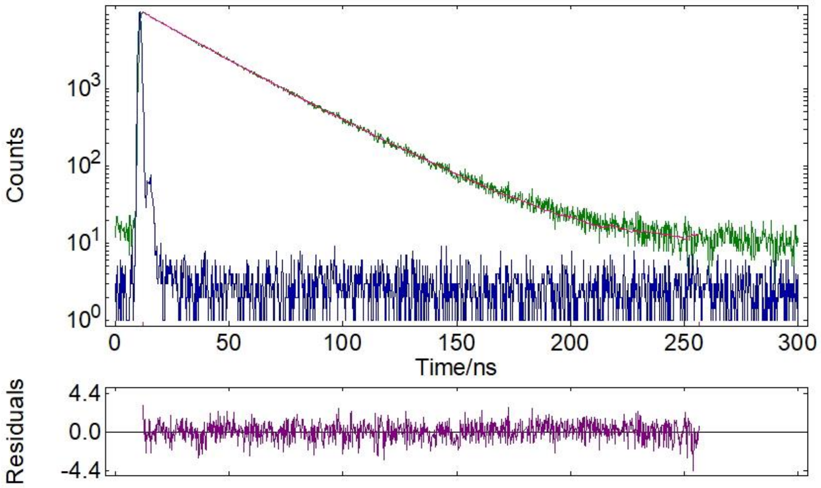

Figure S46. Time-resolved fluorescence lifetime decay profile of solid **P3*** (*green*), instrumental response function (IRF, *blue*). λ_{ex} = 300 nm, λ_{ex} = 364 nm.

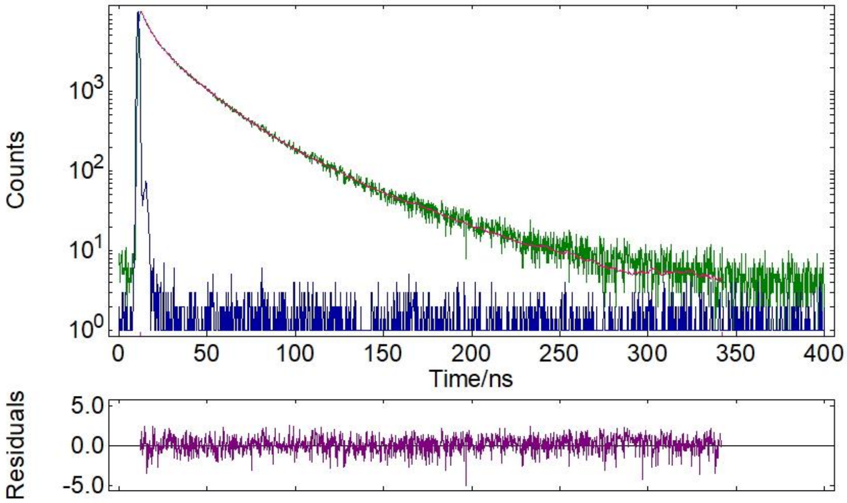

Figure S47. Time-resolved fluorescence lifetime decay profile of solid **P4** (*green*), instrumental response function (IRF, *blue*). λ_{ex} = 300 nm, λ_{ex} = 457 nm.

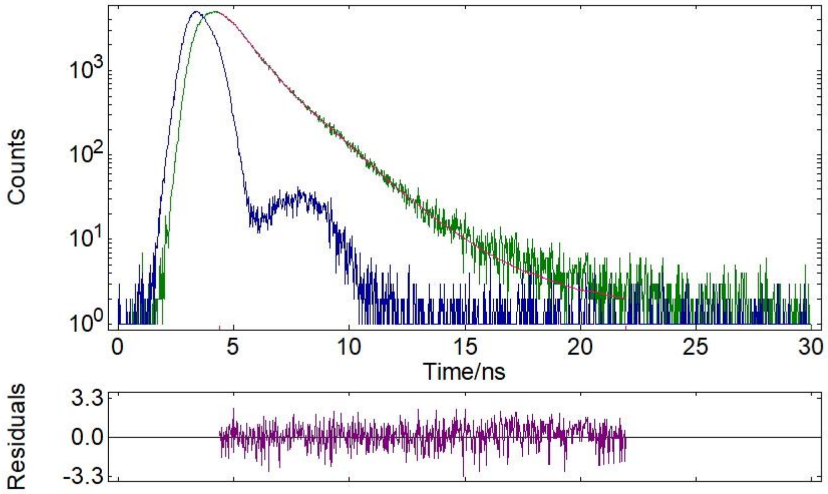

Figure S48. Time-resolved fluorescence lifetime decay profile of solid **P5** (*green*), instrumental response function (IRF, *blue*). λ_{ex} = 300 nm, λ_{ex} = 420 nm.

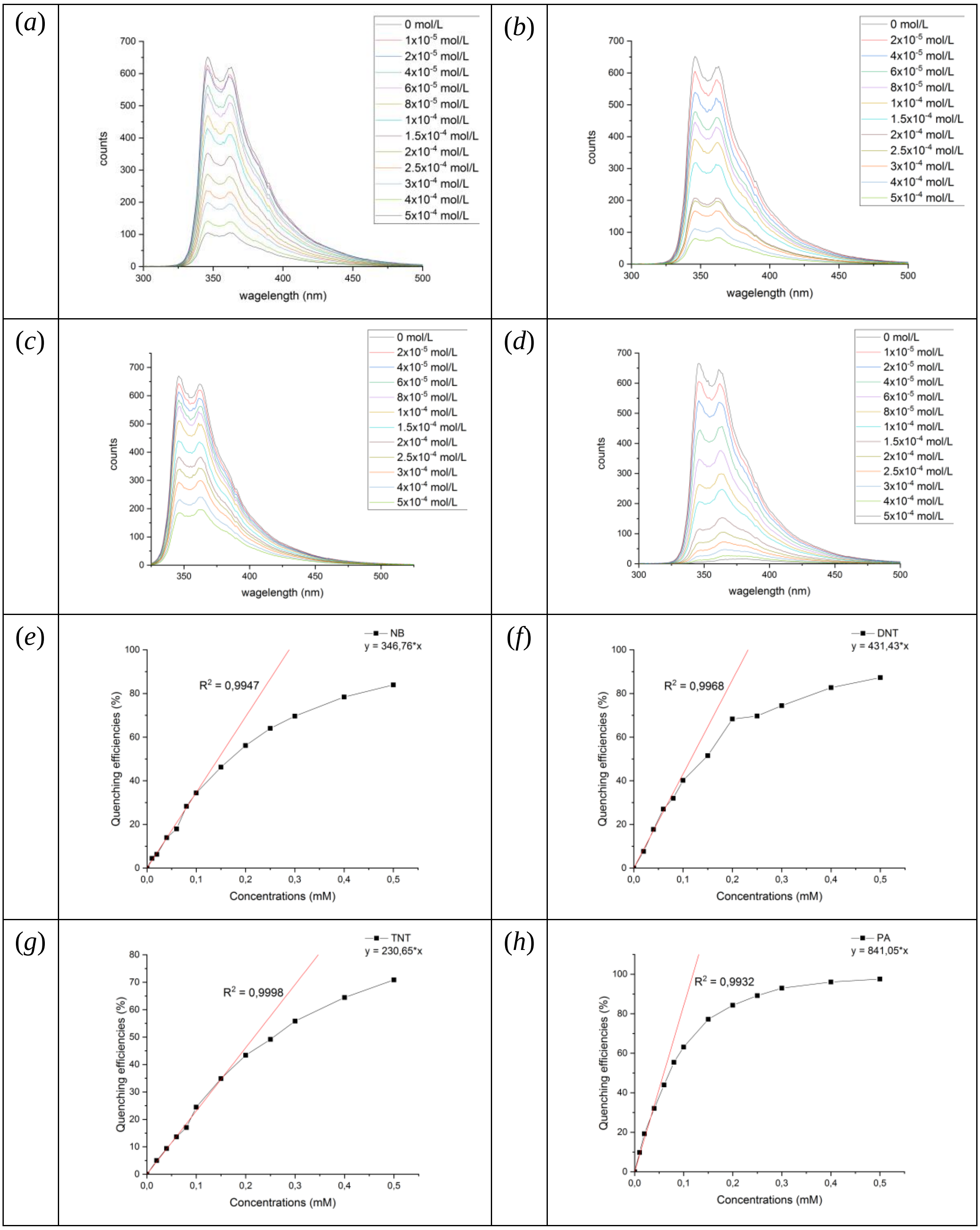

Figure S49. Fluorescence quenching studies of **P1** (1.0×10^{-5} mol/L) recorded in the presence of various amounts of NB (*a*), DNT (*b*), TNT (*c*), and PA (*d*), of which 290 nm was taken as the excitation wavelength. The Stern–Volmer plots as function of NB (*e*), DNT (*f*), TNT (*g*), and PA (*h*) concentration in CH_2Cl_2, with an excitation wavelength of 290 nm for **P1** solution.

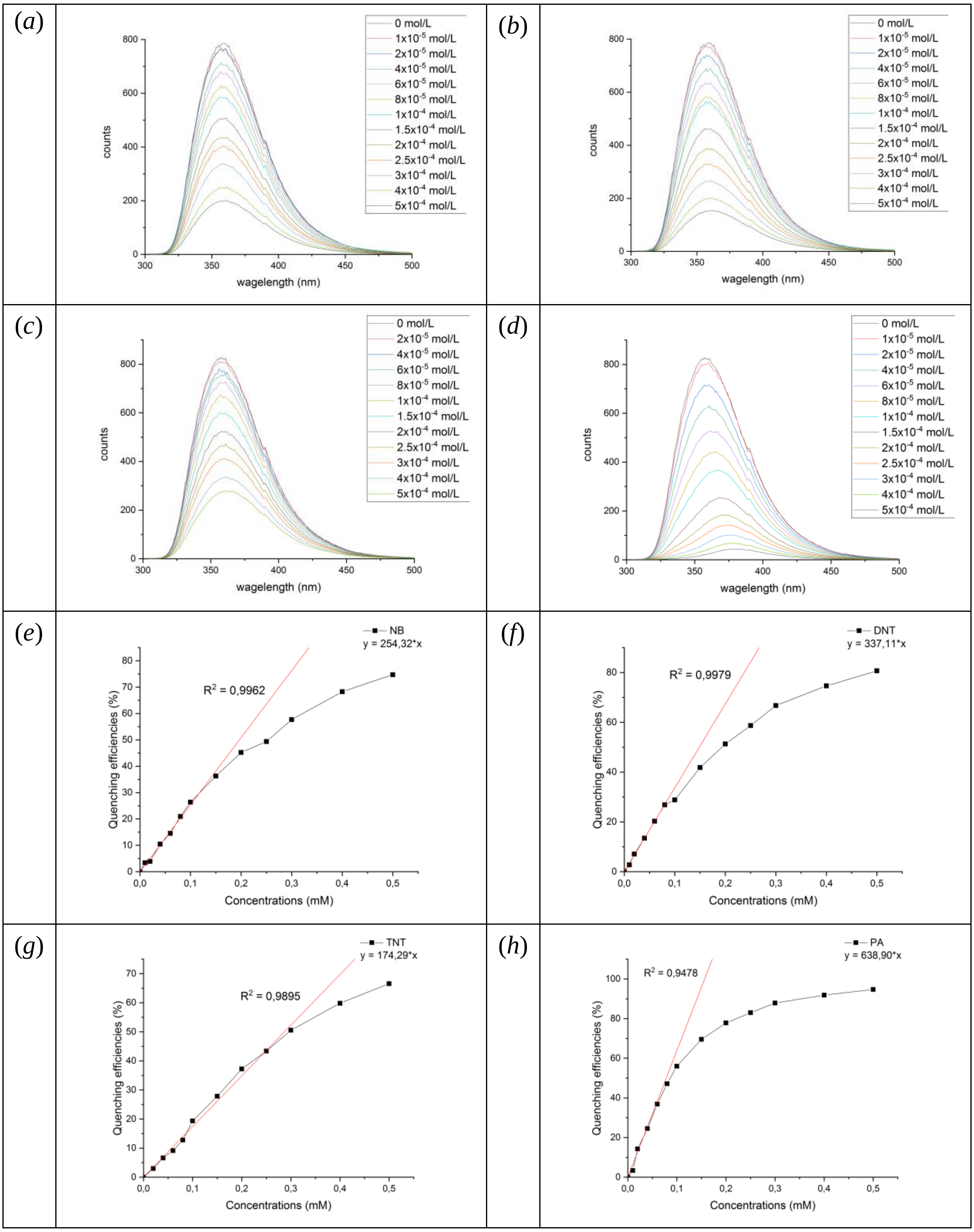

Figure S50. Fluorescence quenching studies of **P2** (1.0×10^{-5} mol/L) recorded in the presence of various amounts of NB (*a*), DNT (*b*), TNT (*c*), and PA (*d*), of which 294 nm was taken as the excitation wavelength. The Stern–Volmer plots as function of NB (*e*), DNT (*f*), TNT (*g*), and PA (*h*) concentration in CH_2Cl_2, with an excitation wavelength of 294 nm for **P2** solution.

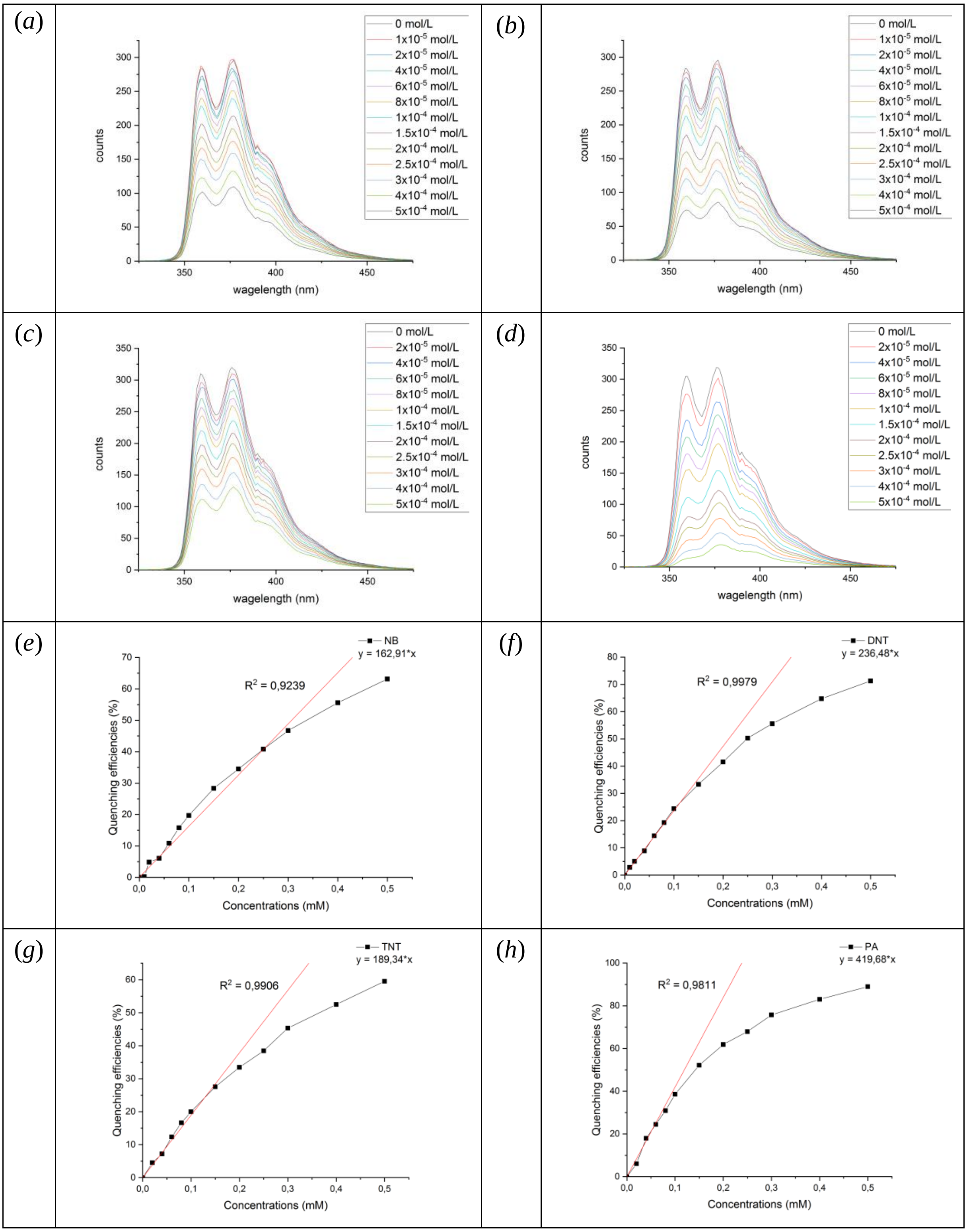

Figure S51. Fluorescence quenching studies of **P3** (1.0×10^{-5} mol/L) recorded in the presence of various amounts of NB (*a*), DNT (*b*), TNT (*c*), and PA (*d*), of which 301 nm was taken as the excitation wavelength. The Stern–Volmer plots as function of NB (*e*), DNT (*f*), TNT (*g*), and PA (*h*) concentration in CH_2Cl_2, with an excitation wavelength of 301 nm for **P3** solution.

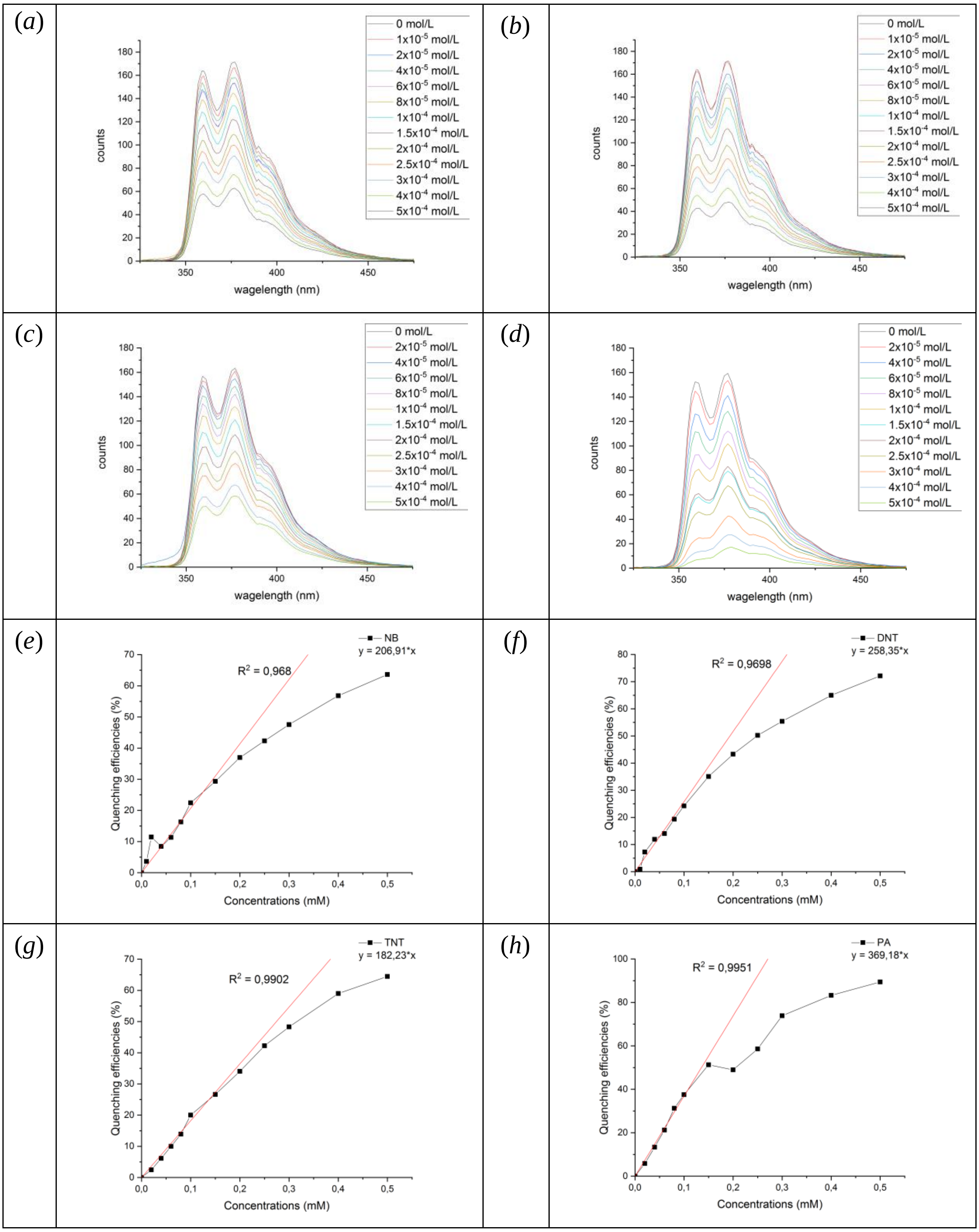

Figure S52. Fluorescence quenching studies of **P3*** (1.0×10^{-5} mol/L) recorded in the presence of various amounts of NB (*a*), DNT (*b*), TNT (*c*), and PA (*d*), of which 301 nm was taken as the excitation wavelength. The Stern–Volmer plots as function of NB (*e*), DNT (*f*), TNT (*g*), and PA (*h*) concentration in CH_2Cl_2, with an excitation wavelength of 301 nm for **P3*** solution.

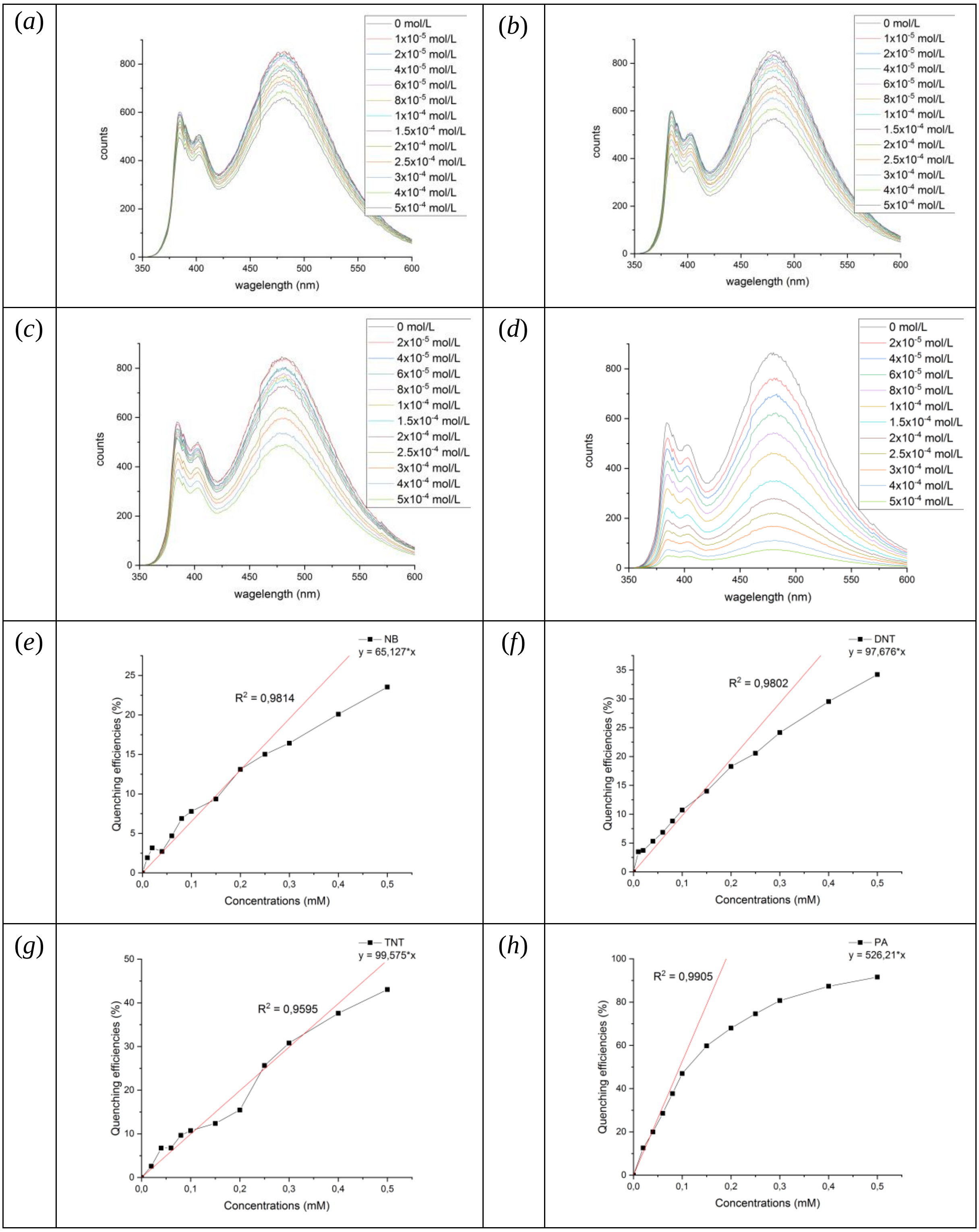

Figure S53. Fluorescence quenching studies of **P4** (1.0×10^{-5} mol/L) recorded in the presence of various amounts of NB (*a*), DNT (*b*), TNT (*c*), and PA (*d*), of which 345 nm was taken as the excitation wavelength. The Stern–Volmer plots as function of NB (*e*), DNT (*f*), TNT (*g*), and PA (*h*) concentration in CH_2Cl_2, with an excitation wavelength of 345 nm for **P4** solution.

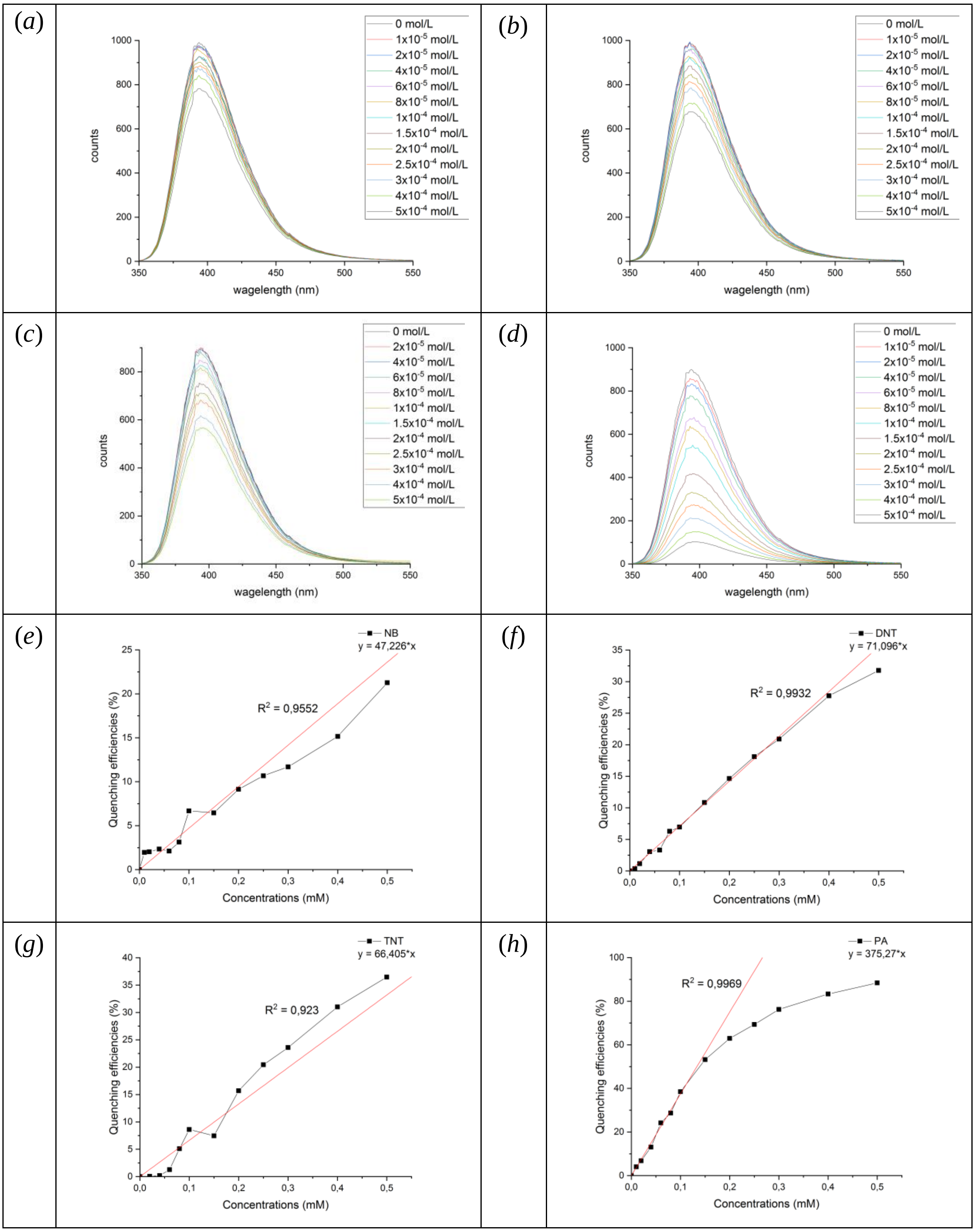

Figure S54. Fluorescence quenching studies of **P5** (1.0×10^{-5} mol/L) recorded in the presence of various amounts of NB (*a*), DNT (*b*), TNT (*c*), and PA (*d*), of which 325 nm was taken as the excitation wavelength. The Stern–Volmer plots as function of NB (*e*), DNT (*f*), TNT (*g*), and PA (*h*) concentration in CH_2Cl_2, with an excitation wavelength of 325 nm for **P5** solution.

Table S4. Detailed data of the fluorescence lifetime measurements of **P1-P5** in DCM: τ – lifetime, *f* - fractional contribution, τ_{avg} – average lifetime, χ^2 - chi-squared distribution.

Compound	τ_1, [ns]	f_1, %	τ_2, [ns]	f_2, %	τ_{ave}, [ns]	χ^2
**P5 (2×10^{-7}M)**	1.09	98	2.73	2	1.12	1.197
**P5+DNT (1×10^{-4}M)**	0.99	86.4	1.65	13.6	1.08	1.142
**P5+DNT (2×10^{-4}M)**	1.04	94.1	1.96	5.9	1.10	1.069
**P5+DNT (3×10-4M)**	1.02	88.2	1.68	11.8	1.09	1.200
**P5+DNT (4×10^{-4}M)**	1.06	96.2	2.19	3.8	1.10	1.185

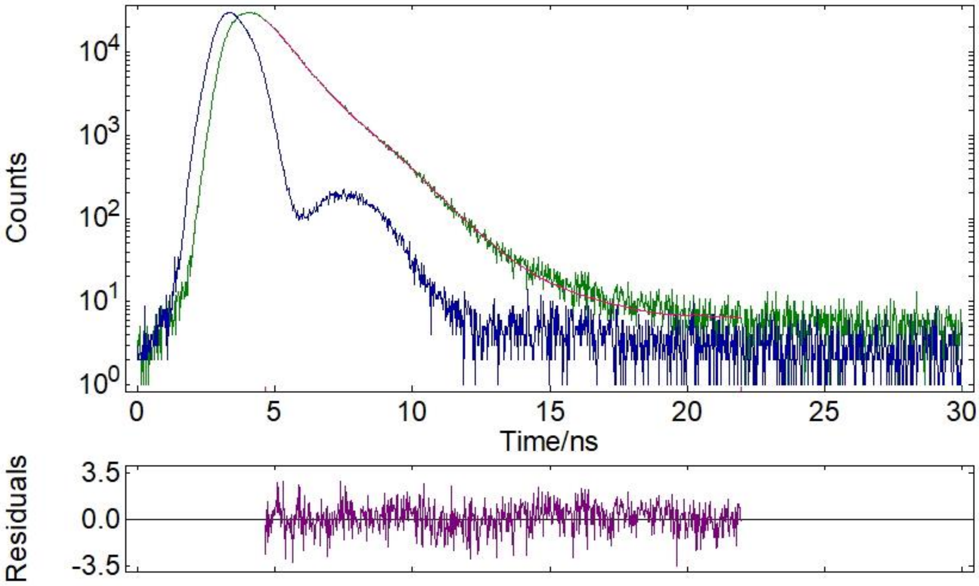

Figure S55. Time-resolved fluorescence lifetime decay profile of **P5** with DNT (1x10^{-4}M) (*blue*), IRF (*green*). λ_{ex} = 300 nm, λ_{ex} = 392 nm.

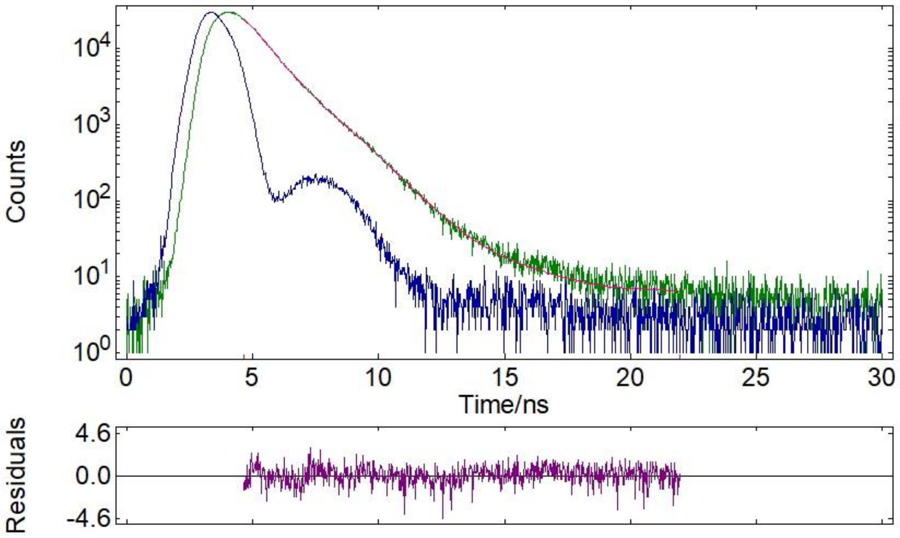

Figure S56. Time-resolved fluorescence lifetime decay profile of **P5** with DNT (2x10^{-4}M) (*blue*), IRF (*green*). λ_{ex} = 300 nm, λ_{ex} = 392 nm.

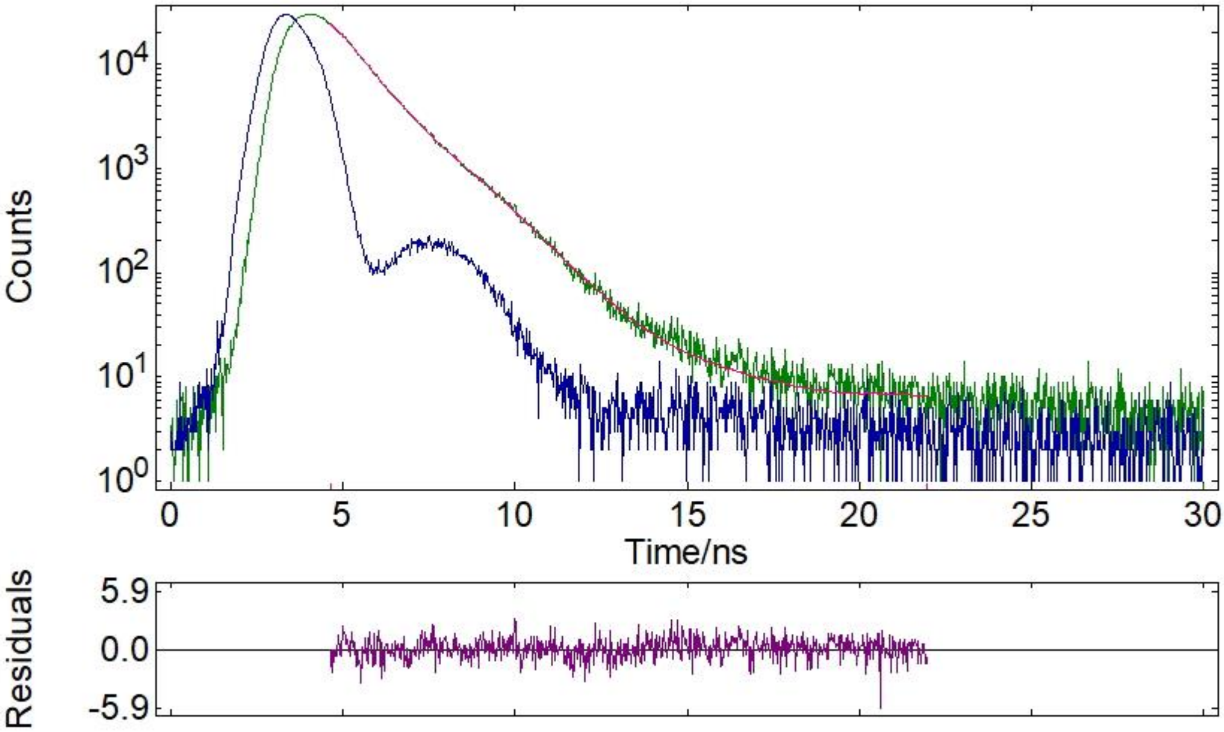

Figure S57. Time-resolved fluorescence lifetime decay profile of **P5** with DNT (3x10^{-4}M) (*blue*), IRF (*green*). λ_{ex} = 300 nm, λ_{ex} = 392 nm.

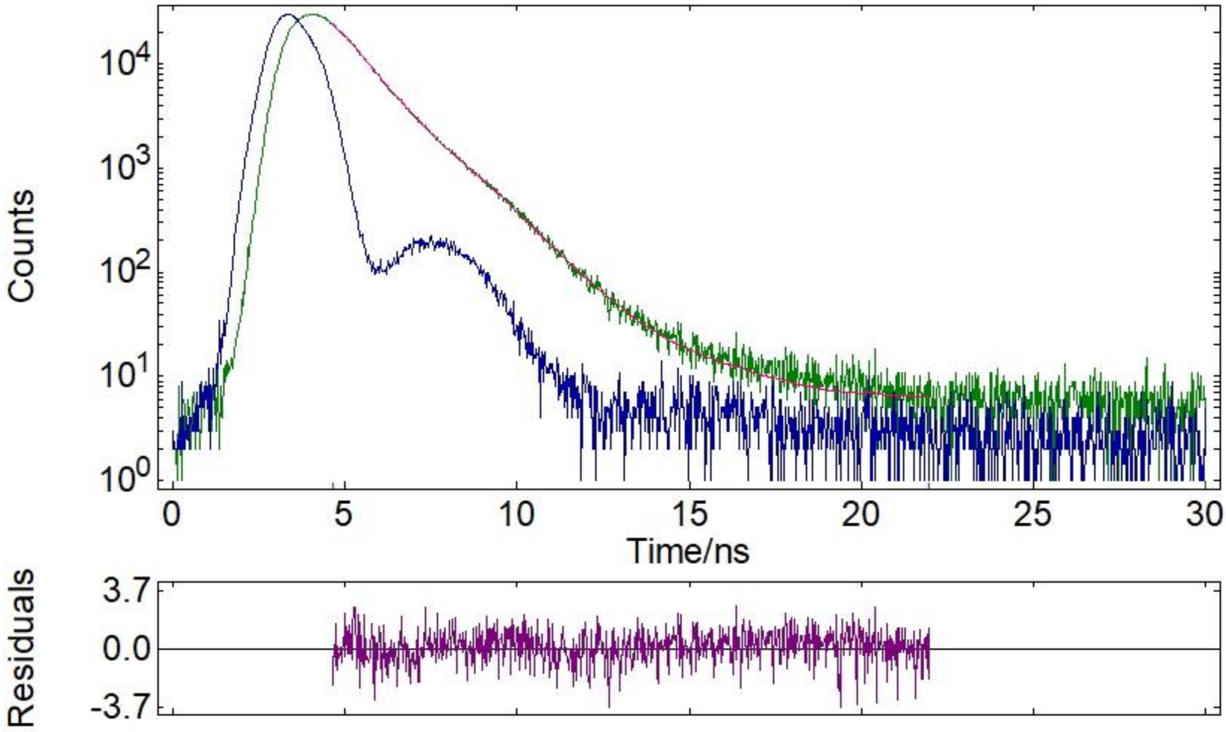

Figure S58. Time-resolved fluorescence lifetime decay profile of **P5** with DNT (4x10^{-4}M) (*blue*), IRF (green). λ_{ex} = 300 nm, λ_{ex} = 392 nm.

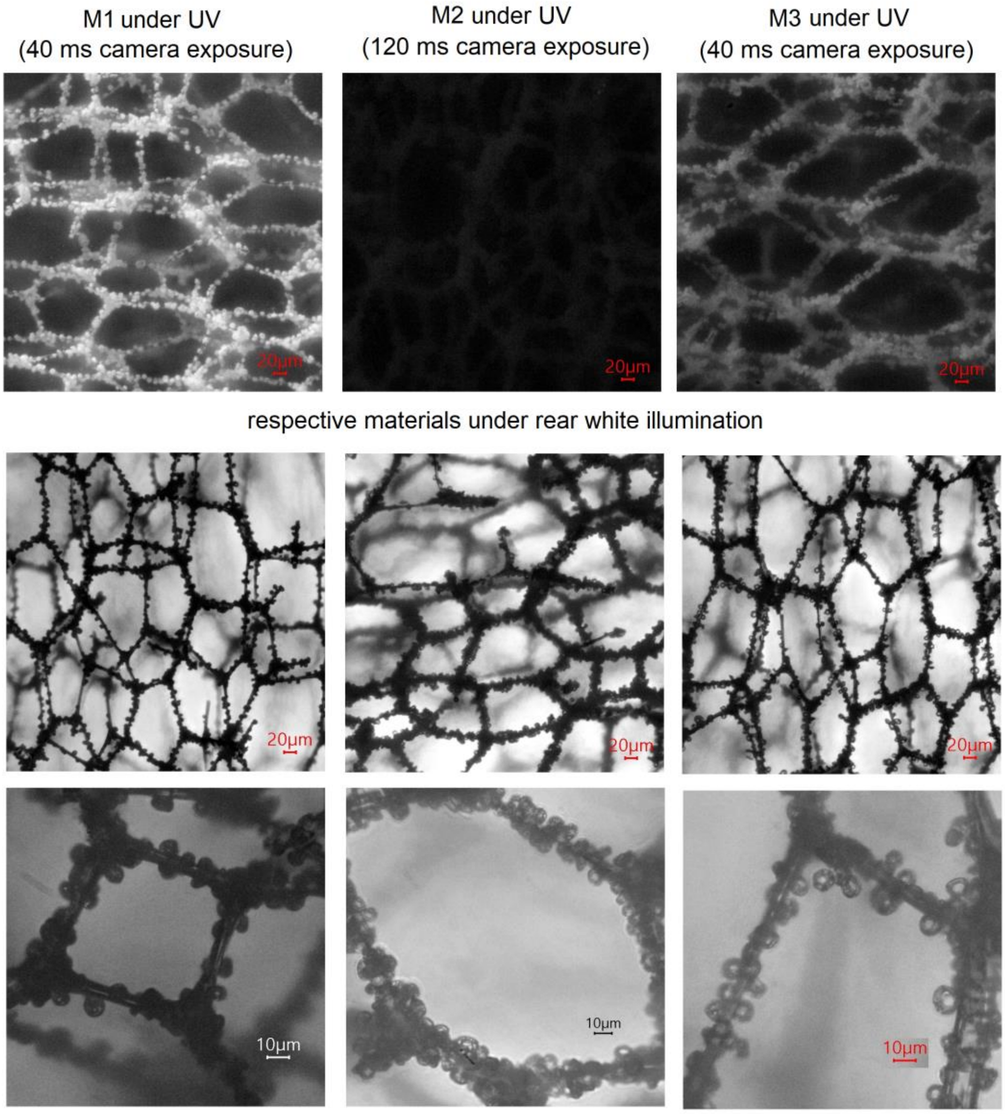

Figure S59. Photos of sensor materials **M1-M3** obtained by electrospinning of fluorescent polymers onto melamine-formaldehyde substrate. For shots under UV illumination, the source of illumination was fixed relative to the material.

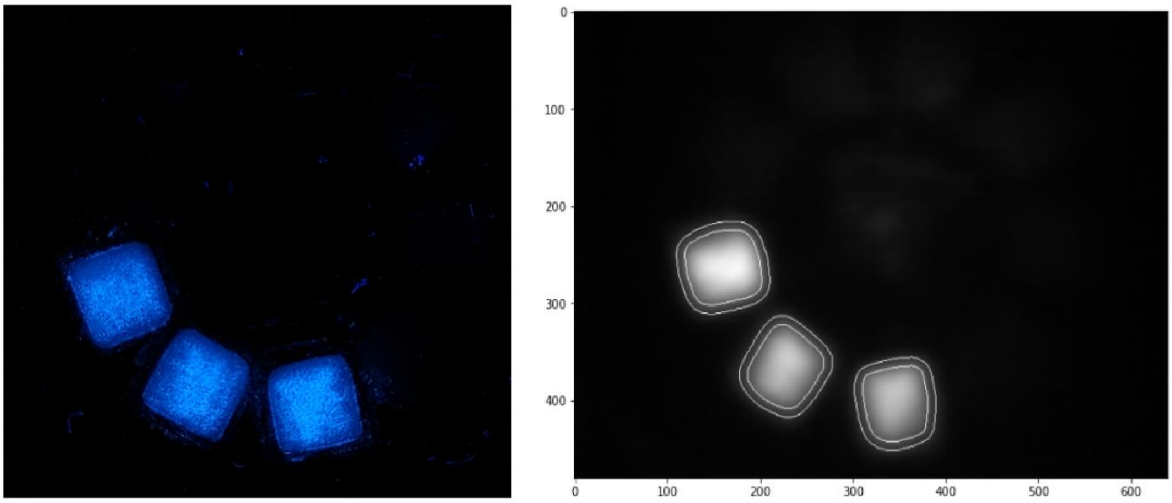

Figure S60. Photos of the typical sensor cartridge loadout used for measurements under UV illumination (365 nm) shot by the phone camera (*left*) and the camera of the fluorescence recorder (*right*).

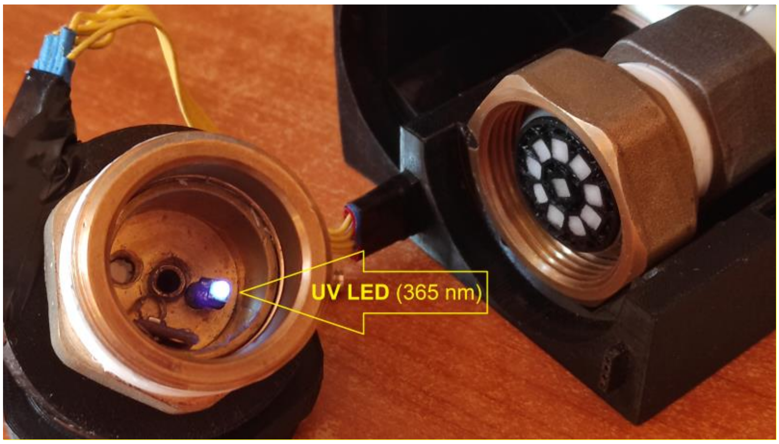

Figure S61. The optical scheme of fluorescence recorder. The device has disassembled for the sensor installation.

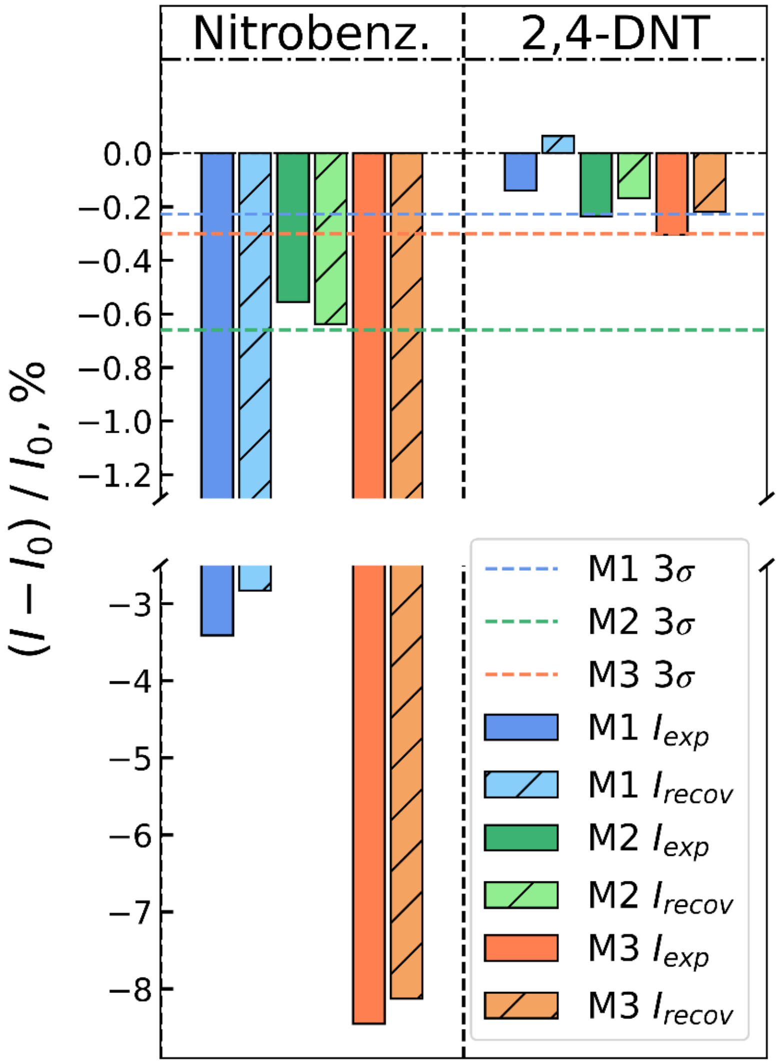

Figure S62. Fluorescence responses of sensor materials to NB and DNT vapors at a concentration of 10% from saturated one in a 100 sec exposure interval. The 3σ noise levels of registered fluorescence intensity difference are marked by horizontal dashed lines.

Table S5. Detection times of obtained sensor materials towards nitroaromatic vapours when applied in the fluorescence recorder. Saturated vapor concentrations at 25C

Material	Analyte	Concentration	Source of vapours	Time to detect
**M1**	NB	347.8 ppm	160 mL pulse of saturated vapour	1.8 sec
**M1**	DNT	282.4 ppb	160 mL pulse of saturated vapour	3.0 sec
**M1**	TNT	5.8 ppb	160 mL pulse of saturated vapour	14.6 sec
**M1**	PA	943.6 ppt	160 mL pulse of saturated vapour	Not detected in 50 sec
**M2**	NB	363.5 ppm	160 mL pulse of saturated vapour	2.1 sec
**M2**	DNT	396.1 ppb	160 mL pulse of saturated vapour	Not detected in 50 sec
**M2**	TNT	5.8 ppb	160 mL pulse of saturated vapour	Not detected in 50 sec
**M2**	PA	943.6 ppt	160 mL pulse of saturated vapour	Not detected in 50 sec
**M3**	NB	363.5 ppm	160 mL pulse of saturated vapour	2.8 sec
**M3**	DNT	386.1 ppb	160 mL pulse of saturated vapour	4.6 sec
**M3**	TNT	5,8 ppb	160 mL pulse of saturated vapour	Not detected in 50 sec
**M3**	PA	637.2 ppt	160 mL pulse of saturated vapour	Not detected in 50 sec
**M1**	NB	28 ppm	Continuous stream	7.2 sec
**M1**	NB	1 ppm	Continuous stream	239 sec
**M1**	DNT	26.3 ppb	Continuous stream	Not detected in 100 sec
**M1**	TNT	532.7 ppt	Continuous stream	Not detected in 100 sec
**M2**	NB	35.7 ppm	Continuous stream	Not detected in 100 sec
**M3**	NB	28.7 ppm	Continuous stream	5.7 sec
**M3**	NB	1 ppm	Continuous stream	247 sec
**M3**	DNT	25.2 ppb	Continuous stream	98.7 sec

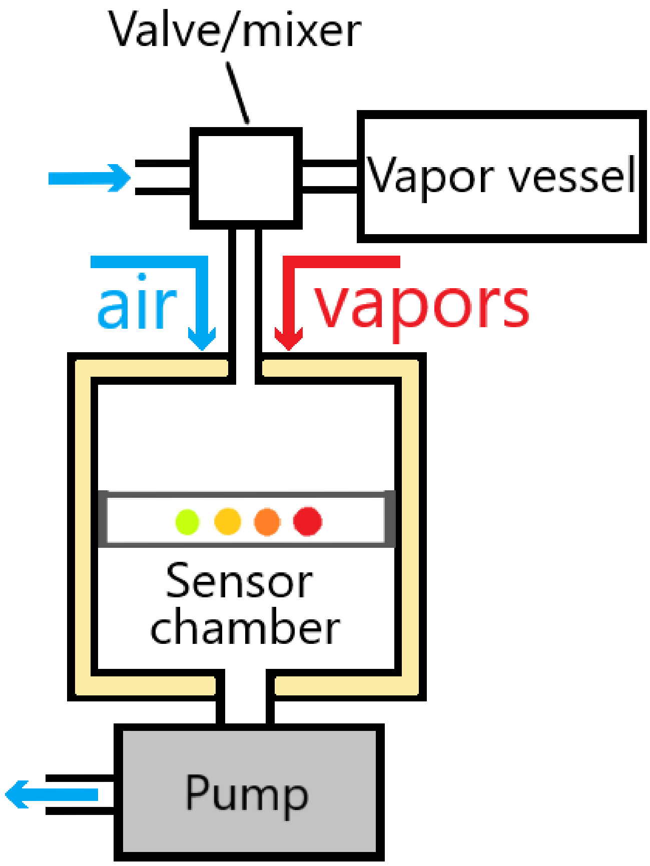

Figure S63. The scheme of vapor measurements setup.

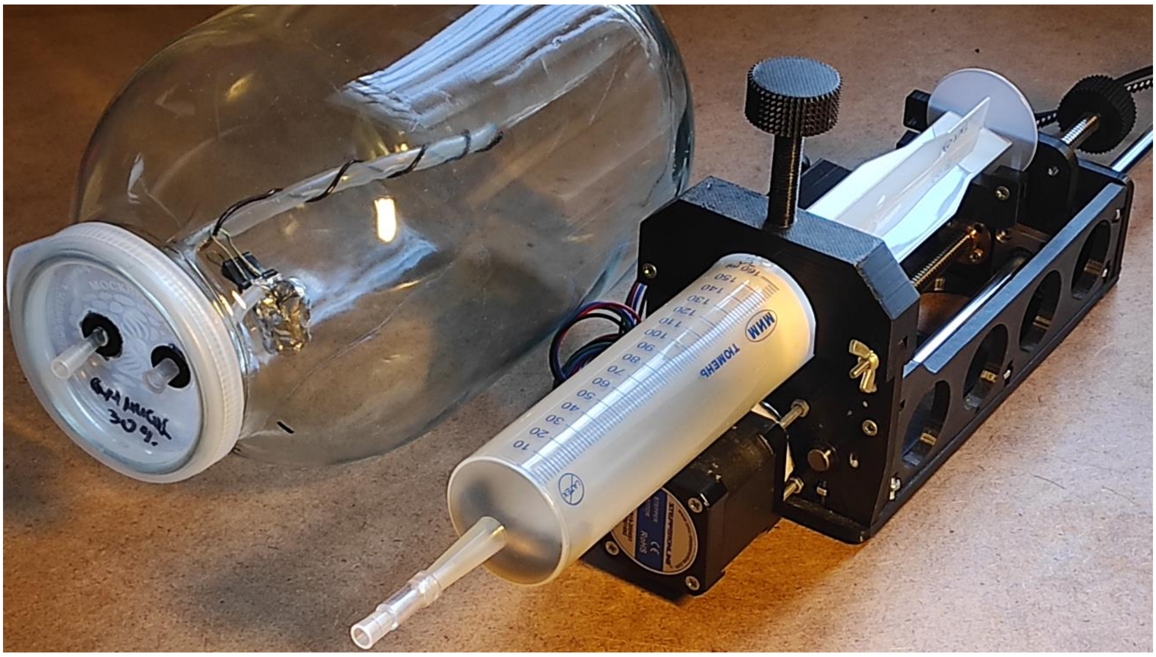

Figure S64. Vapor vessels, the 3 L glass vessel on the left, and the 160 mL syringe vessel installed onto the syringe driver on the right.
